# Readily Accessible and Brightly Fluorogenic BODIPY/NBD–Tetrazines via S_N_Ar Reactions

**DOI:** 10.1021/acs.joc.3c02864

**Published:** 2024-04-10

**Authors:** Murat Işık, Mehmet Ali Kısaçam

**Affiliations:** † Department of Food Engineering, Bingöl University, 12000 Bingöl, Türkiye; ‡ Department of Biochemistry, Faculty of Veterinary Medicine, Mustafa Kemal University, 31060 Hatay, Türkiye

## Abstract

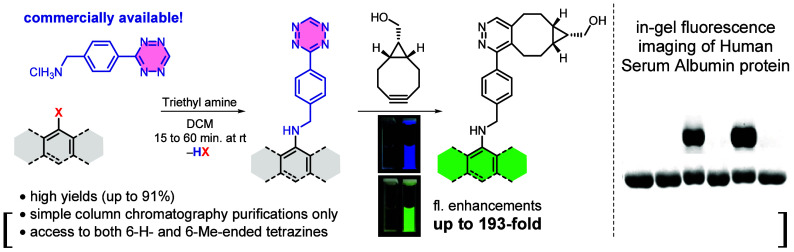

We describe S_N_Ar reactions of some commercial amino-tetrazines and halo-dyes, which give efficiently quenched BODIPY/NBD–tetrazines (*Φ*_Fl_ < 0.01) in high yields and, importantly, with high purities affordable via simple silica gel chromatography only. The dyes exhibit large Stokes shifts, moderate environmental sensitivity, and emission enhancements (up to 193-fold) upon Tz ligation with BCN—a strained dienophile. They successfully serve as labels for HSA protein premodified with BCN, resulting in bright blue–green emission upon ligation.

Bioorthogonally activatable fluorescence turn-on probes have become invaluable tools for visualizing and tracking biological molecules and processes.^1^ The tetrazine (1,2,4,5-tetrazine or Tz) ligation, in this regard, has been considered one of the most promising bioorthogonal reactions developed to date primarily because it obviates the need for a catalyst, unlike the emblematic azide–alkyne “click” reactions where catalysis by cytotoxic copper species is required for reasonable reaction rates.^2^ In addition, among strain-promoted bioorthogonal reactions,^3^ inverse electron demand Diels–Alder (iedDA) reactions^4,5^ of tetrazines and strained dienophiles stand out with their exceptional reaction kinetics.^6,7^

Another advantage of using tetrazines as bioorthogonal handles lies in their outstanding quenching abilities, which facilitate the development of high-contrast imaging tools.^6,8^ One of the earliest methods for synthesizing fluorophore–tetrazine (Fl–Tz) dyes entails classical Pinner-like approaches which allow for installing tetrazine nuclei directly onto nitrile-bearing fluorophores (see Figure 1A).^9^ While these methods are particularly notable for bringing the Fl and Tz moieties into close proximity and thus enable powerful fluorescence quenching,^10,11^ they acknowledge several inconveniencies: (1) Syntheses involving excessive use of hydrazine require the addition of nitrite, an oxidizer, to the reaction mixture, posing a risk of explosion.^12−15^ (2) The reactions often give hardly separable, monosubstituted (6-H-substituted) Tz byproducts in addition to the often desired 6-Me-substituted targets, frequently both in comparable, low yields. This may require reversed-phase *preparative* HPLC purifications to afford the dyes with sufficient purity.^10,11,16^

**Figure 1:**
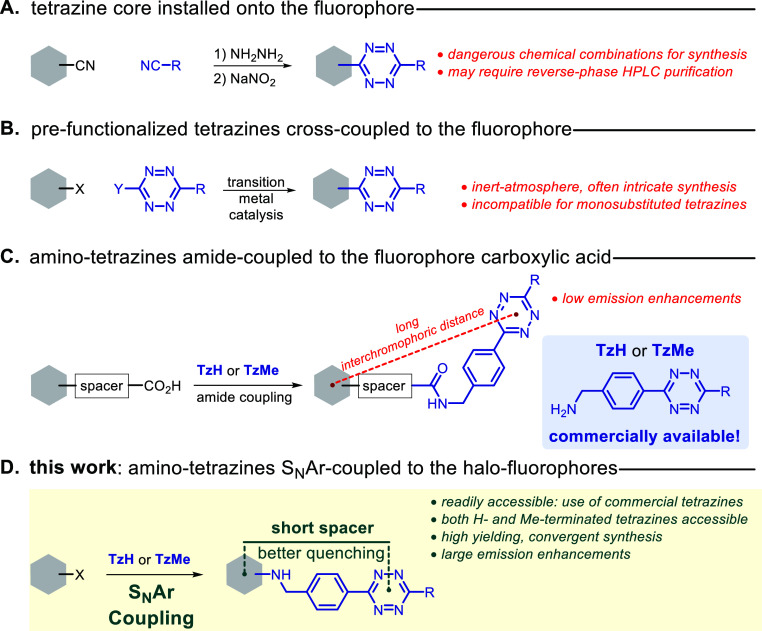
(A–C) The most common synthetic approaches toward Fl–Tzs. (D) S_N_Ar coupling described herein.

Transition metal catalysis (TMC)-based approaches to introduce Tz units into fluorophores (Figure 1B), on the other hand, generally offer milder reaction conditions than the Pinner approach and give Fl–Tz constructs in higher yields with relatively simpler purifications.^17−22^ While these efforts, together with the aforementioned Pinner strategy, have been transformative to the field, the frequently encountered exhaustive syntheses, often requiring inert atmosphere conditions, considerably limit their accessibility.

A third major approach toward Fl–Tzs involves conventional carboxy-amide coupling of commercially available aminotetrazines, most commonly TzH or TzMe (Figure 1C), with carboxyl-functionalized fluorophores, and *vice versa*. The amide-coupling strategy, first reported by Devaraj and co-workers, stands as the earliest method for producing fluorogenic Tz dyes.^23^ Unlike the Pinner and TMC approaches, this method enables Tz ligation chemistry more accessible to a wider audience thanks to the commercial availability of reacting partners and their straightforward reactions.^24−28^ However, the Fl–Tz dyes thus produced are usually only moderately fluorogenic due, presumably, to large spacers intervening between the fluorophore and the (quencher) Tz (Figure 1C).^23−29,10^

We also appreciate leveraging the commercial availability of TzH and TzMe at reasonably affordable prices^30^ to provide access to new Fl–Tz dyes with enhanced fluorescence behavior. However, we reasoned that they could be harnessed more effectively than through carboxy-amide-coupling when coupled with halo-fluorophores via a nucleophilic aromatic substitution (S_N_Ar) reaction (Figure 1D). Such a maneuver minimizes the interchromophoric distance between the Fl and Tz, which would result in better quenching.^31,32^ Recently, the S_N_Ar strategy,^31,33−35^ among others,^36,37^ has emerged as an enabling platform for the development of several highly fluorogenic tetrazines. In this Note, we report that these commercial amino-tetrazines can seamlessly couple with some commercial (or otherwise readily prepared; see the Supporting Information) halo-fluorophores (Fl–X) via room-temperature S_N_Ar reactions to give several efficiently quenched Fl–Tz conjugates in high yields.

We chose (*pseudo*)-halogen-substituted boron dipyrromethenes (BODIPYs: **1a**–**b**,^38−41^**2a**–**c**^42−44^)^45,46^ and nitrobenzofurazan (NBD: **3a**–**b**)^47,48^ dyes as electrophilic S_N_Ar partners for this study (Scheme 1), primarily because of their well-established, high reactivity toward nitrogen nucleophiles at mild reaction conditions.^49−52^ We set out to react all-commercially available **1a** with TzH in dichloromethane at room temperature (entry 1, Table 1) in the presence of the ubiquitous organic base, triethylamine (TEA).^53^

**Scheme 1:**
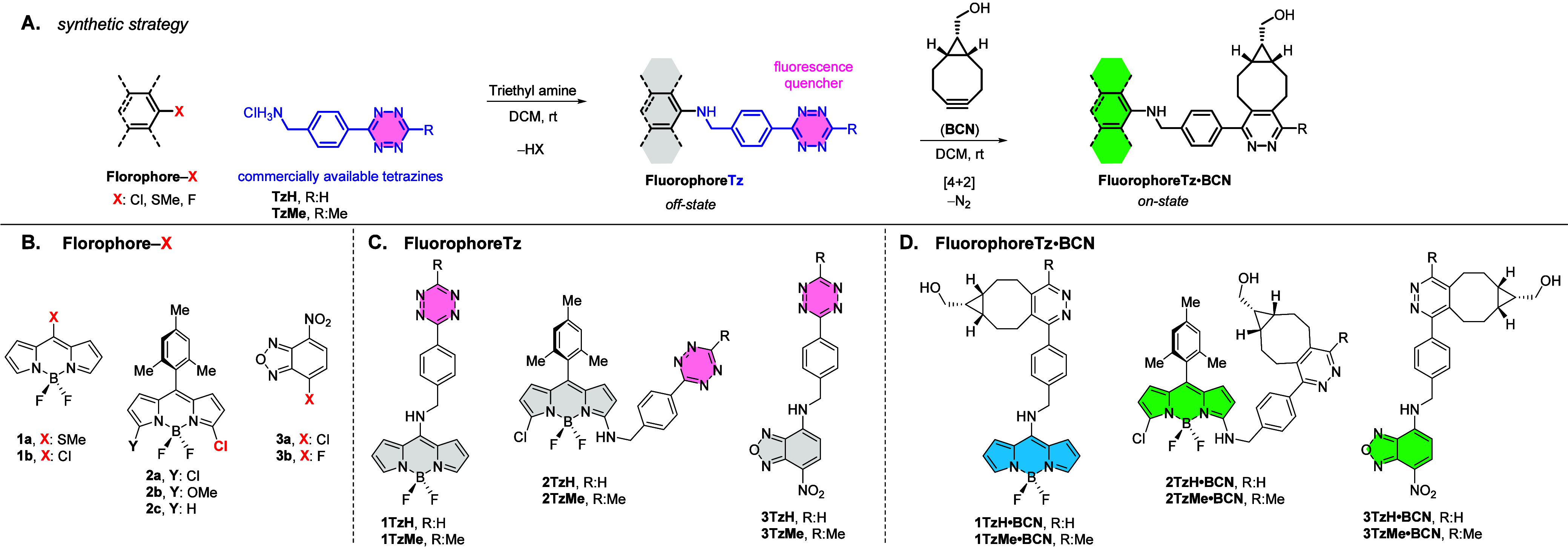
Synthesis of Fluorophore–Tetrazine Dyes and Their iedDA Reactions with BCN (A) The S_N_Ar strategy adopted. (B) (*pseudo*)halo-fluorophores tested. (C) Fl–Tz dyes described herein. (D) iedDA adducts of Fl–Tz with BCN.

**Table 1:** Synthesis of Fl–Tzs^a^ and Their iedDA Adducts with BCN^b^

entry	Fl–X	Fl–Tz	time [h] S_N_Ar	yield [%] S_N_Ar	time [min] iedDA	yield [%] iedDA
1	**1a**(**1b**)	**1TzH**	1(0.5)	59(63)	5	92
2	**1a**(**1b**)	**1TzMe**	1(0.5)	85(77)	30	71
3	**2a**	**2TzH**	1	64	5	90
4	**2a**	**2TzMe**	1	91	30	85
5	**3b**	**3TzH**	0.25	41	5	94
6	**3b**	**3TzMe**	0.25	75	30	95

a Reaction conditions: TzH or TzMe, 1.1 equiv of Fl–X, 2.0 equiv of TEA. For **1a**, 1.0 equiv of TEA was used.

b Reaction conditions: Fl–Tz, 1.5 equiv of BCN.

Gratifyingly, when carried out in either 10- or 25-mg vials of commercially packaged TzH, the S_N_Ar reaction was completed in 1 h giving **1TzH** as a maroon solid in reasonable yield (59%). The product was easily purified solely by means of silica gel chromatography, without the need for any sophisticated equipment.^54^ Delightedly, this nonfluorescent maroon solution of **1TzH** immediately turned pale yellow with an accompanied bright blue emission upon adding some excess of BCN.^55^ Encouraged by this result, we also subjected TzMe to this reaction under identical conditions and isolated the product **1TzMe** with a much higher yield than **1TzH** (85%, entry 2, Table 1). To enhance the chemical yield of **1TzH**, we explored an alternative, benign substrate: 8-chloroBODIPY (**1b**),^41^ but substituting **1a** with **1b** resulted in similar chemical yields.

Next, we wanted to show that this chemistry is not restricted to blue-emissive BODIPYs. To this end, we prepared several α-chloro-BODIPY compounds **2a**–**c** following the literature protocols (Figure 1S).^42−44^ Among these, while the 3,5-bischlorinated-BODIPY dye (**2a**) coupled readily and very efficiently to the Tzs (entries 3–4, Table 1), the 3-chloro-5-methoxy-BODIPY derivative (**2b**) likely formed a BODIPY–Tz conjugate. However, despite our efforts, we were unable to characterize its structure. Last, *α-*monochloro-BODIPY (**2c**)^44^ proved to be completely unreactive toward these Tzs, even at elevated temperatures (refluxing in dichloromethane or acetonitrile). Because the synthesis of **2a** required kinetically controlled chlorination at −78 °C with tedious dropwise addition of the reagent in the dark,^43^ we also proposed all-commercially available potential surrogates, namely, NBD-Cl (**3a**) and NBD-F (**3b**). While compound **3a** did not yield any S_N_Ar products with either of the Tzs, fluoro-derivative (**3b**) reacted very well, similar to the BODIPY-based precedents (entries 5–6, Table 1). In general, the S_N_Ar reactions of Fl–X with TzMe occur more efficiently than those with TzH, consistently resulting in yields ca. 25% higher. This trend holds true regardless of the type of fluorophore or the identity of the leaving group, suggesting that the lower yields obtained in the reactions with TzH should result from its inherent instability in an alkaline medium.

We have employed BCN dienophile exclusively since its cycloadducts (Fl–Tz·BCN) possess a well-defined stereochemistry, which facilitates drawing conclusions.^25^ The cycloaddition reactions involving H-terminated Tzs were completed cleanly within 5 min, while those involving Me-terminated Tzs took longer (30 min) to complete (entries 1–6, Table 1), as expected.^56^

All of the Fl–Tz dyes and their BCN adducts consistently displayed a mild solvatochromism with large Stokes shift (*ca*. 50–65 nm; specifically, ∼2.050–2.800 cm^–1^ for BCN adducts) across solvents of varying polarity (Table 1S and Figures 4S and 5S). This chromic behavior arises from the push–pull structure (from the amino group to the fluorophore), resulting in internal charge transfer (ICT) transitions.^40,57^ Unlike typical BODIPYs, which usually have very small Stokes shifts (400–600 cm^–1^),^49,50^ the fluorophores described herein with their large Stokes shifts would overcome self-quenching often observed in the former, especially when densely populated (e.g., on a biomolecule or polymer).^58^ Since the Fl and the Tz chromophores are electronically decoupled, the band maxima of the absorption or emission spectra of Tz dyes and their cycloadducts remain unchanged (Table 1S, Figures 4S and 5S, and Figure 2J). In general, while BODIPY-based probes and their cycloadducts, both the 3-amino and 8-amino substituted variants, exhibited blue shifts with increasing solvent polarity, whereas NBD derivatives, conversely and complementarily, showed red shifts.

**Figure 2:**
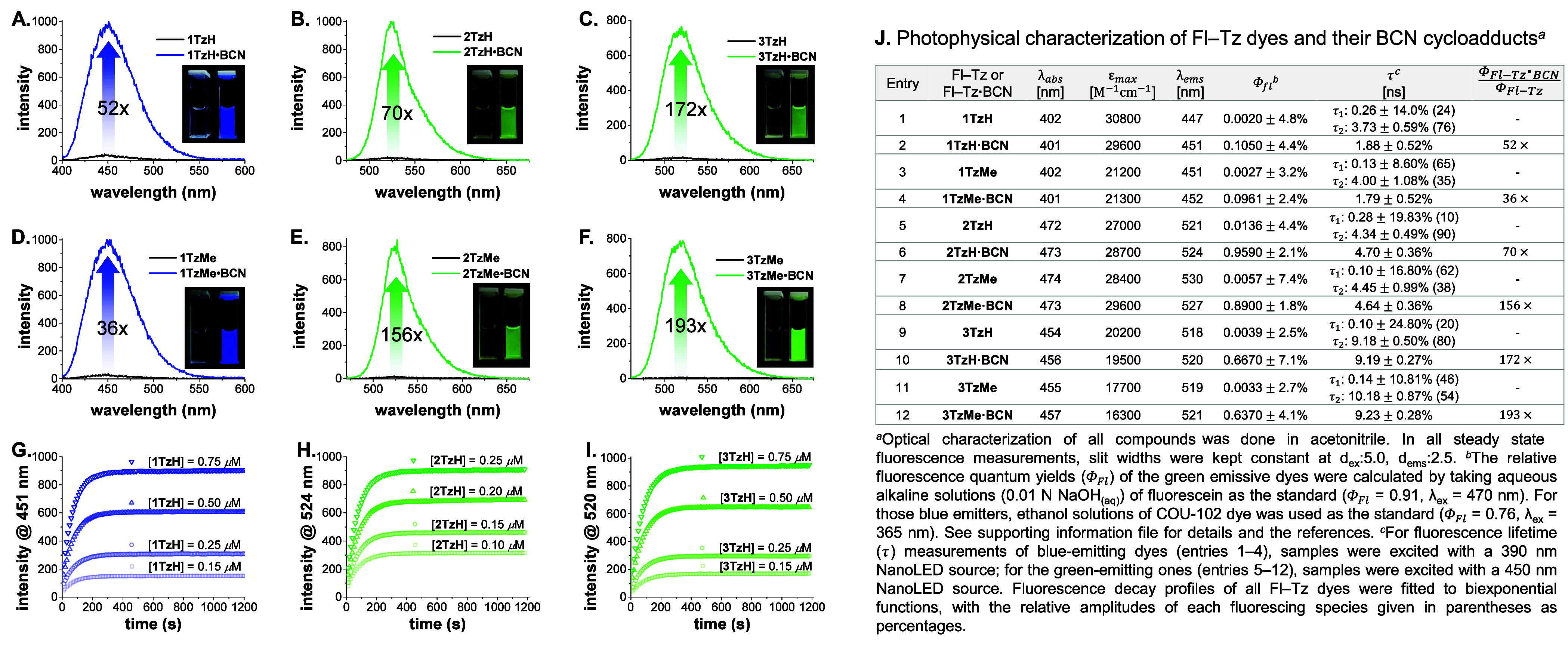
(A–F) Fluorescence emission comparison of Fl–Tz dyes and their BCN adducts. (G–I) Time-dependent emission profiles of iedDA reactions of Fl–Tz dyes (at varying conc.) and BCN (0.1 mM) in acetonitrile with 1% DMSO. (J) Table for photophysical characterizations.

The fluorescence quantum yields (*Φ*_Fl_) of all Fl–Tzs were notably less than 1%, which presented a fairly dark stage for fluorogenic Tz ligation reactions (Figure 2J). Delightedly, cycloaddition reactions dramatically restored fluorescence emissions (36× to 193× enhancements) leading to bright blue–green emissive solutions. The dyes’ molar absorptivities (*ε*_max_: 1.6–3.0 × 10^4^ M^–1^ s^–1^) unaltered upon ligation, combined with high fluorescence turn-on ratios, highlight their brightness (ε × *Φ*_Fl_, Table 2S).^58^ Fluorescence enhancements were more pronounced for the green emitters (70× to 193×) regardless of the fluorophore or tetrazine scaffold, compared to the blue emitters (36× and 52×).

To gain further insight into quenching, fluorescence lifetimes (τ) of each dye were determined through time-correlated single-photon counting experiments. The fluorescence decay profiles of each cycloadduct followed a single exponential function, while their Tz forms consistently exhibited biexponential decay models in all cases (Figure 6S and Figure 2J). The biexponentially fitted lifetimes of Tz dyes are all characterized by one fast-decaying component (τ_1_: 0.1–0.3 ns) accompanied by a slow decaying one (τ_2_: 3.7–10.2 ns), clearly indicating the presence of two distinct decaying species within the excited state. In light of the photophysical characterization data gathered, we speculate that FRET (Förster resonance energy transfer) would be the most likely quenching mechanism for our electronically separated, bichromophoric Fl–Tz dyes.^29^ We arrive at this conclusion particularly because the quenching efficiency is apparently wavelength-dependent (entries 1–4 vs entries 5–12, Figure 2J).^59^ Moreover, another indication of quenching by FRET is the shortening of the fluorescence lifetime of the donor,^60^ that is evident from the fast-decaying components of the decay models of Fl–Tzs (Figure 2J).

Interestingly, pH variations had no noticeable impact on either the absorbance or emission maxima, nor on their respective intensities (Figures 7S and 8S). Nonetheless, compared to their acetonitrile solutions, the emission intensities were substantially reduced to a quarter or half due to the aquatic medium, which further underscored their environmental sensitivity.^61^ These findings signify the suitability of these dyes for use in diverse biological contexts, particularly in situations where pH and polarity fluctuations can be substantial.

The rate constants (*k*_2_) of iedDA reactions are calculated as follows: 129.8 ± 6.6 M^–1^ s^–1^ for **1TzH**·**BCN**, 122.0 ± 2.5 M^–1^ s^–1^ for **2TzH**·**BCN**, and 117.3 ± 5.6 M^–1^ s^–1^ for **3TzH**·**BCN**, respectively. These values are comparable to those of “copper-catalyzed” azide–alkyne click reactions (10–100 M^–1^ s^–1^)^62^ and to those precedent Tz ligations with matching bioorthogonal handles (2–4000 M^–1^ s^–1^)^18^ (Figure 2G–I and Figure 9S).

Finally, we wanted to evaluate H-ended Tz probes in protein labeling (Figure 3) due to their advantageous properties in this regard, specifically their minimal size, charge neutrality, and high click reactivity.^63−65^

**Figure 3:**
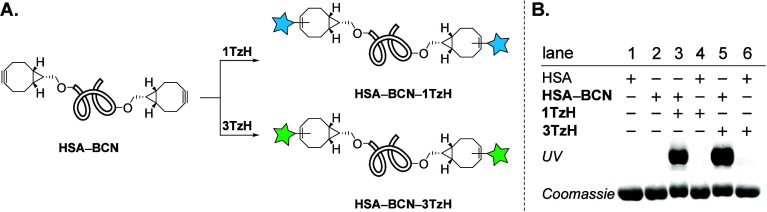
(A) Labeling of HSA–BCN conjugate with H-terminated tetrazine dyes (**1TzH** and **3TzH**.) (B) In gel (SDS-PAGE) visualization of labeling experiments including negative controls. Digital photographs of fluorescent bands (top) and Coomassie Blue stained gel (bottom).

Toward this, a high-purity (≥99%, lyophilized) human serum albumin (HSA) was premodified with BCN chemical reporters at its lysine residues (−NH_2_).^66^ Both this **HSA–BCN** conjugate (see the Supporting Information for preparation) and the negative control (HSA) were separately incubated with one blue-emitting (**1TzH**) and one green-emitting (**3TzH**) probe in the dark at 37 °C for 1 h. The resulting protein solutions were spin-filtered and subsequently analyzed by sodium dodecyl sulfate–polyacrylamide gel electrophoresis (SDS-PAGE) (Figure 3B). Strongly fluorescent bands were clearly visible exclusively in lanes 3 and 5, involving the incubation of merely **HSA–BCN** and the tetrazine probes. The absence of any fluorescence signal from the negative controls suggests no nonspecific binding of these probes, thereby confirming that the signal arises solely from the highly chemoselective reactions occurring between the chemical reporters (BCN) deployed on the protein and the Tz probes. Protein bands, thereafter, were also visualized by the nonspecific Coomassie blue stain.

In conclusion, by taking advantage of the commercial availability of some amino-Tzs (TzH and TzMe) and halo-fluorophores, we have described an S_N_Ar protocol that allows the ready preparation of several novel BODIPY/NBD–Tetrazine probes in high yields and purities. The dyes are characterized with large Stokes shifts, moderate environmental sensitivity, and high fluorescence turn-on ratios in Tz ligation with BCN. We have shown successful use of these probes in labeling of cyclooctyne-modified HSA protein. Given their convenient synthesis, purification, and high fluorogenicity, we hope that the Tz probes developed herein would help democratize the *elite* fluorogenic Tz ligation chemistry and represent examples along this road.

## Data Availability

The data underlying this study are available in the published article and its online Supporting Information.

## Supporting Information Available

Synthetic procedures, characterization data, copies of NMR spectra, HRMS spectra, IR spectra, and additional spectroscopic results (PDF)
FAIR data, including the primary NMR FID files, for compounds **1TzH**, **2TzH**, **3TzH**, **1TzMe**, **2TzMe**, **3TzMe**, **1TzH**·**BCN**, **2TzH**·**BCN**, **3TzH**·**BCN**, **1TzMe**·**BCN**, **2TzMe**·**BCN**, and **3TzMe**·**BCN** (ZIP)

## Dedication

In memoriam to Dr. Mehmet Ali Kısaçam, our esteemed colleague, who passed away in the 2023 Kahramanmaraş (Türkiye) earthquake. We are grateful for his contributions to this work.
