# Supplementary material for: Readily Accessible and Brightly Fluorogenic BODIPY/NBD–Tetrazines via SNAr Reactions
Source: J Org Chem. 2024 Apr 10;89(9):6513–9. doi: 10.1021/acs.joc.3c02864 (PMC11077493; doi:10.1021/acs.joc.3c02864)
Supplement: Supplementary file 1 — jo3c02864_si_001.pdf [file jo3c02864_si_001.pdf]

Supporting Information

for

Readily Accessible and Brightly Fluorogenic BODIPY/NBD–Tetrazines via SNAr Reactions

Murat Işık*,† and Mehmet Ali Kısaçam‡

†*Department of Food Engineering, Bingöl University, Bingöl, 12000, Türkiye*
‡*Department of Biochemistry, Faculty of Veterinary Medicine, Mustafa Kemal University, 31060 Hatay, Türkiye*

*Correspondence to: misik@bingol.edu.tr

Table of Contents

General S2

Synthesis of halo-fluorophores (Fl–X) S3

Characterization data for halo-fluorophores and their precursors S3

General procedure for the synthesis of BODIPY/NBD-Tetrazines S8

General procedure for inverse electron demand Diels–Alder reactions: S11

  Variable temperature ^{1}H-NMR spectra of 2TzH in $CDCl_3$ S14

Solvatochromism of Fl–Tz or Fl–Tz•BCN dyes S15

  Stokes shifts of Fl–Tz•BCN dyes in acetonitrile S15

  Solvent screening studies of Fl–Tz and Fl–Tz•BCN dyes S16

  Solvent screening spectra of Me-ended Fl–Tz and Fl–Tz•BCN dyes S18

Fluorescence decay profiles of Fl–Tz and Fl–Tz•BCN dyes S20

pH Dependency of Fl–Tz and Fl–Tz•BCN dyes S21

Time-dependent fluorescence emission of 1TzMe S23

Protein labelling studies S29

  Synthesis and characterization of the amine-reactive BCN–PNP S29

  BCN modification of HSA by BCN–PNP S29

Copies of NMR, HRMS and FTIR spectra S31

References S82

General

(*pseudo*)Halo-BODIPY dyes (**1a**, **1b**, **2a**–**2c**)[1–6] were synthesized following the literature cited. Halo-NBD dyes (**3a** and **3b**) were supplied from TCI chemicals. Tetrazines (**TzH**: (4-(1,2,4,5-tetrazin-3-yl)phenyl)methanamine hydrochloride and **TzMe**: (4-(6-methyl-1,2,4,5-tetrazin-3-yl)phenyl)methanamine hydrochloride) were supplied both in quantities of 10 or 25mg from Sigma-Aldrich (Merck), or in quantities of 100mg/500mg from Conju-Probe. Racemic (1*R*,8*S*,9*s*)-bicyclo[6.1.0]non-4-yn-9-ylmethanol (**BCN**) was commercially supplied from Sigma-Aldrich (Merck). **BCN**–**PNP** was prepared following the literature report.[7] All other chemicals and solvents used in this work were commercially supplied and used without further purification unless specifically noted. 1D NMR spectra were recorded on a Bruker Spectrospin Avance DPX 400 spectrometer using $CDCl_3$ and DMSO-d_6 as the solvent. High resolution NMR spectra were acquired on an Agilent-Premium Compact (600 MHz, 14.1 Tesla). Chemical shifts values are reported in ppm relative to tetramethylsilane as the internal standard. Spin multiplicities are reported as follows: s (singlet), bs (broad singlet), d (doublet), t (triplet), dd (doublet of doublet), ddd (doublet of doublet of doublet) and m (multiplet). HRMS data were acquired on an Agilent Technologies 6530 Accurate-Mass Q-TOF LC/MS. UV-Vis Absorption spectra were taken on a Shimadzu UV-3101PC UV–VIS-NIR spectrophotometer. Fluorescence measurements were recorded on a Perkin–Elmer (Model LS 55) spectrofluorometer. A Horiba Jobin-Yvon Time-Resolved Fluorometer, Fluorolog FL-1057, equipped with HORIBA NanoLED light sources (NanoLED-390 and NanoLED-450), were used for the fluorescence decay experiments. FT-IR spectra of new compounds were obtained on a Perkin Elmer 100 model FTIR spectrometer (ATR). Fluorescence images of SDS-PAGE gels were acquired on an iBright CL-1000 Imaging System. The BCN-tagged proteins were purified by Zeba™ Spin Desalting Columns (40K MWCO). Protein samples were electrophoresed on a Mini Gel Tank (Invitrogen). Flash grade silica gel (SiliaFlash Irregular Silica Gels, F60, 40–63 µm, 60 Å) was used for flash column chromatography (FCC) purifications. Reactions were monitored by thin layer chromatography (TLC) using precoated silica gel plates (Merck Silica Gel PF-254), visualized by a handheld UV-Vis lamp. All organic extracts were dehydrated over either anhydrous Na_2SO_4 or $MgSO_4$ and concentrated by using rotary evaporator before being subjected to FCC. For all absorption and emission spectra recordings a quartz cell with 1.0 cm pathlength was used. For each measurement 2.0 mL aliquots were used. The relative fluorescence quantum yields (Φ_{Fl}) of the green emissive dyes were calculated by taking aqueous alkaline solutions (0.01 N $NaOH_{(aq)}$) of fluorescein as the standard (Φ_{Fl} = 0.91, λ_{ex} = 470 nm).[8] For those blue emitters, ethanol solutions of COU-102 dye were used as the standard (Φ_{Fl} = 0.76, λ_{ex} = 365 nm).[9] Following equation was used to calculate Φ_{Fl}:

$$\Phi_s = \Phi_r\left(\frac{m_s}{m_r}\right)\left(\frac{n_s}{n_r}\right)^2$$

Where Φ denotes fluorescence quantum yield, m: gradient of the plot of integrated fluorescence intensity against absorbance, n: refractive index of solution, subscripts r and s denotes

reference and sample, respectively. Optically dilute solutions were utilized for Φ_{Fl} measurements. For tetrazine dyes, 5 μM solutions were employed, while for BCN adducts, solutions of ≤1 μM were used. Solvents used were of spectroscopic grade, and all spectra were blank-corrected. The reported Φ_{Fl} results represent the mean of duplicates.

Synthesis of halo-fluorophores (Fl–X)

The synthetic routes we followed to obtain (*pseudo*)Halo-BODIPY dyes (**1a**,[1] **1b**,[2,3] **2a**,[4,5] **2b**,[5] **2c**[6]) is given below in Figure 1S. All these compounds and the related intermediate compounds were all thoroughly characterized with ^{1}H and ^{13}C NMR, HRMS, and IR spectroscopic analysis.

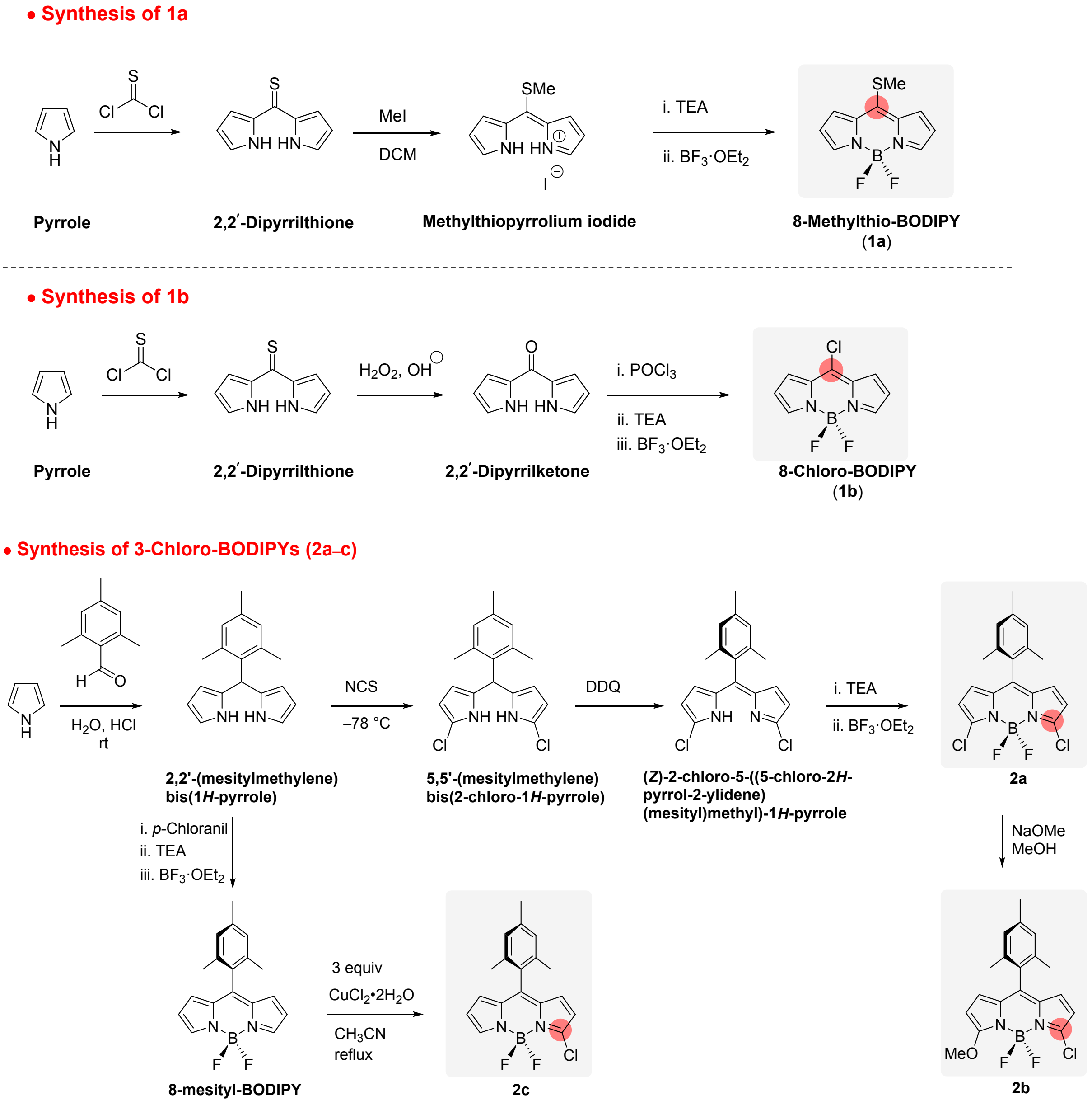

Figure 1S. Synthetic routes followed for halo-fluorophores (Fl–X).

Characterization data for halo-fluorophores and their precursors

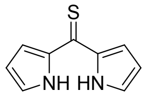

Data for 2,2′-Dipyrrilthione:[1] A dry round-bottomed flask, filled with argon gas and containing a Teflon magnetic stir bar, was equipped with a dropping funnel. A dry ether solution (5 mL) of pyrrole (1.16 g, 1.20 mL, 17.29 mmol), was added to this flask, which was then cooled to 0°C using an ice bath. Subsequently, a solution of thiophosgene (994 mg, 663 µL, 8.64 mmol, 0.5 equiv), dissolved in another 5 mL of dry ether, was placed into the dropping funnel and added dropwise to the stirring solution of pyrrole over a period of 5 minutes. [***Caution!*** *Thiophosgene is a highly toxic volatile chemical. Handling it requires a fume hood!*] After stirring the solution at this temperature for an additional 10 minutes, methanol (2.0 mL) was slowly introduced, and the solution was allowed to stir at this temperature for half an hour. Then, the solvent was removed under vacuum, and the residue was dissolved in minimal chloroform and passed through a short pad of silica gel. Following evaporation under vacuum, the black oily sample was purified over flash silica gel column chromatography using a toluene/chloroform (9:1) eluent, yielding the **2,2'-dipyrrylthione** as a dark purple solid (610 mg, 40%). ^{1}H NMR (400 MHz, Chloroform-*d*) δ 9.71 (s, 2H), 7.16 – 7.07 (m, 2H), 7.04 – 6.91 (m, 2H), 6.38 – 6.28 (m, 2H). ^{13}C{^{1}H} NMR (101 MHz, $CDCl_3$) δ 193.2, 138.4, 127.7, 114.8, 112.5. HRMS (ESI) m/z: $[M - H]^-$ Calcd for $C_9H_7N_2S$ 175.0330; Found 175.0341. IR (neat) 3326, 2973, 2880, 1380, 1087 cm^{-1}.

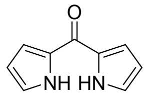

Data for 2,2′-Dipyrrilketone:[2] To a solution of **2,2'-dipyrrylthione** (0.8 g, 4.5 mmol, 1.0 equiv) and KOH (1.15 g, 20.4 mmol, 4.5 equiv) in 25 mL of aqueous methanol (95%, v/v) was added 3.3 mL of hydrogen peroxide (30% aqueous solution) dropwise at 0°C. The resultant solution was refluxed for 5 minutes using a heating mantle, and the solution was cooled using an ice bath. Subsequently, 40 mL of water was added to the solution, and it was cooled again to 0°C. Through this process, the product (**2,2'-dipyrrylketone**) precipitated as a pale yellow solid. The solid was then suction filtered using filter paper and a Büchner funnel. This solid was further purified by passing it through a short pad of silica gel using dichloromethane as the eluant to separate it from any unreacted starting material. The product was isolated as white amorphous solid (473 mg, 65%). ^{1}H NMR (400 MHz, Chloroform-*d*) δ 9.97 (s, 2H), 7.14 – 7.06 (m, 2H), 7.05 – 6.98 (m, 2H), 6.30 – 6.23 (m, 2H). ^{13}C{^{1}H} NMR (101 MHz, $CDCl_3$) δ 173.0, 130.5, 124.1, 116.1, 111.0. HRMS (ESI) m/z: $[M + H]^+$ Calcd for $C_9H_9N_2O$ 161.0715; Found 161.0704. IR (neat) 3323, 2973, 2881, 1413, 1380, 1325, 1087, 1045, 880 cm^{-1}.

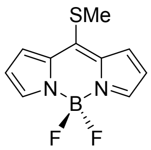

Data for 1a:[1] To a solution of **2,2'-dipyrrylthione** (0.30 g, 1.7 mmol) in dichloromethane (5 mL), methyl iodide (0.48 mL, 7.67 mmol, 4.5 equiv) was added at room temperature. After stirring for 24 hours, the solvent was removed under vacuum to obtain **methylthiopyrrolium iodide** as a dark brown gummy solid. This solid, without isolation and further purification, was dissolved in dichloromethane (7 mL) under an argon atmosphere at room temperature, and triethylamine (2.4 mL, 10 equiv) was added dropwise. After stirring for 30 minutes, $BF_3·OEt_2$ (2.1 mL, 10 equiv) was added dropwise, and the mixture was stirred for an additional 30 minutes. The solvent was then removed under vacuum, and the crude product was chromatographed on silica gel (eluant: 15% EtOAc in *n*-hexane), yielding **1a** as a dark red solid (0.22 g, 55%). ^{1}H NMR (400 MHz, Chloroform-*d*) δ 7.72 (s, 2H), 7.37 – 7.29 (m, 2H), 6.48 – 6.43 (m, 2H), 2.83 (d, *J* = 3.5 Hz, 3H). ^{13}C{^{1}H} NMR (101 MHz, $CDCl_3$) δ 152.3, 139.5, 132.0, 125.9, 116.1, 18.7. HRMS (ESI) m/z: $[M-H]^-$ Calcd for $C_{10}H_8BF_2N_2S$ 237.0469; Found 237.0484. IR (neat) 3125, 3103, 2920, 1520, 1467, 1411, 1384, 1366, 1347, 1312, 1265, 1229, 1180 cm^{-1}.

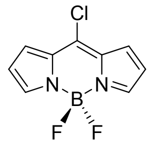

Data for 1b:[3] A stirring solution of **2,2'-dipyrrylketone** (320 mg, 2.0 mmol, 1 equiv) in 1,2-dichloroethane (10 mL) was slowly treated with phosphoryl chloride (0.37 mL, 4.0 mmol, 2 equiv) at room temperature. The resulting solution was then refluxed for 3 hours using a heating mantle. After refluxing, the solution was cooled using an ice bath, and triethylamine (2.8 mL, 20 mmol, 10 equiv) was added dropwise, maintaining the temperature at 0–4°C. After stirring for 15 minutes at this temperature, boron trifluoride etherate (2.7 mL, 22 mmol, 11 equiv) was added dropwise, while keeping the solution cold at 0–4°C. The solution was allowed to slowly warm to room temperature and was stirred for an additional 2 hours at this temperature. After this period, the solution was poured onto 60 mL of ether and extracted with water (2x50 mL). The resulting solution was then dried over anhydrous $MgSO_4$ powder, filtered (through filter paper), and the solvent was removed under vacuum. The crude product obtained was purified by silica gel column chromatography (eluant: dichloromethane:petroleum ether, 1:1). The product was obtained as a dark orange solid (268 mg, 59%). ^{1}H NMR (400 MHz, Chloroform-*d*) δ 7.81 (s, 2H), 7.36 – 7.26 (m, 2H), 6.56 – 6.45 (m, 2H). ^{13}C{^{1}H} NMR (101 MHz, $CDCl_3$) δ 145.1, 141.2, 134.0, 129.3, 119.1. HRMS (ESI) m/z: $[M\cdot]^-$ Calcd for $C_9H_6BClF_2N_2$ 226.0281; Found 226.0296. IR (neat) 3126, 3107, 2923, 1797, 1757, 1558, 1486, 1444, 1411, 1386, 1353, 1257 cm^{-1}.

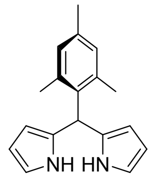

Data for 2,2'-(mesitylmethylene)bis(1*H*-pyrrole):[4] Pyrrole (3.02 g, 45 mmol, 3 equiv) was added to 100 mL of a 0.18 M aqueous solution of HCl (1.5:98.5, v/v), followed by the addition of mesitaldehyde (2.22 g, 15 mmol, 1 equiv). The resulting mixture was stirred overnight at room temperature.

The resulting semi-solid precipitate was filtered and washed with water and petroleum ether, yielding mesityldipyrromethane as a dark green solid. Further purification was achieved through silica gel column chromatography (eluant: dichloromethane:*n*-hexane, 1:1). The product was obtained as an off-white solid (1.0 g, 28%). ^{1}H NMR (400 MHz, Chloroform-*d*) δ 7.85 (s, 2H), 6.79 (s, 2H), 6.63 – 6.54 (m, 4H), 6.13 – 6.08 (m, 2H), 5.95 – 5.91 (m, 2H), 5.85 (s, 1H), 2.20 (s, 3H), 1.98 (s, 6H). ^{13}C{^{1}H} NMR (101 MHz, $CDCl_3$) δ 137.6, 136.6, 134.5, 131.3, 130.4, 116.2, 108.7, 106.5, 38.3, 20.8, 20.6. HRMS (ESI) m/z: $[M + H]^+$ Calcd for $C_{18}H_{21}N_2$ 265.1705; Found 265.1703. IR (neat) 3399, 3382, 2911, 1718, 1608, 1558, 1481, 1449, 1418, 1379, 1296, 1170, 1113, 1093, 1030 cm^{-1}.

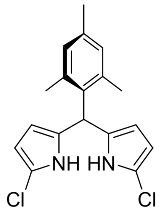

Data for 5,5'-(mesitylmethylene)bis(2-chloro-1*H*-pyrrole):[5] A THF (sodium/benzophenone-dried) solution (80 mL) of **2,2'-(mesitylmethylene)bis(1*H*-pyrrole)** (1.0 g, 3.78 mmol) was shielded from light by wrapping it in foil. The solution was then purged with Ar gas for 10 minutes and cooled down to –78 °C. Separately, *N*-chlorosuccinimide (1.01 g, 7.57 mmol, 2 equiv) was dissolved in THF (80 mL) and also protected from light and was introduced dropwise via a syringe over the course of 1 hour. The reaction mixture was stirred for an additional 2 hours at –78 °C, sealed, and stored in a freezer at –20 °C overnight. The resulting olive-green solution was concentrated, and the residue was diluted with dichloromethane (100 mL) and washed with water (2 × 20 mL). The organic phase was dried over Na_2SO_4. Column chromatography (using silica gel with a 1:1 mixture of *n*-hexanes and dichloromethane as eluant) gave the product as a greenish brown oil (695 mg, 55%). Due to low air/light stability of the product, we were not able to take NMR data. However, the spectra are given (see Figure 32S and 3S). HRMS (ESI) m/z: $[M + H]^+$ Calcd for $C_{18}H_{19}Cl_2N_2$ 333.0925; Found 333.0921. IR (neat) 3430, 3382, 2967, 2921, 1607, 1570, 1464, 1411, 1379, 1296, 1242, 1211, 1138, 1035 cm^{-1}.

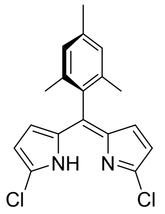

Data for (Z)-2-chloro-5-((5-chloro-2*H*-pyrrol-2-ylidene)(mesityl)methyl)-1*H*-pyrrole:[5] A dichloromethane (25 mL) solution of **5,5'-(mesitylmethylene)bis(2-chloro-1*H*-pyrrole)** (550 mg, 1.65 mmol) was purged with Ar for 5 minutes. Then, a suspension of DDQ (375 mg, 1.65 mmol) in dichloromethane (10 mL) was gradually added dropwise over a period of 5 minutes. The reaction mixture was stirred at room temperature for 1 hour, after which sat'd $NaHCO_3$ (20 mL) was introduced. The organic phase was washed with water (20 mL), dried over anhydrous Na_2SO_4, and concentrated to yield a red-brown solid. This crude was column chromatographed (silica gel, 4:1 mixture of hexanes and dichloromethane as eluant) to gave product as reddish-brown solid (385 mg, 70%). ^{1}H NMR (400 MHz, Chloroform-*d*) δ 6.83 (s, 2H), 6.25 (d, J = 4.2 Hz, 2H), 6.11 (d, J = 4.3 Hz, 2H), 2.26 (s, 3H), 1.99 (s, 6H). ^{13}C{^{1}H} NMR (101 MHz, $CDCl_3$) δ

141.3, 138.9, 138.4, 138.0, 136.9, 131.5, 128.6, 127.9, 117.1, 21.1, 19.8. HRMS (ESI) m/z: $[M + H]^+$ Calcd for $C_{18}H_{17}Cl_2N_2$ 331.0769; Found 331.0768. IR (neat) 2953, 2917, 2162, 1610, 1576, 1502, 1480, 1440, 1419, 1397, 1362, 1324, 1247, 1214, 1168, 1158, 1144, 1121, 1032 cm^{-1}.

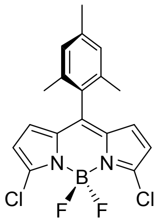

Data for 2a:[5] To a dichloromethane (30 mL) solution of **(*Z*)-2-chloro-5-((5-chloro-2*H*-pyrrol-2-ylidene)(mesityl)methyl)-1*H*-pyrrole** (250 mg, 0.76 mmol) was added triethylamine (1.0 mL, 7.6 mmol, 10 equiv) in one portion. The resulting mixture was stirred for 1 h at room temperature. BF_3•OEt_2 (1.86 mL, 30.2 mmol, 20 equiv) was added dropwise via a micropipette and the reaction was stirred in the dark for 12 h. Water (20 mL) was added and the layers were separated. The organic phase was dried over Na_2SO_4 and concentrated under vacuum. Purification by column chromatography (silica gel, 1:1 dichloromethane/hexanes) gave **2a** as an orange solid (258 mg, 90%). ^1H NMR (400 MHz, Chloroform-*d*) δ 6.88 (s, 2H), 6.53 (d, J = 4.2 Hz, 2H), 6.29 (d, J = 4.2 Hz, 2H), 2.28 (s, 3H), 2.02 (s, 6H). $^{13}C\{^1H\}$ NMR (101 MHz, $CDCl_3$) δ 144.9, 144.1, 139.2, 136.6, 134.1, 130.3, 128.3, 128.2, 119.0, 21.1, 19.9. HRMS (ESI) m/z: $[M + H]^+$ Calcd for $C_{18}H_{16}BCl_2F_2N_2$ 379.0752; Found 379.0749. IR (neat) 3163, 2916, 1786, 1609, 1538, 1468, 1458, 1389, 1374, 1328, 1312, 1258, 1190, 1164 cm^{-1}.

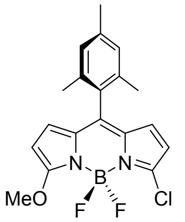

Data for 2b:[5] To a THF solution (10 mL) of **2a** (100 mg, 0.26 mmol) at 0 °C (ice-bath), sodium methoxide (54 μL, 0.29 mmol, 30 wt %) in methanol (15 mL) was added dropwise over 30 min. The reaction was stirred at 0 °C for 2 h. The resulting mixture was diluted with dichloromethane (50 mL) and washed with water (2 × 20 mL). The dichloromethane phase was separated, dried over Na2SO4 and concentrated under reduced pressure. The crude was chromatographed over silica gel (98:2 dichloromethane/ethyl acetate) to give **2b** as a bright orange solid (94 mg, 95%). ^1H NMR (400 MHz, Chloroform-*d*) δ 6.85 (s, 2H), 6.63 (d, J = 4.7 Hz, 1H), 6.25 (d, J = 4.0 Hz, 1H), 6.13 (d, J = 4.0 Hz, 1H), 6.03 (d, J = 4.7 Hz, 1H), 4.07 (s, 3H), 2.27 (s, 3H), 2.01 (s, 6H). $^{13}C\{^1H\}$ NMR (101 MHz, $CDCl_3$) δ 169.5, 139.9, 138.7, 137.0, 136.5, 133.9, 132.2, 130.5, 128.7, 128.2, 124.8, 115.5, 104.7, 59.3, 21.1, 19.9. HRMS (ESI) m/z: $[M + H]^+$ Calcd for $C_{19}H_{19}BClF_2N_2O$ 375.1247; Found 375.1244. IR (neat) 2918, 1582, 1551, 1520, 1476, 1450, 1408, 1387, 1353, 1326, 1284, 1235, 1188, 1167, 1109, 1051 cm^{-1}.

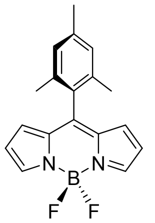

Data for 8-mesityl-BODIPY:[4] A dichloromethane (15 mL) solution of **5,5'-(mesitylmethylene)bis(2-chloro-1*H*-pyrrole)** (400 mg, 1.51 mmol) was purged with Ar for 5 minutes. Subsequently, a suspension of *p*-chloranil (410 mg, 1.66 mmol, 1.1 equiv) in dichloromethane (10 mL) was slowly added dropwise over a period of 5 minutes. After stirring at room temperature for 1 hour, triethylamine (2.0 mL, 15.2 mmol, 10 equiv) was added dropwise to the reaction mixture. The resulting mixture was then stirred for an additional hour at room temperature. BF_3•OEt_2 (3.7 mL, 60.4 mmol, 20 equiv) was

added dropwise via a micropipette and the mixture was stirred overnight. Water (20 mL) was added and the layers were separated. The organic phase was dried over Na_2SO_4 and concentrated under vacuum. Purification by column chromatography (silica gel, 1:1 dichloromethane/*n*-hexanes) gave **8-mesityl-BODIPY** as a bright orange solid (135 mg, 34%). ^1H NMR (400 MHz, Chloroform-*d*) δ 7.84 (s, 2H), 6.88 (s, 2H), 6.60 (d, J = 4.2 Hz, 2H), 6.39 (d, J = 3.8 Hz, 3H), 2.29 (s, 3H), 2.02 (s, 6H). $^{13}C\{^1H\}$ NMR (101 MHz, $CDCl_3$) δ 147.7, 144.3, 138.9, 136.3, 135.4, 130.2, 129.7, 128.2, 118.6, 21.2, 20.0. HRMS (ESI) m/z: [M ·]$^-$ Calcd for $C_{18}H_{17}BF_2N_2$ 310.1453; Found 310.1458. IR (neat) 1610, 1542, 1480, 1449, 1407, 1384, 1355, 1257, 1219, 1168, 1138, 1106, 1039 cm^{-1}.

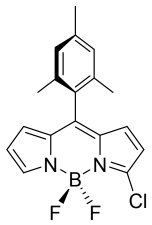

Data for 2c:[6] A mixture of **8-mesityl-BODIPY** (62 mg, 0.2 mmol) and $CuCl_2$•$2H_2O$ (102 mg, 0.6 mmol) in CH_3CN (5 mL) was refluxed in an open air atmosphere for 2 hours using a heating mantle. After cooling to room temperature, the reaction mixture was transferred to dichloromethane (30 mL), washed with water (3 × 50 mL), dried over anhydrous Na_2SO_4, filtered, and concentrated under vacuum. The resulting residue was subjected to purification via column chromatography on silica gel, using petroleum ether/ethyl acetate (40:1, v/v) as eluant. The product **2c** was obtained as a bright orange solid (36 mg, 52%). ^1H NMR (400 MHz, Chloroform-*d*) δ 7.84 (s, 1H), 6.88 (s, 2H), 6.60 (d, J = 4.2 Hz, 1H), 6.56 (d, J = 4.3 Hz, 1H), 6.42 (dd, J = 4.4, 1.8 Hz, 1H), 6.28 (d, J = 4.3 Hz, 1H), 2.29 (s, 3H), 2.02 (s, 6H). $^{13}C\{^1H\}$ NMR (101 MHz, $CDCl_3$) δ 144.9, 143.6, 143.3, 138.0, 135.4, 134.1, 133.3, 129.4, 129.1, 127.9, 127.2, 118.0, 117.4, 20.1, 18.9. HRMS (ESI) m/z: [M ·]$^-$ Calcd for $C_{18}H_{16}BClF_2N_2$ 344.1063; Found 344.1053. IR (neat) 3128, 2921, 1609, 1547, 1485, 1454, 1402, 1384, 1342, 1319, 1259 cm^{-1}.

General procedure for the synthesis of BODIPY/NBD-Tetrazines

To a 25 mg packaged commercial tetrazine (**TzH**: 112 μmol or **TzMe**: 105 μmol) vial containing a Teflon-coated magnetic stir bar, 1.1 equivalents of (*pseudo*)halo-BODIPYs (**1a** or **1b** or **2a**) or 4-fluoro-7-nitrobenzofurazan (**3b**) compound were added. To this, 2 mL DCM was added and the suspension was left to stir for 30 seconds. Then, 1–2 equivalents of triethyl amine were added at once and the solution was left to stir vigorously at room temperature. The progress of the reaction was monitored by TLC (eluant: DCM:EtOAc 20:1 or 50:1); and the reaction mixture was directly loaded onto a flash silica gel column when the reaction was complete. Caution: The reactions should not be stirred for longer than the specified times (Table 1 of the manuscript). Otherwise, the product starts to precipitate necessitating much more solvent to redissolve the precipitated solids (particularly valid for **1TzH**/**Me** and **3TzH**/**Me**), which deteriorates separation efficiency. The product was double chromatographed to ensure spectroscopically pure dyes. It should be also noted why we exclusively employ DCM as the

reaction solvent (and as the column eluant!): its low boiling point (39.6 °C) further minimizes the risk of thermal degradation of the products as DCM can readily be removed.

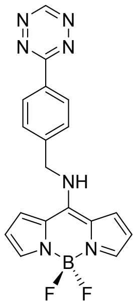

Data for 1TzH: The reaction of **1a** (employing 1 equiv of TEA and stirring for 1h) gave the product as a maroon solid (24.8 mg, 59% yield). The reaction of **1b** (employing 2 equiv of TEA and stirring for 30 min) gave the same product (26.5 mg, 63% yield). Eluant: 20:1 DCM:EtOAc. ^{1}H NMR (400 MHz, DMSO-d_6) δ 10.60 (s, 1H), 10.41 (bs, 1H), 8.54 (d, J = 8.4 Hz, 2H), 7.72 (d, J = 8.3 Hz, 2H), 7.62 (d, J = 3.9 Hz, 1H), 7.57 (bs, 1H), 7.44 (bs, 1H), 7.11 (d, J = 4.1 Hz, 1H), 6.46 (ddd, J = 8.0, 4.0, 2.2 Hz, 2H), 5.21 (d, J = 5.0 Hz, 2H). ^{13}C{^{1}H} NMR (101 MHz, DMSO-d_6) δ 165.3, 158.1, 148.9, 140.9, 134.0, 131.25, 131.21, 128.4, 127.6, 125.1, 123.7, 121.5, 117.2, 114.5, 113.3, 54.9, 48.9. HRMS (ESI) m/z: $[M + Na]^+$ Calcd for $C_{18}H_{14}BF_2N_7Na$ 400.1264; Found 400.1253. IR (neat) 3362, 3100, 1590, 1554, 1513, 1463, 1438, 1425, 1387, 1370, 1338, 1302, 1277, 1229 cm^{-1}. UV-vis: λ_{abs} 402 nm (MeCN).

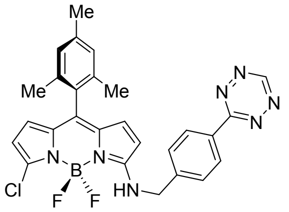

Data for 2TzH: The reaction of **2a** (employing 2 equiv of TEA and stirring for 1h) gave the product as a dark orange-brown solid (37.6 mg, 64% yield). Eluant: 50:1 DCM:EtOAc. ^{1}H NMR (400 MHz, $CDCl_3$) δ (1:2 rotameric relationship was observed. See variable temperature ^{1}H-NMR spectra provided below in Figure 2S.) 10.17 (s, 1H), 8.58 (d, J = 8.4 Hz, 2H), 7.54 (d, J = 2.5 Hz, 1H), 7.52 (d, J = 2.5 Hz, 1H), 6.87 – 6.80 (m [overlapping two singlets peaking at 6.84 and 6.83], 2H), 6.74 (bs, 0.66H), 6.69 (bs, 0.34H), 6.61 (d, J = 4.9 Hz, 0.66H), 6.58 (d, J = 4.9 Hz, 0.34H), 6.17 (d, J = 2.6 Hz, 0.34H), 6.06 (d, J = 3.9 Hz, 0.66H), 6.05 – 6.00 (m, 1H), 4.73 – 4.61 (m, 2H), 2.25 (s, 3H), 2.04 – 1.99 (m [overlapping two singlets peaking at 2.02 and 2.01], 6H). ^{13}C{^{1}H} NMR (101 MHz, $CDCl_3$) δ (rotameric relationship was observed) 165.1, 160.8, 160.6, 156.9, 140.89, 140.87, 137.2, 137.1, 136.3, 136.0, 134.7, 133.8, 133.2, 132.4, 131.3, 130.8, 130.6, 129.5, 129.2, 128.3, 128.1, 127.1, 126.94, 126.85, 126.76, 124.9, 122.0, 119.1, 113.4, 112.1, 109.34, 109.25, 76.3, 76.2, 76.0, 75.7, 52.4, 46.98, 46.95, 28.7, 20.14, 20.07, 18.9. HRMS (ESI) m/z: $[M + Na]^+$ Calcd for $C_{27}H_{23}BClF_2N_7Na$ 552.1657; Found 552.1632. IR (neat) 3385, 3101, 2918, 1605, 1576, 1522, 1510, 1490, 1436, 1402, 1375, 1350, 1299, 1259, 1236 cm^{-1}. UV-vis: λ_{abs} 472 nm (MeCN).

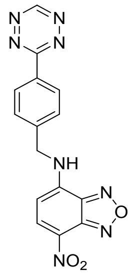

Data for 3TzH: The reaction of **3b** (employing 2 equiv of TEA and stirring for 15min) gave the product as an orange solid (16 mg, 41% yield). Eluant: 20:1 DCM:EtOAc. ^{1}H NMR (400 MHz, DMSO-d_6) δ 10.46 (s, 1H), 9.91 (s, 1H), 8.37 (d, J = 2.6 Hz, 2H), 8.35 (d, J = 1.9 Hz, 2H), 7.59 (s, 1H), 7.55 (s, 1H), 6.26 (s, 1H), 4.76 (s, 2H). ^{13}C{^{1}H} NMR (101 MHz, DMSO-d_6) δ 165.7, 158.5, 145.2, 144.9, 144.5, 142.5, 138.1, 131.4, 128.5, 128.4, 122.1, 100.2, 46.4. HRMS (ESI) m/z: $[M + Na]^+$ Calcd for $C_{15}H_{10}N_8O_3Na$ 373.0774; Found 373.0768. UV-vis: λ_{abs} 454 nm (MeCN).

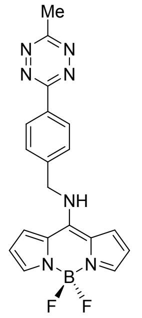

Data for 1TzMe: The reaction of **1a** (employing 1 equiv of TEA and stirring for 1h) gave the product as a shiny maroon solid (35 mg, 85% yield). The reaction of **1b** (employing 2 equiv of TEA and stirring for 30min) gave the same product (32 mg, 77% yield). Eluant: 20:1 DCM:EtOAc. ^{1}H NMR (400 MHz, DMSO-d_6) δ 10.39 (s, 1H), 8.51 (d, J = 8.3 Hz, 2H), 7.70 (d, J = 8.3 Hz, 2H), 7.62 (s, 1H), 7.57 (s, 1H), 7.44 (s, 1H), 7.12 (d, J = 3.1 Hz, 1H), 6.53 – 6.40 (m, 2H), 5.20 (s, 2H), 2.99 (s, 3H). ^{13}C{^{1}H} NMR (101 MHz, DMSO-d_6) δ 167.5, 163.4, 149.2, 140.8, 134.4, 131.6, 128.4, 127.9, 125.5, 124.1, 121.8, 117.5, 114.9, 113.7, 49.4, 21.2 (One aromatic carbon was not located.). HRMS (APCI) m/z: $[M - H]^-$ Calcd for $C_{19}H_{15}BF_2N_7$ 390.1450; Found 390.1451. UV-vis: λ_{abs} 402 nm (MeCN).

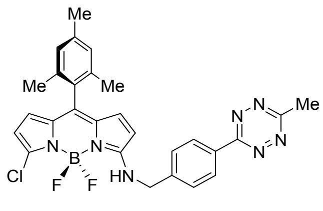

Data for 2TzMe: The reaction of **2a** (employing 2 equiv of TEA and stirring for 1h) gave the product as a dark orange-brown solid (52 mg, 91% yield). Eluant: 50:1 DCM:EtOAc. ^{1}H NMR (400 MHz, DMSO-d_6) δ 9.08 (s, 1H), 8.47 (d, J = 8.3 Hz, 2H), 7.68 (d, J = 8.3 Hz, 2H), 6.97 (s, 2H), 6.69 (s, 2H), 6.16 (d, J = 3.6 Hz, 1H), 5.84 (d, J = 3.6 Hz, 1H), 4.80 (d, J = 5.4 Hz, 2H), 3.00 (s, 3H), 2.29 (s, 3H), 2.00 (s, 6H). ^{13}C{^{1}H} NMR (101 MHz, DMSO-d_6) δ 167.5, 163.5, 162.5, 143.6, 137.9, 136.9, 135.2, 134.4, 131.4, 129.6, 128.5, 128.4, 128.1, 127.2, 126.0, 116.4, 115.0, 112.2, 47.3, 21.2, 21.0, 19.8 (One aromatic carbon was not located.). HRMS (APCI) m/z: $[M - H]^-$ Calcd for $C_{28}H_{24}BClF_2N_7$ 542.1843; Found 542.1847. UV-vis: λ_{abs} 474 nm (MeCN).

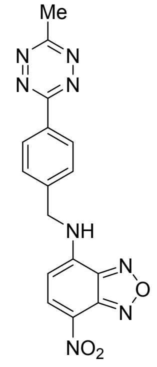

Data for 3TzMe: The reaction of **3b** (employing 2 equiv of TEA and stirring for 15min) gave the product as an orange solid (29 mg, 75% yield). Eluant: 20:1 DCM:EtOAc. ^{1}H NMR (400 MHz, DMSO-d_6) δ 10.04 (s, 1H), 8.49 (d, *J* = 8.9 Hz, 1H), 8.45 (d, *J* = 8.3 Hz, 2H), 7.69 (d, *J* = 8.3 Hz, 2H), 6.38 (d, *J* = 7.7 Hz, 1H), 4.88 (s, 2H), 2.99 (s, 3H). ^{13}C{^{1}H} NMR (101 MHz, DMSO-d_6) δ 167.5, 163.4, 145.3, 144.9, 144.5, 142.0, 138.1, 131.4, 128.5, 128.1, 122.0, 100.2, 46.4, 21.2. HRMS (APCI) m/z: $[M-H]^-$ Calcd for $C_{16}H_{11}N_8O_3$ 363.0954; Found 363.0948. UV-vis: λ_{abs} 455 nm (MeCN).

General procedure for inverse electron demand Diels–Alder reactions:

To a vigorously stirred dichloromethane solutions (20 mL) of Fl-Tz dyes, **BCN** (1.5 equiv) was added as solid at once at room temperature. The reactions of H-terminated Fl–Tz dyes were completed in 5 min, and those of Me-terminated in 30 min. The solvent was removed under reduced pressure; and the crude product was subjected to FCC. The 90:10 DCM:MeOH eluant system was used to purify all of the cycloaddition products.

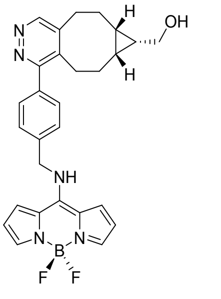

Data for 1TzH•BCN: A 5 min. reaction of **1TzH** (15.0 mg, 40 μmol) and **BCN** (9.0 mg, 60 μmol) in DCM (20 mL) gave the adduct as a pale-yellow solid (18.3 mg, 92% yield). ^{1}H NMR (400 MHz, DMSO-d_6) δ 10.39 (s, 1H), 8.98 (s, 1H), 7.75 – 7.48 (m, 6H), 7.43 (s, 1H), 7.20 (s, 1H), 6.47 (d, *J* = 12.3 Hz, 2H), 5.16 (d, *J* = 5.8 Hz, 2H), 3.13 – 2.96 (m, 1H), 2.93 – 2.72 (m, 2H), 2.65 – 2.53 (m, 1H), 2.26 – 2.09 (m, 1H), 2.04 – 1.84 (m, 1H), 1.58 (s, 2H), 1.23 (s, 1H), 1.05 (t, *J* = 7.0 Hz, 1H), 1.00 – 0.88 (m, 1H), 0.78 (s, 2H) (One proton was not located.). ^{13}C{^{1}H} NMR (101 MHz, DMSO-d_6) δ 161.3, 152.1, 149.2, 142.7, 140.1, 137.3, 136.5, 134.3, 131.5, 129.9, 126.9, 125.5, 124.1, 121.9, 117.5, 114.8, 113.6, 57.4, 56.4, 49.5, 30.0, 27.4, 23.5, 22.3, 18.9, 18.6. HRMS (ESI) m/z: $[M+Na]^+$ Calcd for $C_{28}H_{28}BF_2N_5ONa$ 522.2253; Found 522.2293. UV-vis: λ_{abs} 401 nm (MeCN).

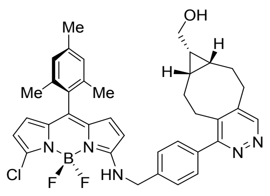

Data for 2TzH•BCN: A 5 min. reaction of **2TzH** (25.5 mg, 48 μmol) and **BCN** (11.0 mg, 72 μmol) in DCM (10 mL) gave the adduct as an orange solid (31.7 mg, 95% yield). ^{1}H NMR (400 MHz, DMSO-d_6) δ 9.06 (s, 1H), 8.95 (s, 1H), 7.60 – 7.41 (m, 4H), 6.98 (s, 2H), 6.80 – 6.62 (m, 2H), 6.16 (d, *J* = 3.7 Hz, 1H), 5.83 (d, *J* = 3.7 Hz, 1H), 5.78 – 5.72 (m, 1H), 4.77 (d, *J* = 5.8 Hz, 2H), 4.28 (s, 1H),

3.48 (s, 2H), 3.09 – 2.94 (m, 1H), 2.90 – 2.74 (m, 2H), 2.61 – 2.53 (m, 1H), 2.29 (s, 3H), 2.24 – 2.09 (m, 1H), 2.01 (s, 6H), 1.98 – 1.89 (m, 1H), 1.58 (s, 2H), 1.23 (s, 1H), 0.92 (dd, J = 15.8, 7.6 Hz, 1H), 0.78 (s, 2H). $^{13}C\{^1H\}$ NMR (101 MHz, DMSO-d_6) δ 162.6, 161.4, 152.2, 142.2, 139.6, 139.2, 137.9, 137.4, 136.9, 136.8, 135.2, 134.5, 131.4, 129.6, 128.4, 127.3, 127.0, 125.8, 116.3, 115.1, 112.1, 57.4, 55.2, 47.4, 29.9, 27.4, 23.5, 22.2, 21.0, 19.9, 18.9, 18.6. HRMS (APCI) m/z: $[M\cdot]^-$ Calcd for $C_{37}H_{37}BClF_2N_5O$ 651.2748; Found 651.2730. UV-vis: λ_{abs} 473 nm (MeCN).

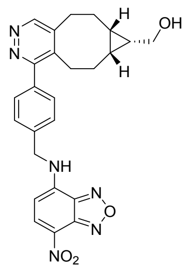

Data for 3TzH•BCN: A 5 min. reaction of **3TzH** (21.0 mg, 60 μmol) and **BCN** (13.5 mg, 90 μmol) in DCM (40 mL) gave the adduct as an orange solid (25.5 mg, 90% yield). ^1H NMR (400 MHz, DMSO-d_6) δ 10.04 (s, 1H), 9.06 – 8.86 (m, 1H), 8.62 – 8.38 (m, 1H), 7.65 – 7.52 (m, 2H), 7.52 – 7.40 (m, 2H), 6.45 (s, 1H), 5.82 – 5.69 (m, 1H), 4.84 (s, 2H), 3.12 – 2.93 (m, 1H), 2.93 – 2.73 (m, 2H), 2.28 – 2.07 (m, 1H), 2.02 – 1.82 (m, 1H), 1.57 (s, 2H), 1.35 – 1.03 (m, 1H), 1.02 – 0.86 (m, 1H), 0.77 (s, 2H) (Two proton were not located.). $^{13}C\{^1H\}$ NMR (101 MHz, DMSO-d_6) δ 161.4, 152.1, 145.3, 144.9, 144.5, 142.4, 139.9, 138.1, 137.6, 137.3, 129.6, 127.4, 121.8, 100.1, 57.4, 55.2, 46.4, 29.9, 27.4, 23.5, 22.2, 18.9, 18.6. HRMS (ESI) m/z: $[M + Na]^+$ Calcd for $C_{25}H_{24}N_6O_4Na$ 495.1757; Found 495.1770. UV-vis: λ_{abs} 456 nm (MeCN).

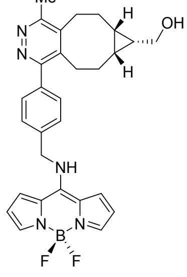

Data for 1TzMe•BCN: A 30 min. reaction of **1TzMe** (22.0 mg, 56 μmol) and **BCN** (12.7 mg, 84 μmol) in DCM (20 mL) gave the adduct as a pale-yellow solid (20.4 mg, 71% yield). ^1H NMR (400 MHz, DMSO-d_6) δ 10.35 (s, 1H), 7.66 – 7.38 (m, 7H), 7.21 (s, 1H), 6.47 (dd, J = 16.6, 1.9 Hz, 2H), 5.15 (s, 2H), 4.29 (s, 1H), 3.48 (s, 2H), 2.97 (dd, J = 13.9, 6.9 Hz, 1H), 2.91 – 2.73 (m, 2H), 2.68 (s, 3H), 2.36 – 2.17 (m, 1H), 1.93 (s, 1H), 1.57 (s, 2H), 1.40 – 1.19 (m, 1H), 0.98 – 0.80 (m, 1H), 0.69 (s, 2H). $^{13}C\{^1H\}$ NMR (101 MHz, DMSO-d_6) δ 160.4, 157.7, 149.2, 140.8, 139.0, 138.0, 136.1, 134.3, 131.4, 130.0, 127.0, 125.5, 124.1, 121.9, 117.4, 114.8, 113.6, 57.3, 55.2, 49.5, 27.7, 26.7, 23.6, 22.7, 22.1, 20.5, 18.5. HRMS (APCI) m/z: $[M - H]^-$ Calcd for $C_{29}H_{29}BF_2N_5O$ 512.2433; Found 512.2449. UV-vis: λ_{abs} 401 nm (MeCN).

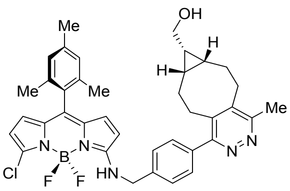

Data for 2TzMe•BCN: A 30 min. reaction of **2TzMe** (20.0 mg, 37 μmol) and **BCN** (8.3 mg, 55 μmol) in DCM (15 mL) gave the adduct as an orange solid (23.0 mg, 94% yield). ^{1}H NMR (400 MHz, DMSO-d_6) δ 9.06 (s, 1H), 7.52 (d, *J* = 7.9 Hz, 2H), 7.44 (d, *J* = 8.0 Hz, 2H), 6.98 (s, 2H), 6.71 (dd, *J* = 13.9, 5.0 Hz, 2H), 6.16 (d, *J* = 3.7 Hz, 1H), 5.83 (d, *J* = 3.7 Hz, 1H), 4.76 (d, *J* = 6.0 Hz, 2H), 4.28 (t, *J* = 5.0 Hz, 1H), 3.54 – 3.41 (m, 2H), 3.06 – 2.91 (m, 1H), 2.91 – 2.72 (m, 2H), 2.67 (s, 3H), 2.63 (m, 1H), 2.29 (s, 3H), 2.27 – 2.18 (m, 1H), 2.01 (s, 6H), 1.98 – 1.87 (m, 1H), 1.58 (s, 2H), 0.97 – 0.83 (m, 1H), 0.69 (s, 2H). ^{13}C{^{1}H} NMR (101 MHz, DMSO-d_6) δ 162.2, 160.1, 157.3, 140.4, 138.6, 137.5, 136.6, 134.8, 134.1, 131.0, 129.4, 129.2, 128.0, 126.9, 126.6, 125.5, 115.9, 114.8, 111.7, 79.1, 59.7, 57.0, 54.9, 47.0, 27.4, 26.3, 23.1, 21.8, 21.2, 20.7, 20.1, 19.5, 18.2. HRMS (APCI) m/z: $[M-H]^-$ Calcd for $C_{38}H_{38}BClF_2N_5O$ 664.2826; Found 664.2830. UV-vis: λ_{abs} 473 nm (MeCN).

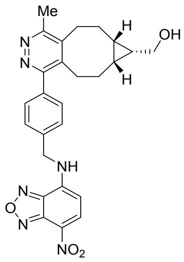

Data for 3TzMe•BCN: A 30 min. reaction of **3TzMe** (29.0 mg, 80 μmol) and **BCN** (12.0 mg, 120 μmol) in DCM (20 mL) gave the adduct as an orange solid (33.0 mg, 85% yield). ^{1}H NMR (400 MHz, DMSO-d_6) δ 10.05 (s, 1H), 8.51 (d, *J* = 8.9 Hz, 1H), 7.55 (d, *J* = 8.0 Hz, 2H), 7.42 (d, *J* = 8.0 Hz, 2H), 6.45 (d, *J* = 8.4 Hz, 1H), 4.83 (s, 2H), 4.29 (t, *J* = 5.0 Hz, 1H), 3.47 (t, *J* = 6.4 Hz, 2H), 3.05 – 2.90 (m, 1H), 2.90 – 2.72 (m, 2H), 2.66 (s, 3H), 2.62 (d, *J* = 6.6 Hz, 1H), 2.34 – 2.18 (m, 1H), 2.01 – 1.84 (m, 1H), 1.57 (s, 2H), 0.97 – 0.80 (m, 1H), 0.68 (s, 2H). ^{13}C{^{1}H} NMR (101 MHz, DMSO-d_6) δ 160.5, 157.7, 145.3, 144.9, 144.5, 140.7, 139.0, 138.2, 137.9, 137.2, 129.7, 127.4, 121.8, 100.1, 57.3, 55.2, 46.5, 27.7, 26.7, 23.4, 22.1, 21.7, 20.4, 18.6. HRMS (APCI) m/z: $[M-H]^-$ Calcd for $C_{26}H_{25}N_6O_4$ 485.1937; Found 485.1917. UV-vis: λ_{abs} 457 nm (MeCN).

Variable temperature ^{1}H-NMR spectra of 2TzH in $CDCl_3$

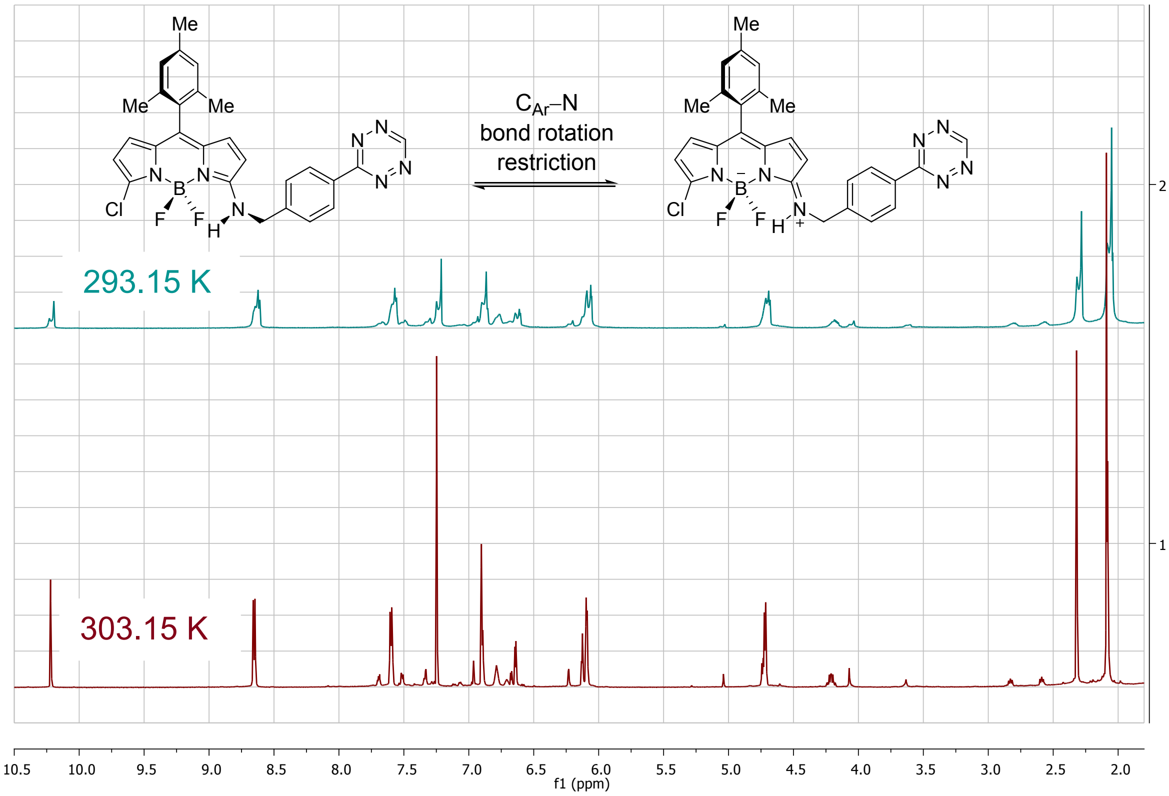

Figure 2S. Variable temperature ^{1}H NMR spectra (600 MHz) of **2TzH** in $CDCl_3$ (293.15 K vs. 303.15 K)

Solvatochromism of Fl–Tz or Fl–Tz•BCN dyes

Stokes shifts of Fl–Tz•BCN dyes in acetonitrile

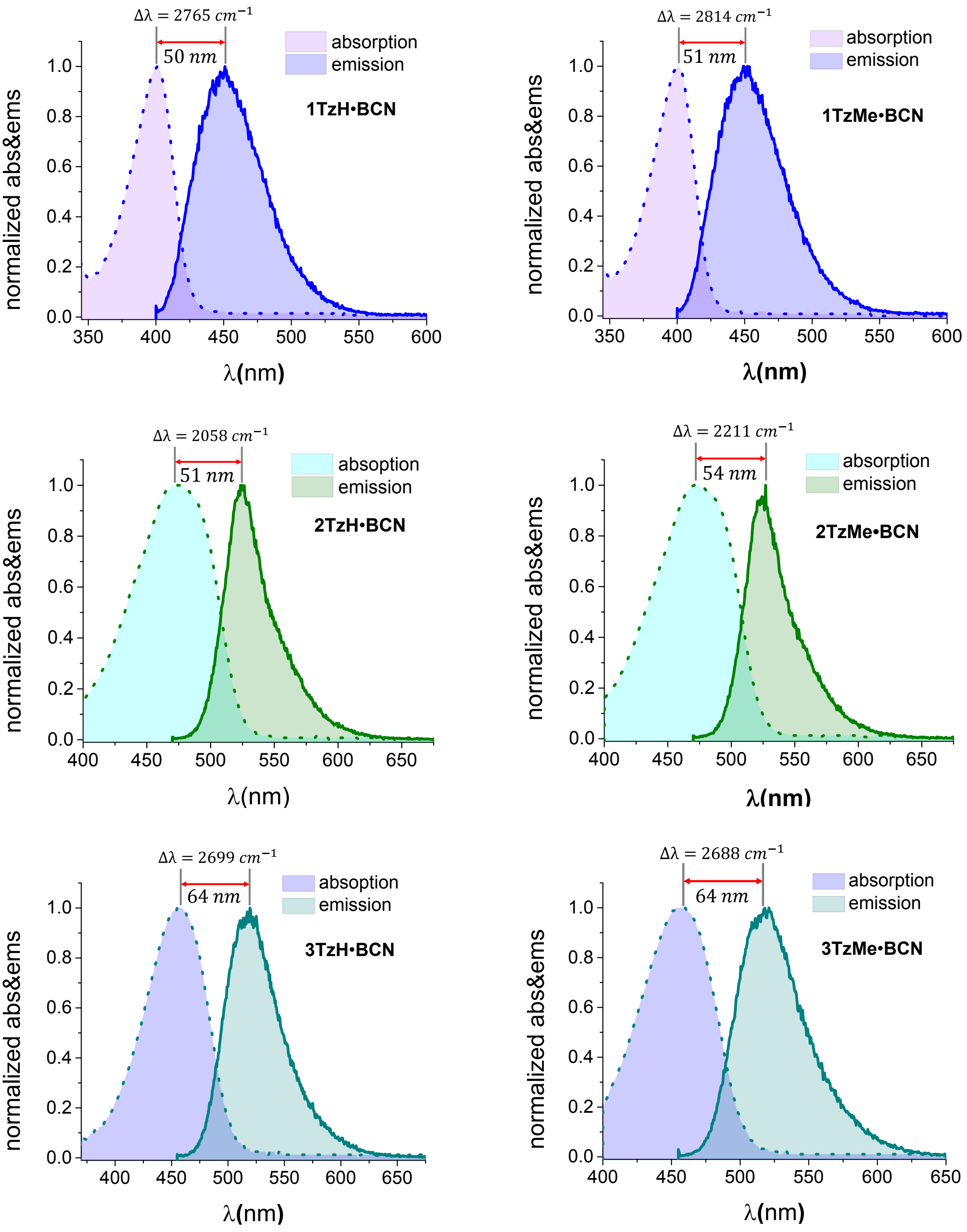

Figure 3S. Normalized absorption and emission spectra of Fl–Tz•BCN dyes in acetonitrile at room temperature. Δλ denotes Stokes shifts calculated in wavenumbers (cm^{-1}) or nanometers (nm).

Solvent screening studies of Fl–Tz and Fl–Tz•BCN dyes

We only considered the Me-ended Tzs and their BCN cycloadducts for this investigation due to their higher chemical stability and nearly identical spectral properties to those of H-ended Tzs. UV-vis electronic absorption spectra of Me-ended Fl–Tz and Fl–Tz•BCN dyes were acquired in organic solvents of varied polarities[10] (dimethylsulfoxide (DMSO), acetonitrile (MeCN), methanol (MeOH), acetone, dichloromethane, chloroform, toluene) each at a concentration of 5 μM. Fluorescence emission spectra were obtained from diluted solutions; refer to Table S1 footnotes for details.

Table 1S. Absorption and emission maxima of Fl–Tz and Fl–Tz•BCN dyes[a,b]

Fl–Tz or Fl–Tz•BCN	solvent	λ_{abs} [nm]	λ_{em} [nm]	$\Delta\lambda$ [nm]
**1TzMe**	DMSO	402	445	43
	MeCN	402	451	49
	MeOH	404	453	49
	Acetone	404	456	52
	Dichloromethane	414	468	54
	Chloroform	416	464	48
	Toluene	420	471	51
**2TzMe**	DMSO	471	535	64
	MeCN	474	530	56
	MeOH	475	527	52
	Acetone	474	527	53
	Dichloromethane	483	535	52
	Chloroform	485	534	49
	Toluene	486	534	48
**3TzMe**	DMSO	464	534	70
	MeCN	455	519	64
	MeOH	458	526	68
	Acetone	454	520	76

	Dichloromethane	445	507	62
	Chloroform	444	512	68
	Toluene	438	512	74
**1TzMe•BCN**	DMSO	402	455	53
	MeCN	401	452	55
	MeOH	403	454	51
	Acetone	402	459	57
	Dichloromethane	411	467	56
	Chloroform	413	468	55
	Toluene	415	473	58
**2TzMe•BCN**[c]	DMSO	471	526	55
	MeCN	473	527	51
	MeOH	473	520	47
	Acetone	473	522	49
	Dichloromethane	502	527	25
	Chloroform	509	528	19
	Toluene	511	527	16
**3TzMe•BCN**	DMSO	462	530	68
	MeCN	457	521	61
	MeOH	461	526	65
	Acetone	457	521	64
	Dichloromethane	447	512	65
	Chloroform	446	514	68
	Toluene	437	508	71

[a]A 5 µM solution of each dye was used for UV-vis electronic absorption studies of all the compounds. [b]In all fluorescence emission measurements, slit widths were kept constant at d_{ex}:5.0, d_{em}:2.5. For fluorescence measurements, Fl-Tz dyes were studied at 5 µM concentrations, whereas Fl-Tz•BCN dyes were studied at 1 µM concentrations unless otherwise noted. [c]The fluorescence emissions of the adduct 2TzMe•BCN were studied at 0.5 µM in dichloromethane and 0.125 µM concentrations, in chloroform and toluene.

Solvent screening spectra of Me-ended Fl–Tz and Fl–Tz•BCN dyes

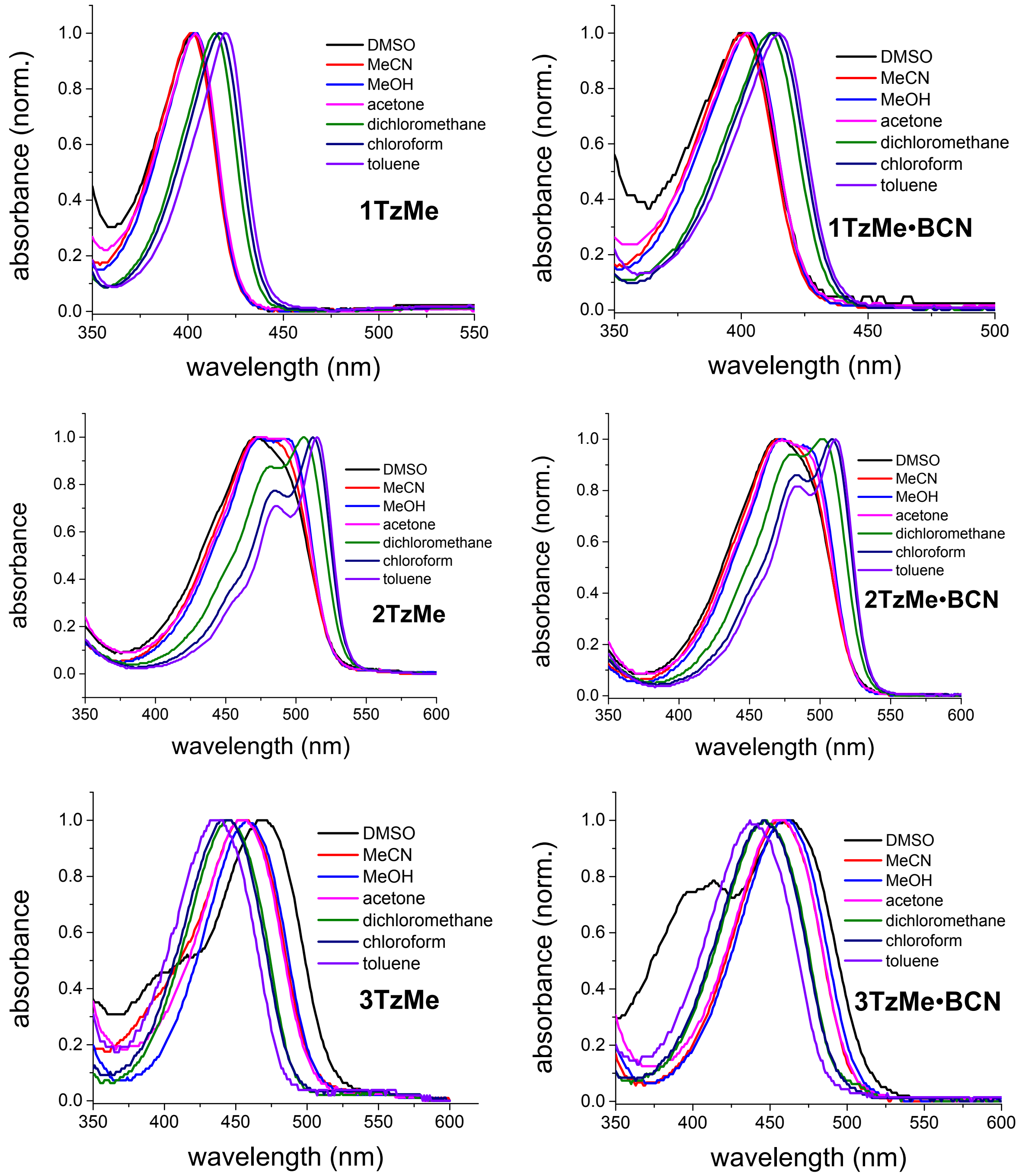

Figure 4S. Absorption spectra of Me-ended Fl–Tz and Fl–Tz•BCN dyes (5 µM each) in organic solvents.

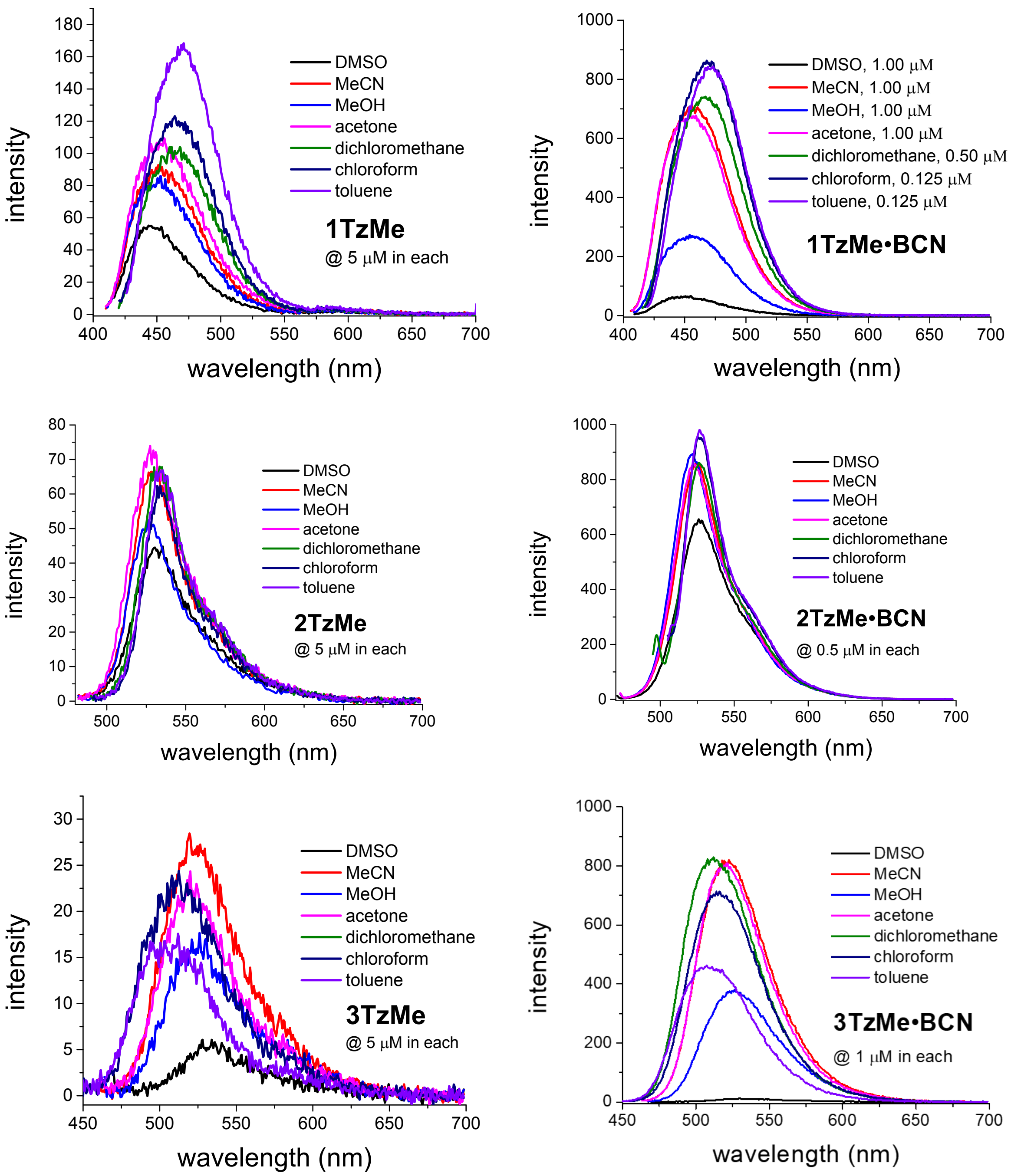

Figure 5S. Fluorescence emission spectra of Me-ended Fl–Tz and Fl–Tz•BCN dyes in organic solvents.

Fluorescence decay profiles of Fl–Tz and Fl–Tz•BCN dyes

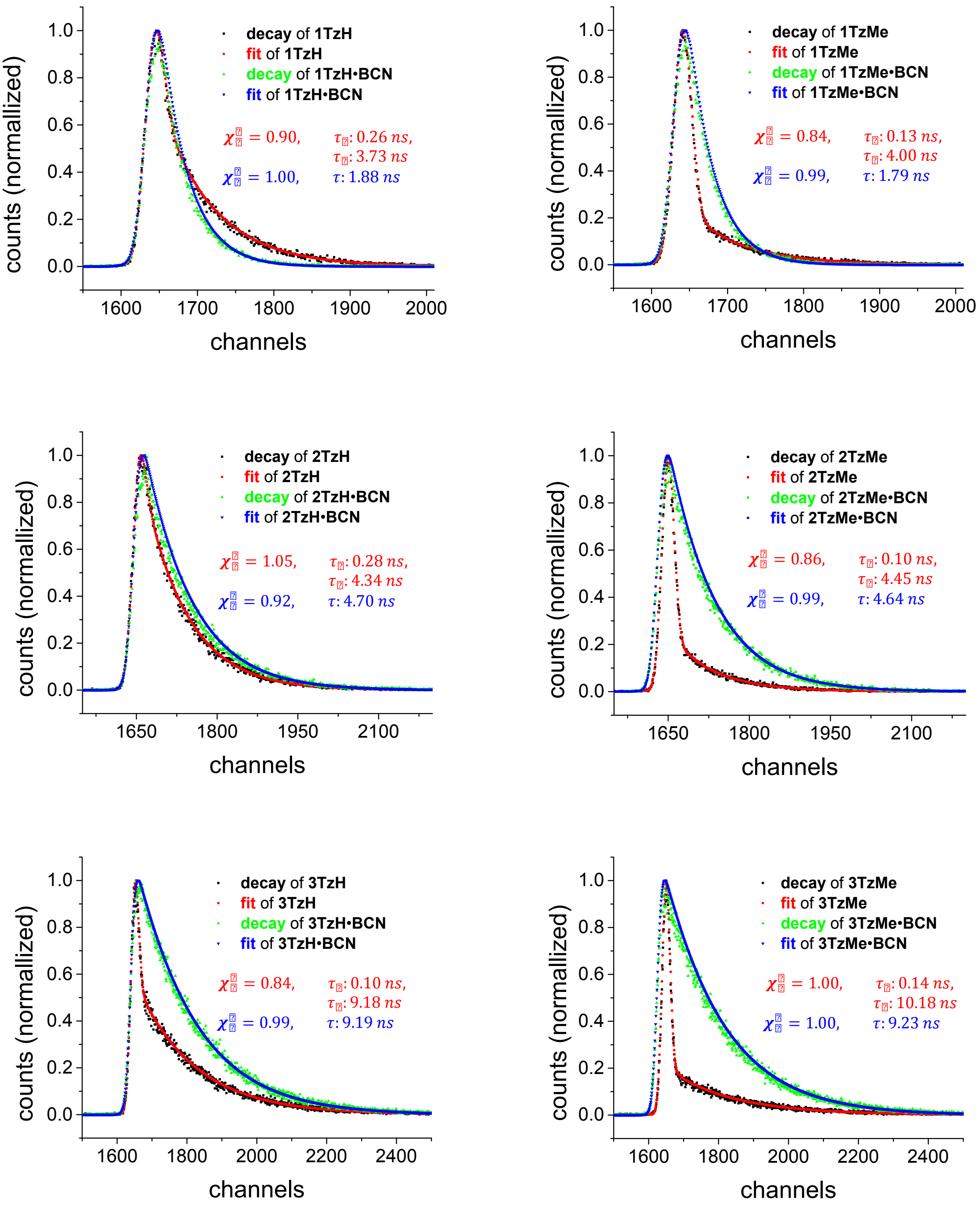

Figure 6S. Fluorescence emission decay profiles of Fl–Tz and Fl–Tz•BCN dyes (each at a concentration of 10 μM) were acquired in acetonitrile at room temperature. A 390 nm NanoLED source was used to excite the blue-emissive dyes, while a 450 nm NanoLED source was employed for exciting the green-emissive dyes. For all decay measurements, time calibration was set to 5.486969×10^{-11} sec/ch. The instrument response function was determined by employing dilute solutions of LUDOX® AS-40 colloidal silica (40 wt. % suspension in water). This response function was then corrected using DAS-6 v6.6 Horiba Jobin Yvon Fluorescence Decay Analysis Software to fit decay data of our Tz dyes and their BCN adducts.

pH Dependency of Fl–Tz and Fl–Tz•BCN dyes

In all pH dependency experiments, we used phosphate-buffered saline (PBS, 200 mM) for the pH range of 6.0–8.0 and acetate buffer (200 mM) to set the pH in the range of 4.0–5.5. As the compounds are insoluble in water, measurements were conducted in a 1:1 (v:v) acetonitrile:buffer mixture. Mixing the buffers with an equal volume of acetonitrile reduces the effective buffer concentration to 100 mM.

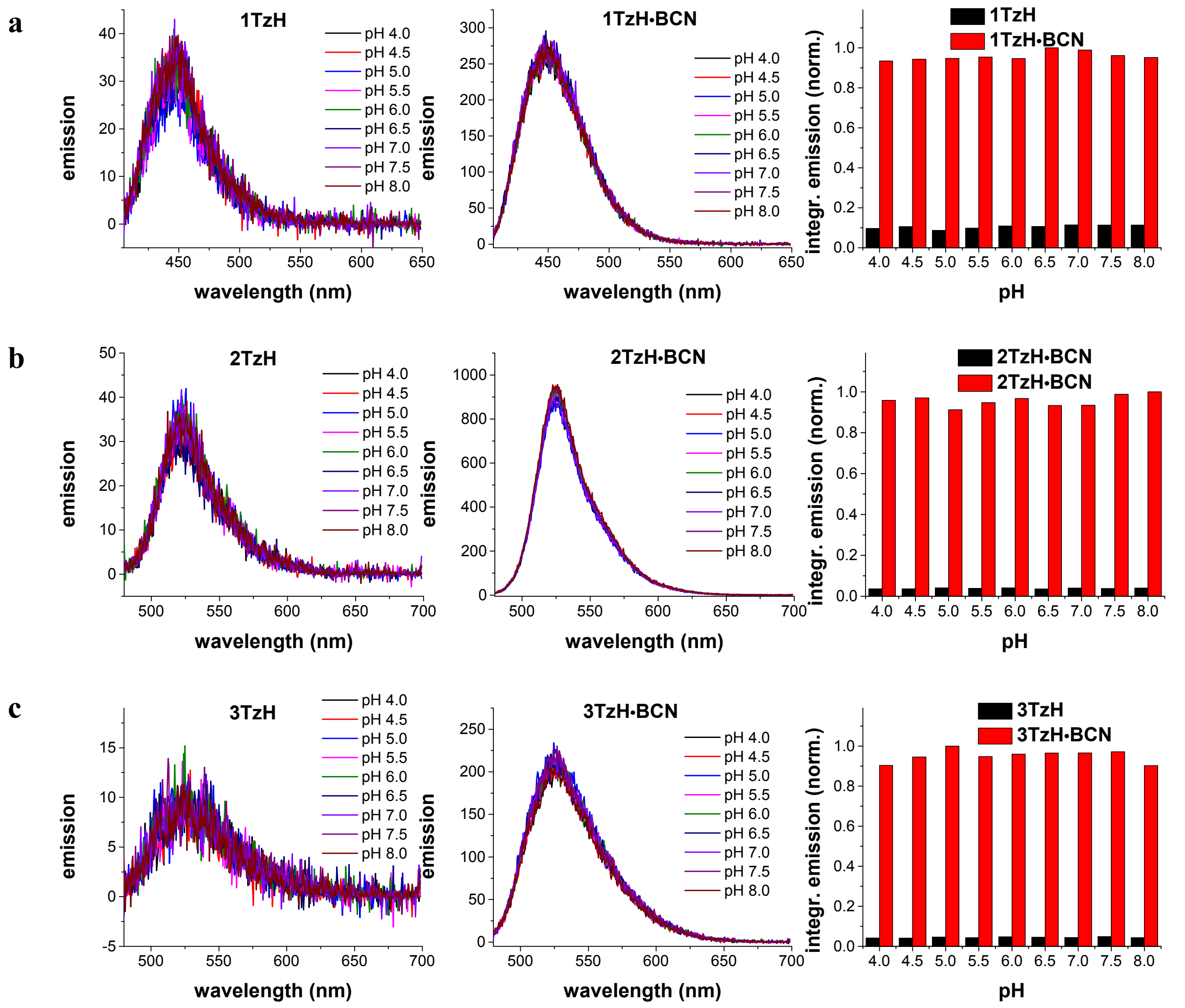

Figure 7S. pH dependency experiments conducted on H-terminated tetrazine dyes and their BCN adducts. Compounds **2TzH** and **2TzH•BCN** were at a 0.25 µM concentration, while the others were at 0.50 µM. The spectra were recorded in a 1:1 (v:v) mixture of acetonitrile and buffer.

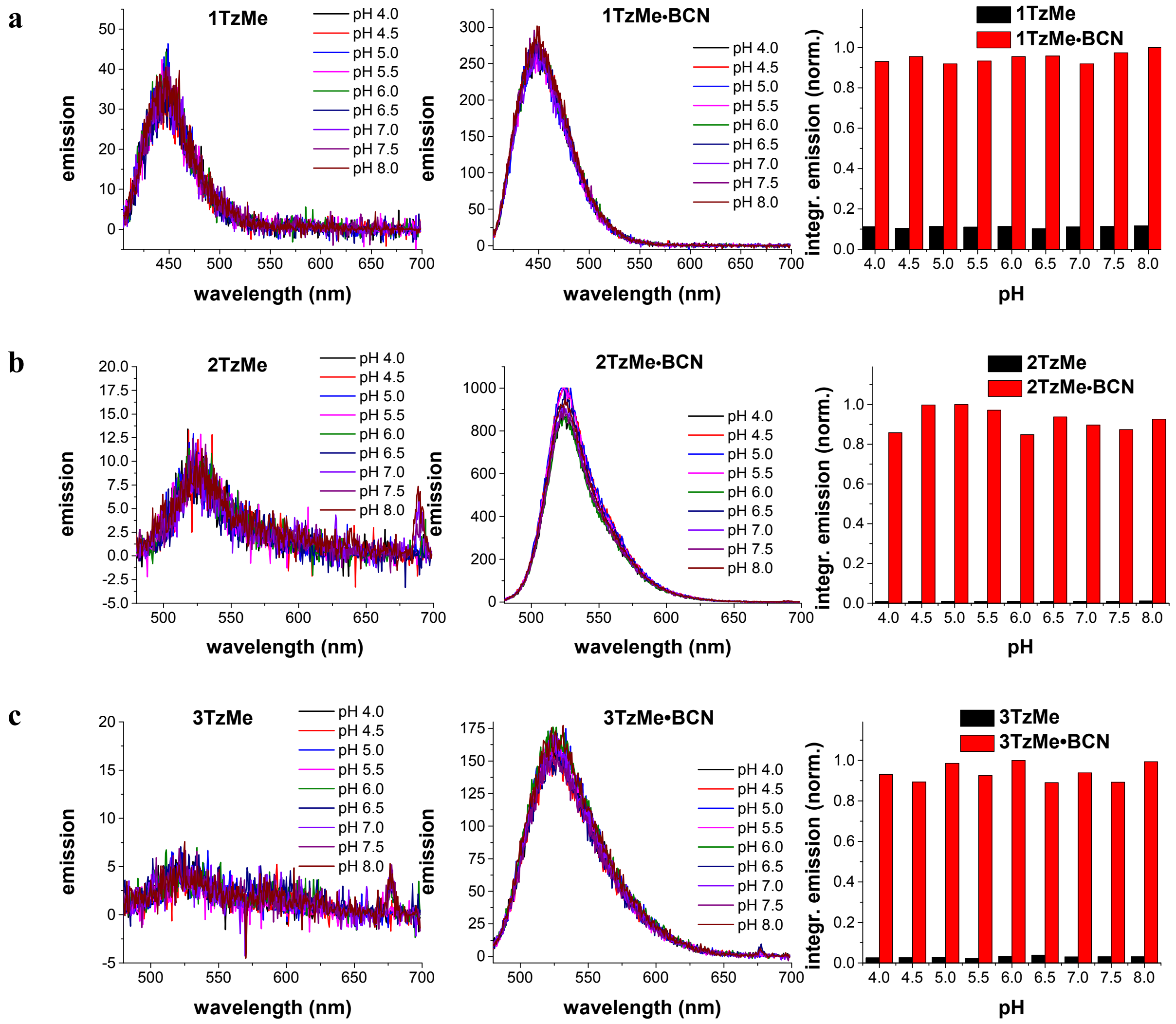

Figure 8S. pH dependency experiments conducted on Me-terminated tetrazine dyes and their BCN adducts. Compounds **2TzMe** and **2TzMe•BCN** were at a 0.25 µM concentration, while the others were at 0.50 µM. The spectra were recorded in a 1:1 (v:v) mixture of acetonitrile and buffer.

Time-dependent fluorescence emission of 1TzMe

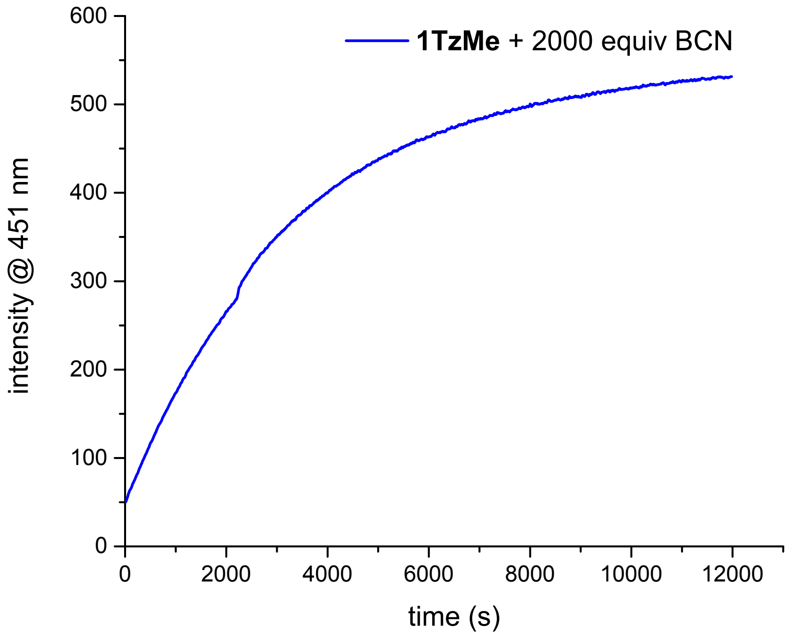

Figure 9S. The time-dependent fluorescence emission profile of the cycloaddition reaction between **1TzMe** (0.5 μM) and BCN (1.0 mM) in acetonitrile with 5% DMSO as a cosolvent at room temperature.

Table S2. A literature compilation of fluorophore–tetrazines that carry H- or Me-substituents on tetrazine units.

Fl–Tz	dienophile	medium	λ_{abs} [nm]	λ_{em} [nm]	ε_{max} [M^{-1} cm^{-1}]	Φ_{fl} (adduct)	turn-on	$\varepsilon_{max} \times \Phi_{fl}$ [$M^{-1}cm^{-1}$]	ref.[a]
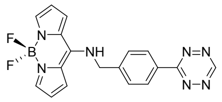	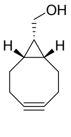 (BCN)	MeCN	401	451	30800	0.105	52 ×	3234	this work
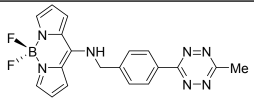	BCN	MeCN	401	452	21300	0.096	36 ×	2045	this work
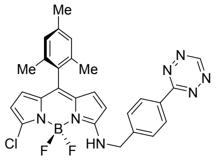	BCN	MeCN	473	524	28700	0.959	70 ×	27523	this work
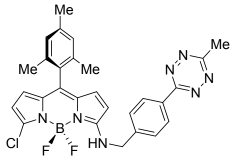	BCN	MeCN	473	527	29600	0.890	156 ×	26344	this work
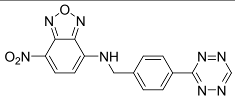	BCN	MeCN	456	520	19500	0.667	172 ×	13006	this work
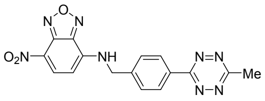	BCN	MeCN	457	521	16300	0.637	193 ×	10383	this work
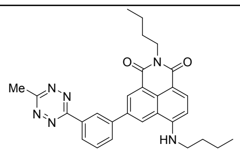	BCN	EtOH	461	540	8900	0.34	200 ×	3000	New and co-workers (2021)[18b]
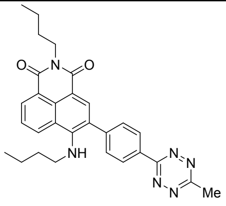	BCN	EtOH	449	545	11000	0.25	10 ×	2700	New and co-workers (2021)[18b]

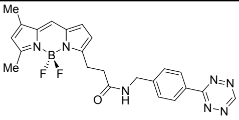	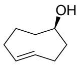<br>(TCO)	PBS, pH 7.4	505	512	-	0.24	15 ×	-	Weissleder and co-workers (2010)[23]
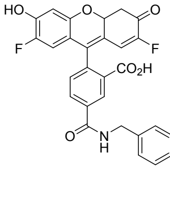	TCO	PBS, pH 7.4	495	523	-	0.82	18.5 ×	-	Weissleder and co-workers (2010)[23]
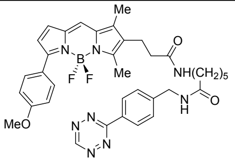	TCO	PBS, pH 7.4	543	573	-	0.40	20.6 ×	-	Weissleder and co-workers (2010)[23]
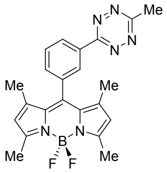	TCO	water (MeCN)	-	-	-	0.73<br>(0.58)	1600 ×<br>(1100 ×)	-	Weissleder and co-workers (2013)[10]
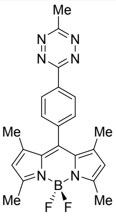	TCO	water (MeCN)	-	-	-	0.80<br>(0.23)	900 ×<br>(340 ×)	-	Weissleder and co-workers (2013)[10]
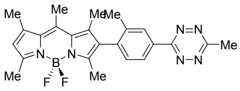	TCO	water (MeCN)	-	-	-	-<br>(0.22)	-<br>(120 ×)	-	Weissleder and co-workers (2013)[10]
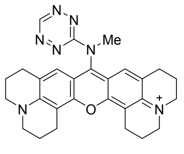	BCN<br>TCO<br>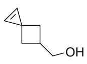<br>(Sph)	PBS	591<br>595<br>602	612<br>614<br>617	58000<br>40000<br>79000	0.62<br>0.50<br>0.66	612 ×<br>528 ×<br>626 ×	35960<br>20000<br>52140	Wu and co-workers (2022)[31b]
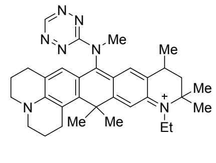	BCN<br>TCO<br>Sph	PBS	657<br>660<br>664	671<br>675<br>676	83000<br>82000<br>105000	0.47<br>0.37<br>0.38	1459 ×<br>544 ×<br>668 ×	39010<br>30340<br>39900	Wu and co-workers (2022)[31b]
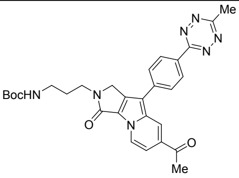	TCO	MeCN:H_2O (1:1)	416	532	-	0.168	20 ×	-	Kim&Park and co-workers (2018)[16]

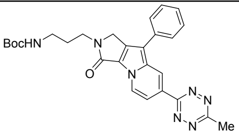	TCO	MeCN:H_2O (1:1)	375	484	-	0.683	>1000 ×	-	Kim&Park and co-workers (2018)[16]
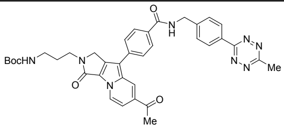	TCO	MeCN:H_2O (1:1)	401	522	-	0.583	14 ×	-	Kim&Park and co-workers (2018)[16]
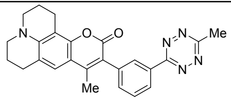	TCO	PBS, pH 7.4	400	502	16000	0.41	4000 ×	6560	Weissleder and co-workers (2014)[11]
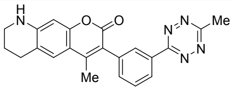	TCO	PBS, pH 7.4	388	482	20000	0.38	11000 ×	7600	Weissleder and co-workers (2014)[11]
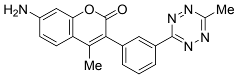	TCO	PBS, pH 7.4	370	463	19000	0.49	2900 ×	9310	Weissleder and co-workers (2014)[11]
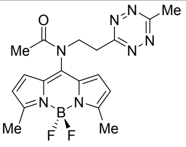	BCN<br>TCO<br>Sph	EtOH	521<br>520<br>510	529<br>530<br>522	30200<br>28400<br>25200	0.85<br>0.87<br>0.84	62 ×<br>65 ×<br>128 ×	25670<br>24708<br>21168	Wu and co-workers (2022)[35]
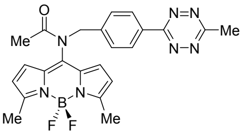	BCN<br>TCO<br>Sph	EtOH	521<br>521<br>522	531<br>531<br>531	44100<br>49400<br>39400	0.91<br>0.28<br>0.74	59 ×<br>10 ×<br>28 ×	40131<br>13832<br>29156	Wu and co-workers (2022)[35]
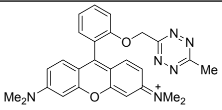	BCN	PBS, pH 7.4	557	582	74100	0.447	95 ×	33123	Wombacher and co-workers (2021)[31a]
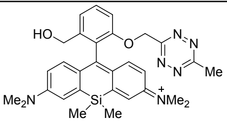	BCN	PBS, pH 3.5	656	677	104000	0.310	9 ×	32240	Wombacher and co-workers (2021)[31a]
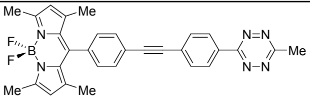	TCO	chloroform	323,<br>505	518	-	-	11.9 ×	-	Wombacher and co-workers (2014)[19]
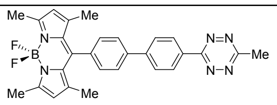	TCO	chloroform	303,<br>504	515	-	-	23.4 ×	-	Wombacher and co-workers (2014)[19]
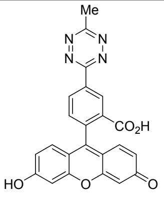	TCO	PBS, pH 7.4	495	521	57000	-	72 ×	-	Wombacher and co-workers (2017)[20]

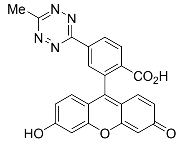	TCO	PBS, pH 7.4	495	517	55000	-	109 ×	-	Wombacher and co-workers (2017)[20]
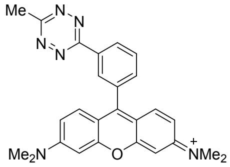	TCO	PBS, pH 7.4	554	577	54000	-	12 ×	-	Wombacher and co-workers (2017)[20]
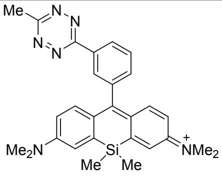	TCO	PBS, pH 7.4	649	664	62000	-	3.7 ×	-	Wombacher and co-workers (2017)[20]
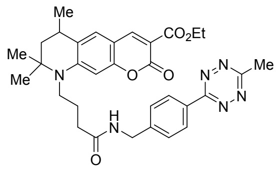	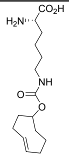 (TCO-Lys)	PBS, pH 7.4	441	478	-	-	15 ×	-	Sauer and co-workers (2019)[29]
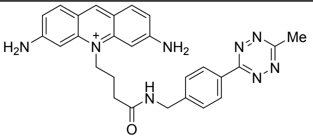	TCO-Lys	PBS, pH 7.4	455	505	-	-	39 ×	-	Sauer and co-workers (2019)[29]
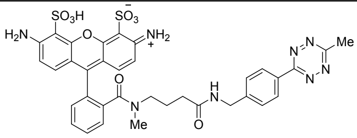	TCO-Lys	PBS, pH 7.4	502	522	-	-	25 ×	-	Sauer and co-workers (2019)[29]
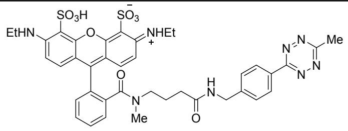	TCO-Lys	PBS, pH 7.4	535	553	-	-	7 ×	-	Sauer and co-workers (2019)[29]
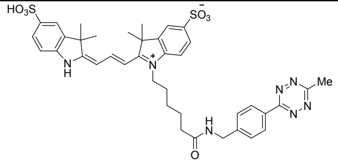	TCO-Lys	PBS, pH 7.4	551	564	-	-	2 ×	-	Sauer and co-workers (2019)[29]
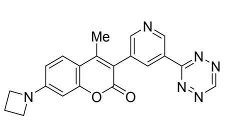	TCO<br>BCN	PBS, pH 7.4	371<br>371	485<br>480	8500<br>11500	-	1050 ×<br>500 ×	-	Vrabel and co-workers (2020)[18a]
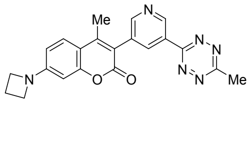	TCO<br>BCN	PBS, pH 7.4	370<br>372	485<br>475	23000<br>22000	-	3300 × 3400	-	Vrabel and co-workers (2020)[18a]

							×		
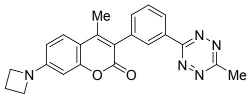	TCO<br>BCN	PBS,<br>pH 7.4	374<br>376	483<br>472	16000<br>21000	-	2900<br>×<br>3100<br>×	-	Vrabel and co-workers (2020)[18a]
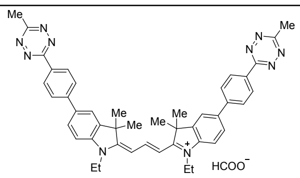	BCN	PBS,<br>pH 7.4<br>0.1% SDS	582	603	111000	0.289	13.3 ×	32079	Lemke&Kele and co-workers (2018)[17d]
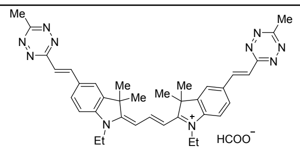	BCN	PBS,<br>pH 7.4<br>0.1% SDS	599	619	93000	0.157	14.8 ×	14601	Lemke&Kele and co-workers (2018)[17d]
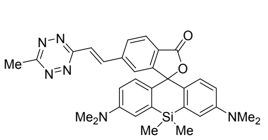	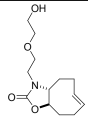<br>(OxTCO)	water<br>0.1% SDS	645	667	19200	0.34	22 ×	6528	Kele and co-workers (2017)[17e]
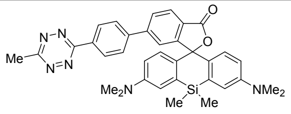	OxTCO	water<br>0.1% SDS	644	660	21600	0.34	31 ×	7344	Kele and co-workers (2017)[17e]
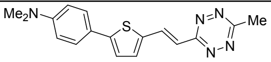	BCN	EtOH	401	587	26000	0.29	265 ×	7500	Wu and co-workers (2021)[37]
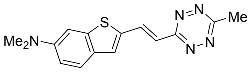	BCN	EtOH	393	556	31000	0.14	186 ×	4300	Wu and co-workers (2021)[37]
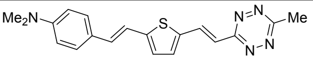	BCN	EtOH	433	642	36000	0.30	961 ×	10800	Wu and co-workers (2021)[37]
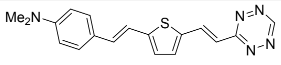	BCN	EtOH	434	652	27000	0.14	304 ×	3800	Wu and co-workers (2021)[37]

*a*For citations, refer to the original manuscript of this SI file.

Protein labelling studies

Synthesis and characterization of the amine-reactive BCN–PNP

We prepared the compound **BCN–PNP** by reacting BCN with 4-nitrophenyl chloroformate in the presence of pyridine in DCM by following the protocol by Isaacman and coworkers.[7] Despite all our efforts to characterize **BCN–PNP** with HRMS, we only observed the nitrophenolate fragment (Figure 6S). Consequently, this compound was converted into a chemically more robust carbamate derivative (**BCN–PNP**)[11] to enable indirect characterization by HRMS. **Data for BCN–PNP**: HRMS (APCI positive) m/z: $[M + H]^+$ Calcd for $C_{17}H_{26}N_2O_3$ 307.2022, Found 307.2020.

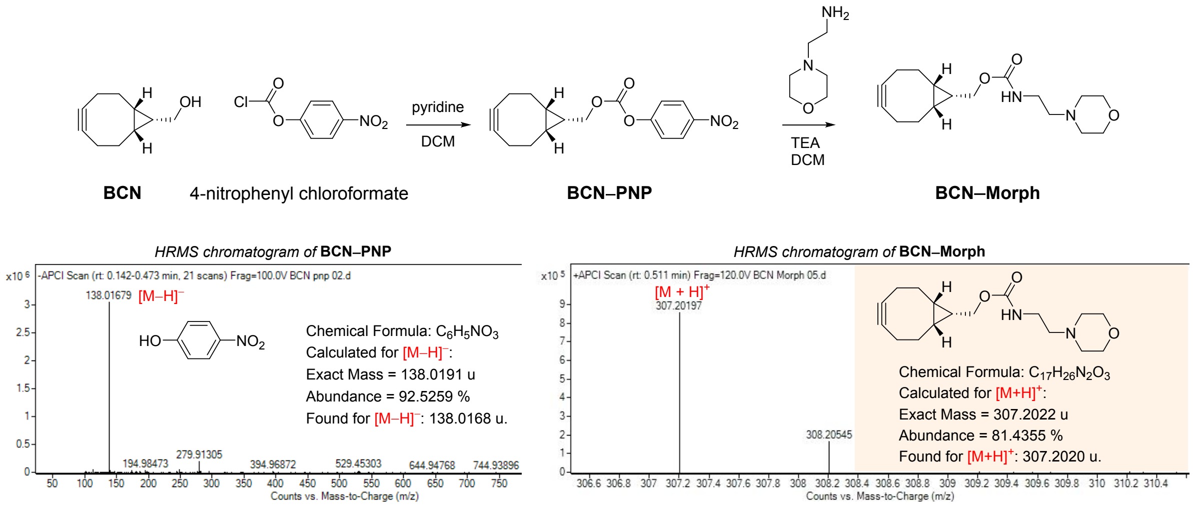

Figure 10S. Synthesis and characterization of the amine-reactive **BCN–PNP**.

BCN modification of HSA by BCN–PNP

To a 600 µL aliquot of a high-purity (lyophilized, assay ≥99%) Human Serum Albumin (HSA; 30 µM) solution, prepared by dissolving 10.3 mg HSA in 5.15 mL NaH_2PO_4 buffer (100 µM, pH 8.5), a 90 µL (5 equiv) DMSO solution of **BCN–PNP** (1.0 mM) was added to have a final concentration of 130 µM **BCN–PNP**. This mixture was incubated for 8 hours in the dark at room temperature on a shaker. The resulting **HSA–BCN** mixture was loaded onto Zeba™ Spin Desalting Columns (40K MWCO) to remove excess small molecules. Separately, 50 µL aliquots of BCN-tagged HSA samples and native HSA (a negative control) were incubated with 1mM DMSO stock solutions (15 µL, 11.5 equiv) of tetrazine probes (**1TzH**, **2TzH** and **3TzH**) for 1 hour at room temperature. The labelled protein samples were then purified again using spin column filtration. Subsequently, the samples were treated with an equal amount of sample buffer and denatured by boiling at 95ºC for 5 minutes. Once cooled, the samples were loaded onto 10% Tris-Glycine gel and electrophoresed using a Mini Gel Tank (Invitrogen). Following electrophoresis, fluorescent gel images were captured using Thermo iBright CL1000 (Figure

8S). After obtaining the fluorescent images, the gels were further stained with Coomassie Blue and visualized using Thermo iBright CL1000.

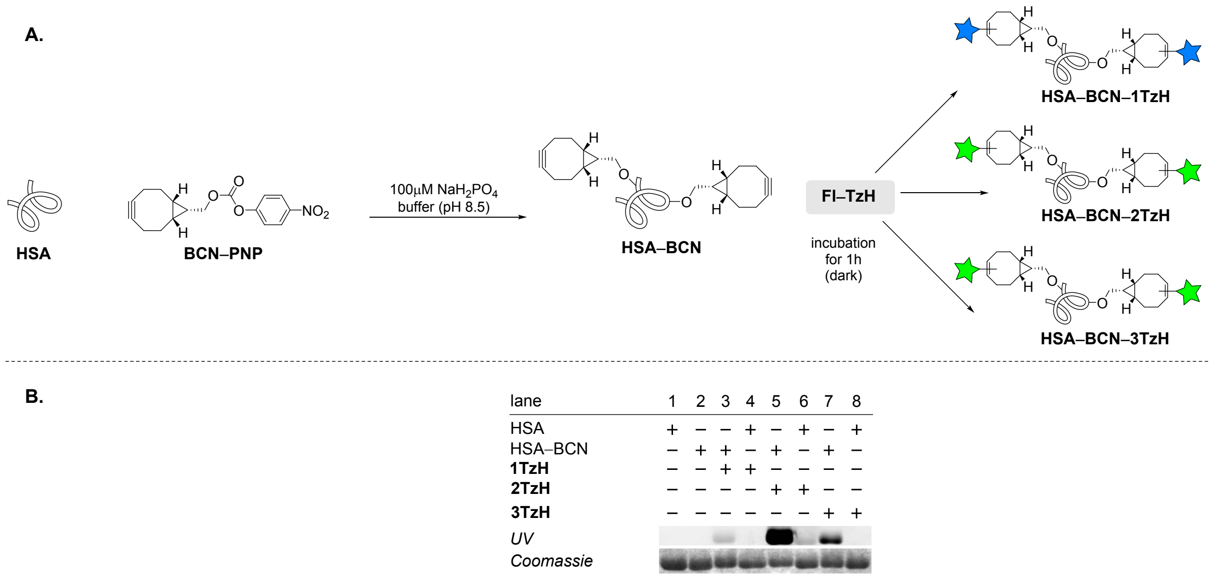

Figure 11S. Tagging HSA protein with BCN-PNP and labeling studies with H-terminated tetrazines (**A.**). In gel (SDS-PAGE) visualization of labelling experiments including negative controls. Digital photographs of fluorescent bands (top) and Coomassie Blue stained gel (bottom) (**B.**).

Copies of NMR, HRMS and FTIR spectra

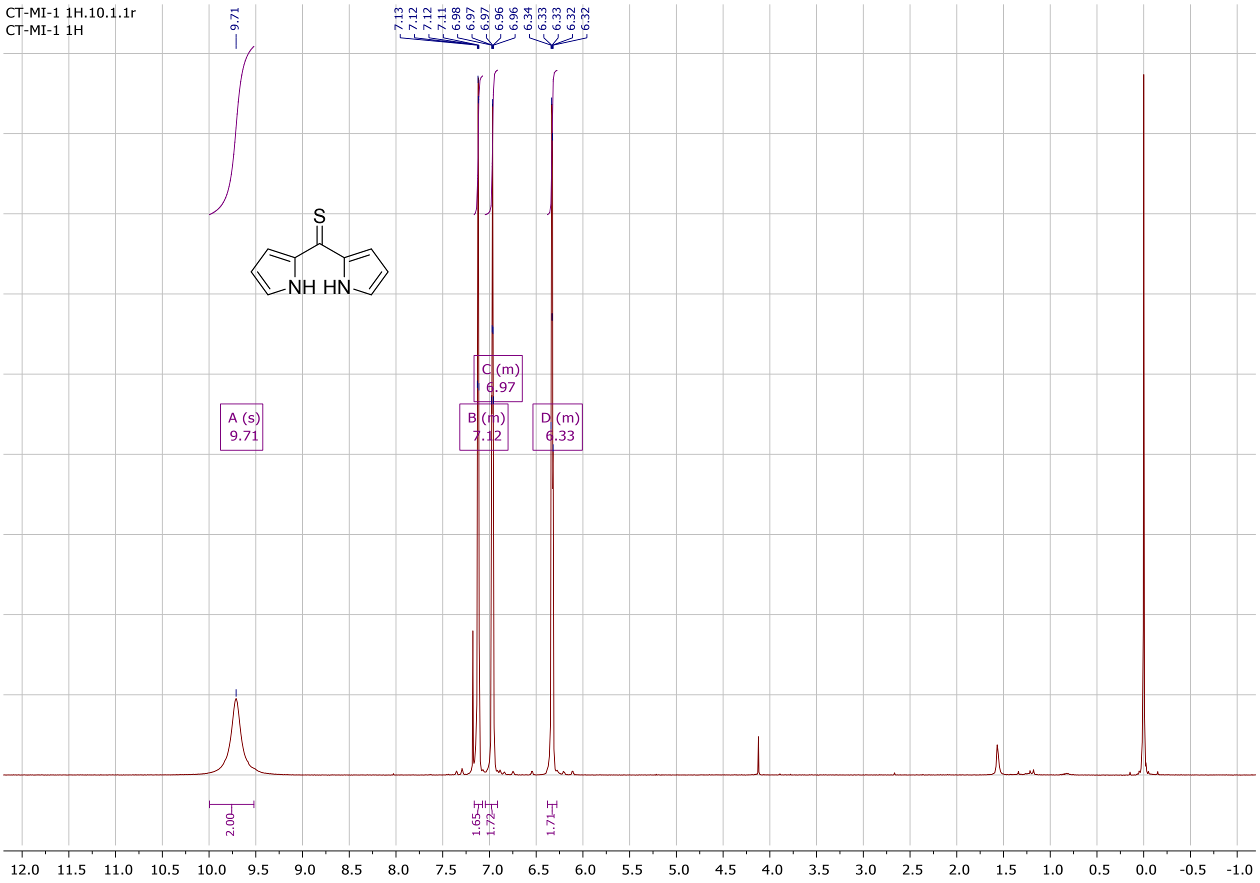

Figure 12S. ^{1}H NMR spectrum (400 MHz) of **2,2′-Dipyrrilthione** in $CDCl_3$.

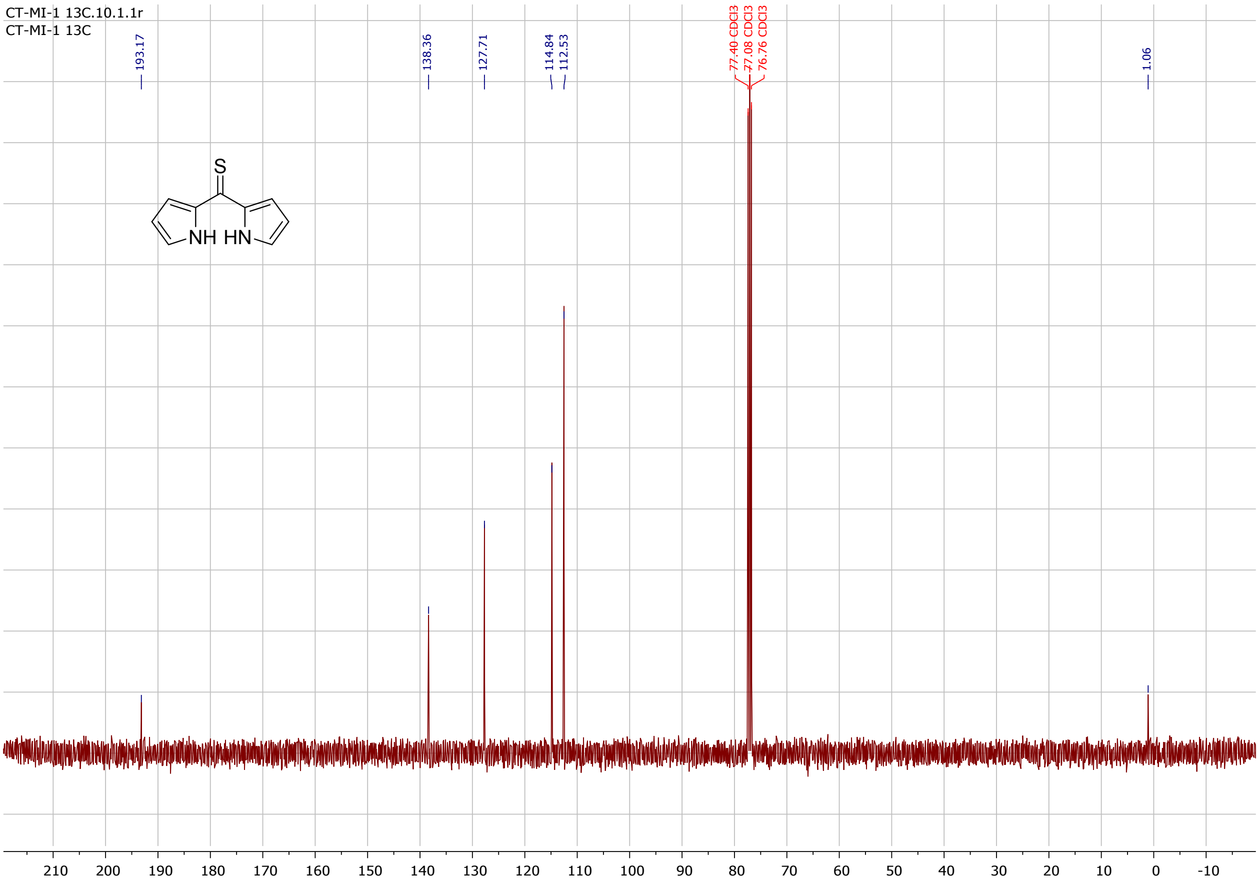

Figure 13S. $^{13}C\{^{1}H\}$ NMR spectrum (101 MHz) of **2,2′-Dipyrrilthione** in $CDCl_3$.

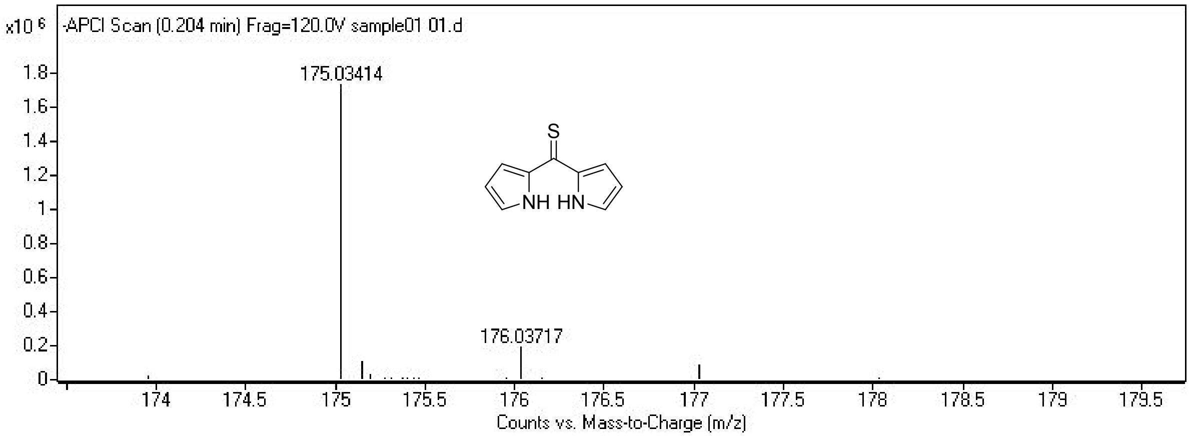

Figure 14S. HRMS (APCI negative) spectrum of **2,2′-Dipyrrilthione**.

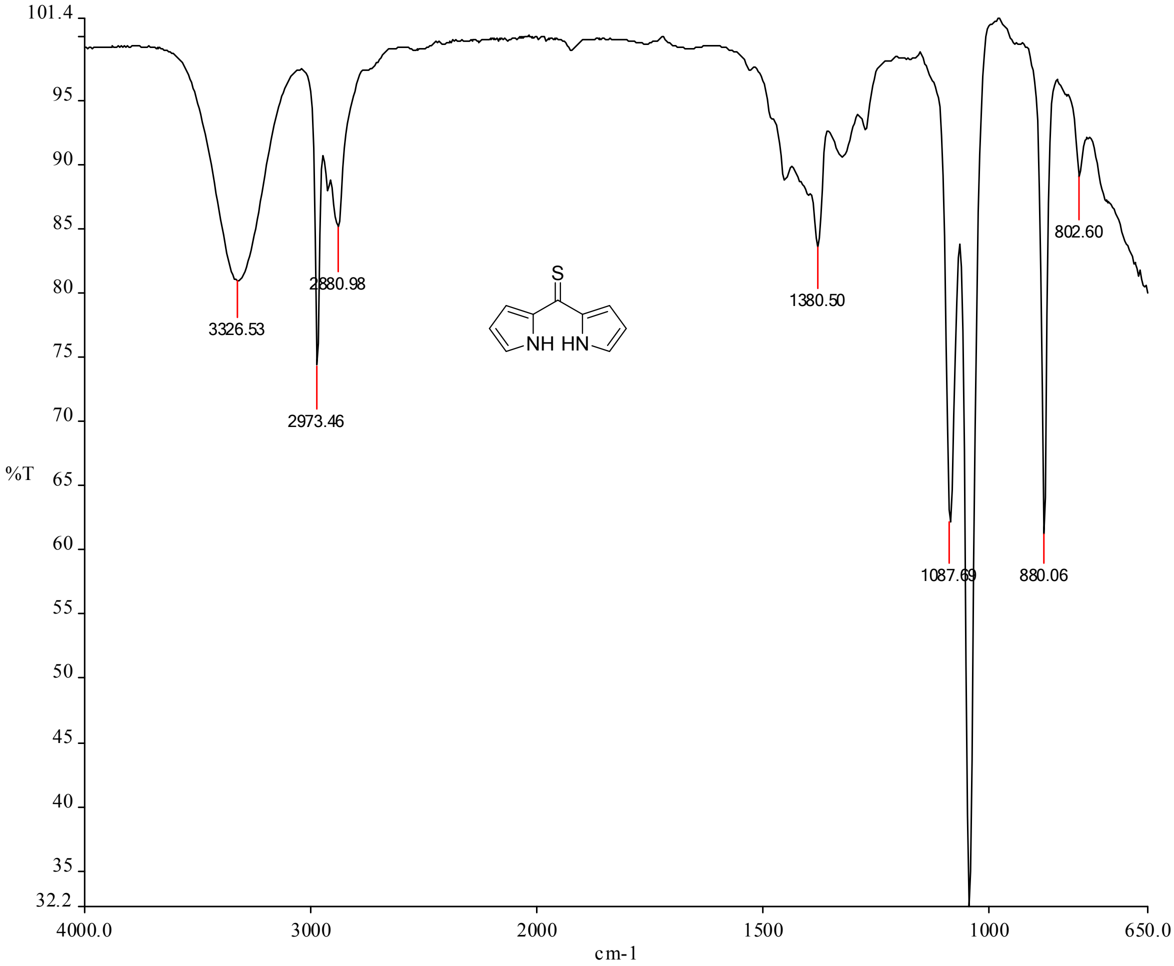

Figure 15S. IR (neat) spectrum of **2,2′-Dipyrrilthione**.

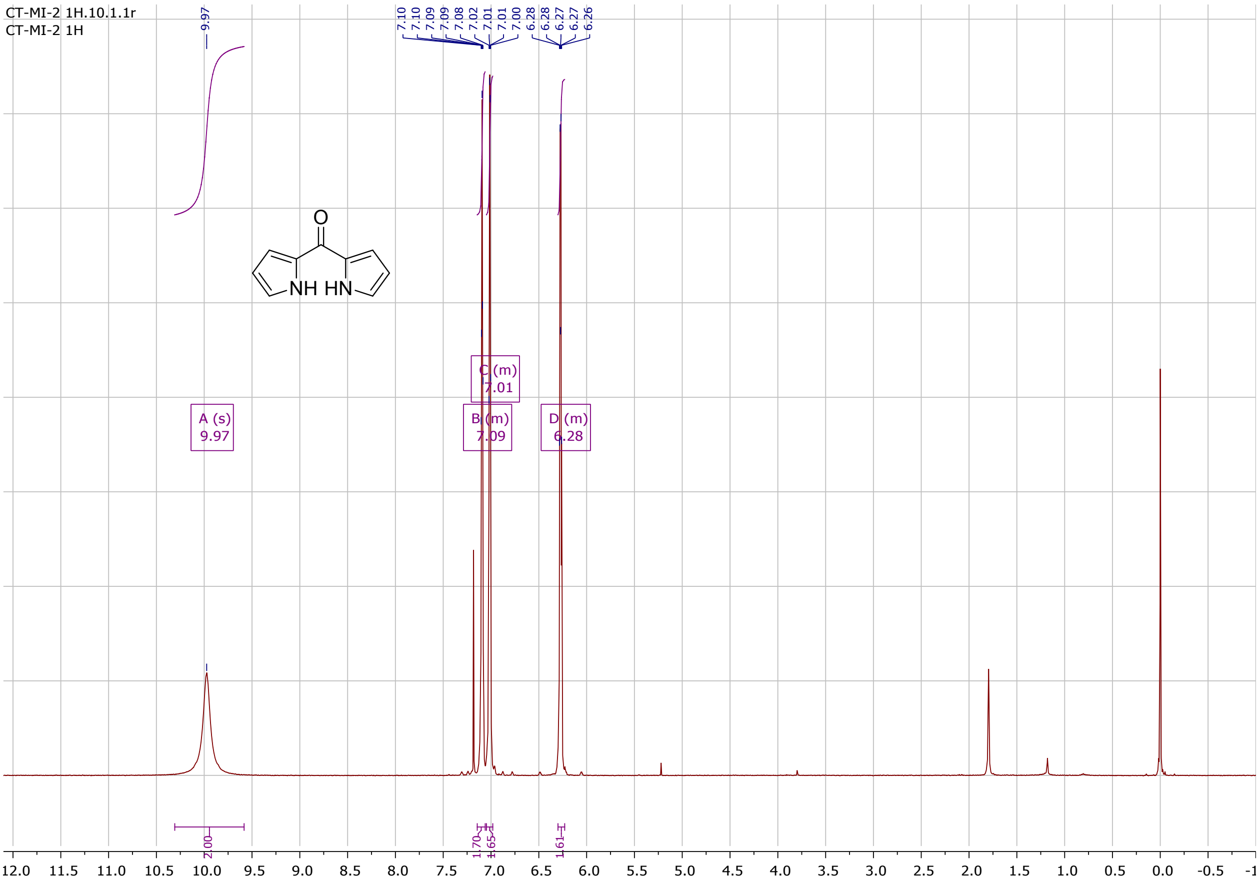

Figure 16S. ^{1}H NMR spectrum (400 MHz) of **2,2′-Dipyrrilketone** in $CDCl_3$.

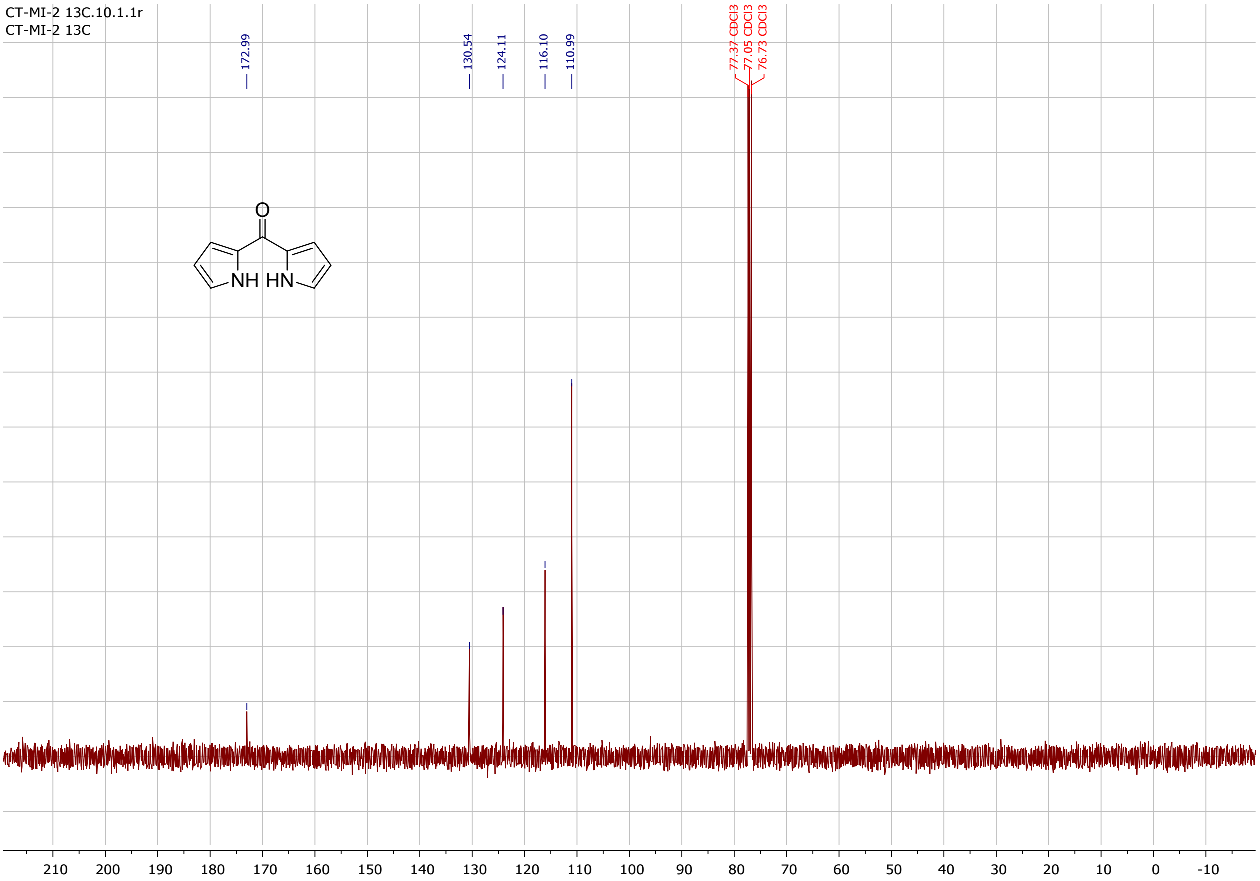

Figure 17S. $^{13}C\{^{1}H\}$ NMR spectrum (101 MHz) of **2,2′-Dipyrrilketone** in $CDCl_3$.

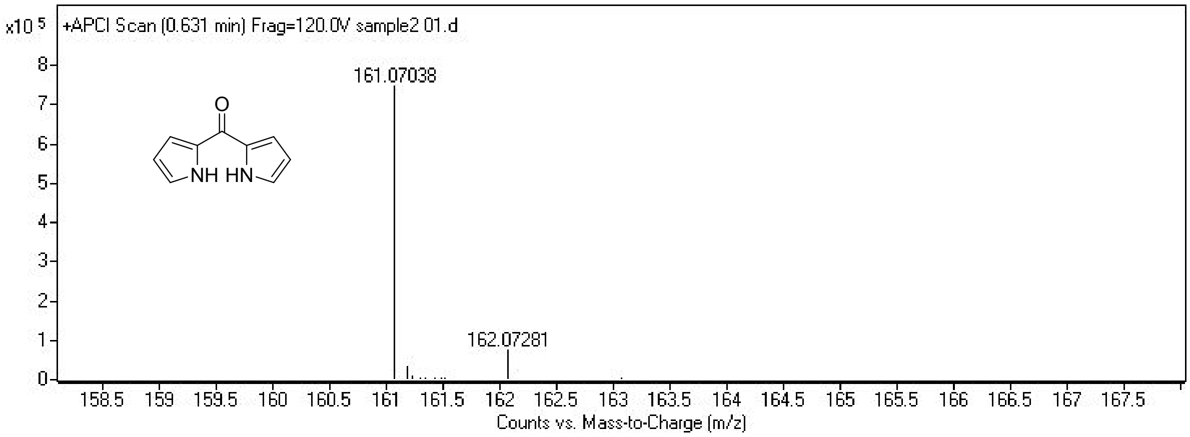

Figure 18S. HRMS (APCI positive) spectrum of **2,2′-Dipyrrilketone**.

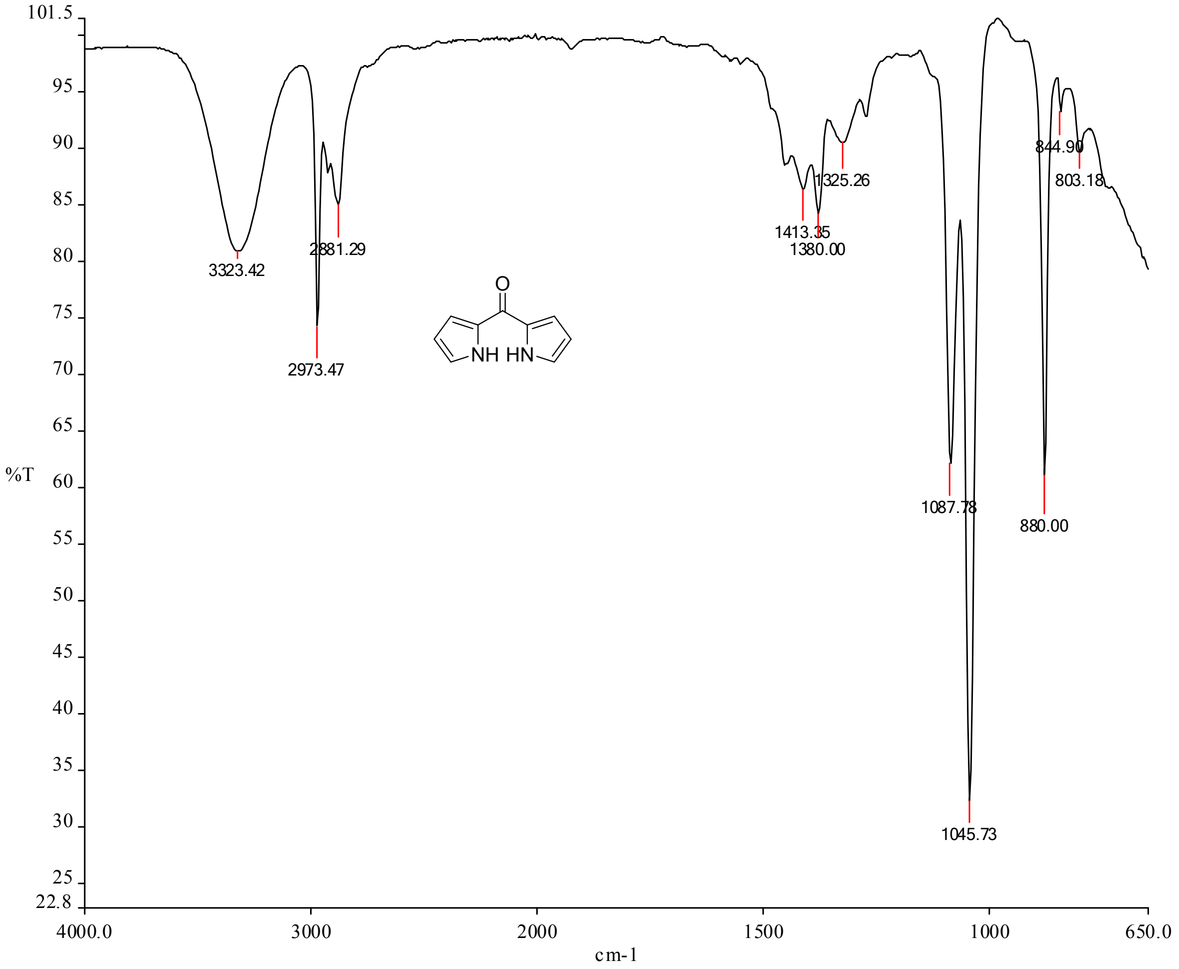

Figure 19S. IR (neat) spectrum of **2,2′-Dipyrrilketone**.

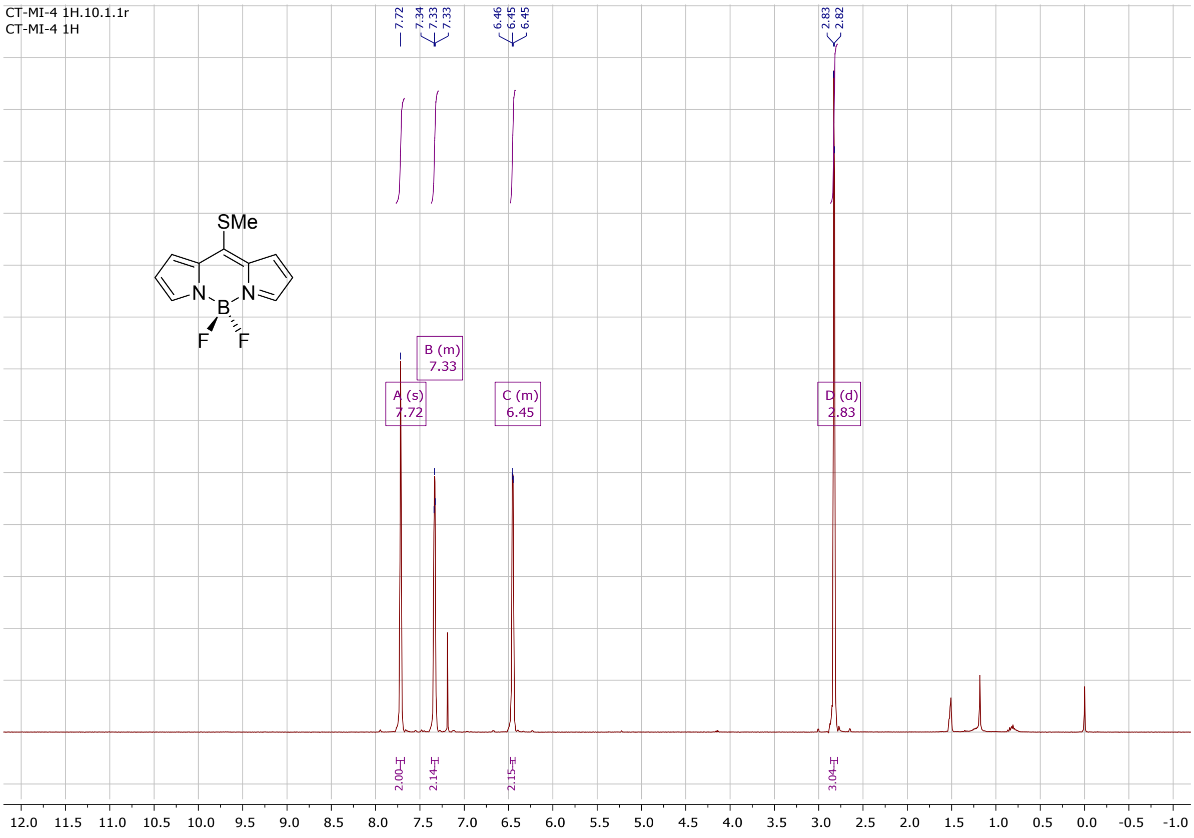

Figure 20S. ^{1}H NMR spectrum (400 MHz) of **1a** in $CDCl_3$.

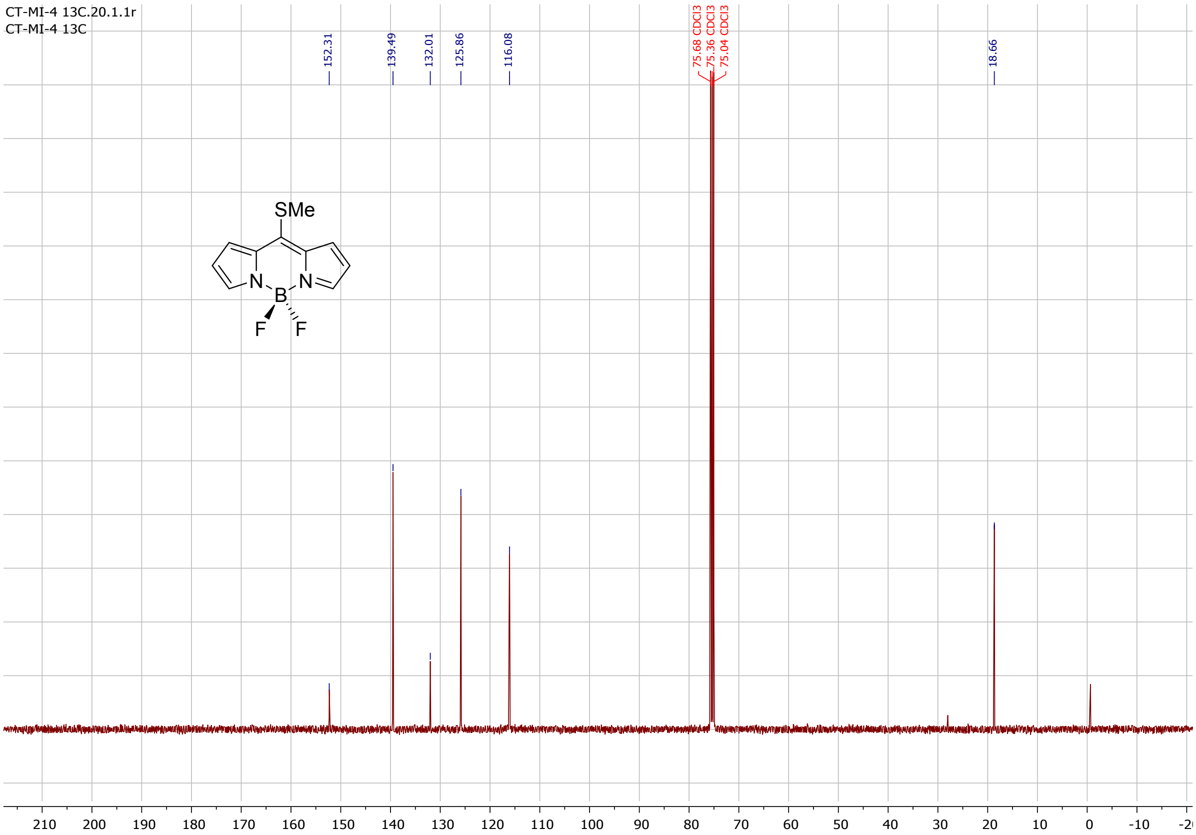

Figure 21S. $^{13}C\{^{1}H\}$ NMR spectrum (101 MHz) of **1a** in $CDCl_3$.

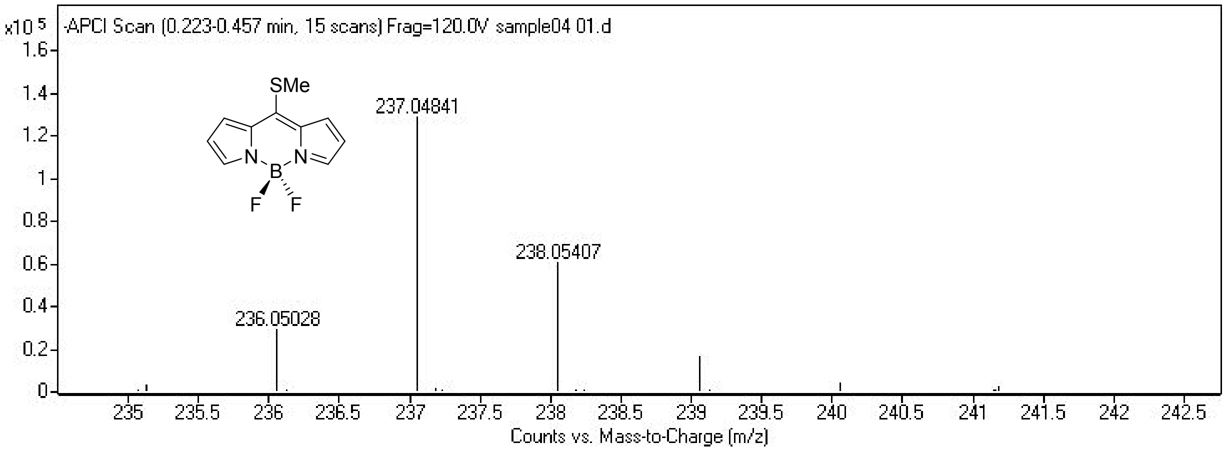

Figure 22S. HRMS (APCI negative) spectrum of **1a**.

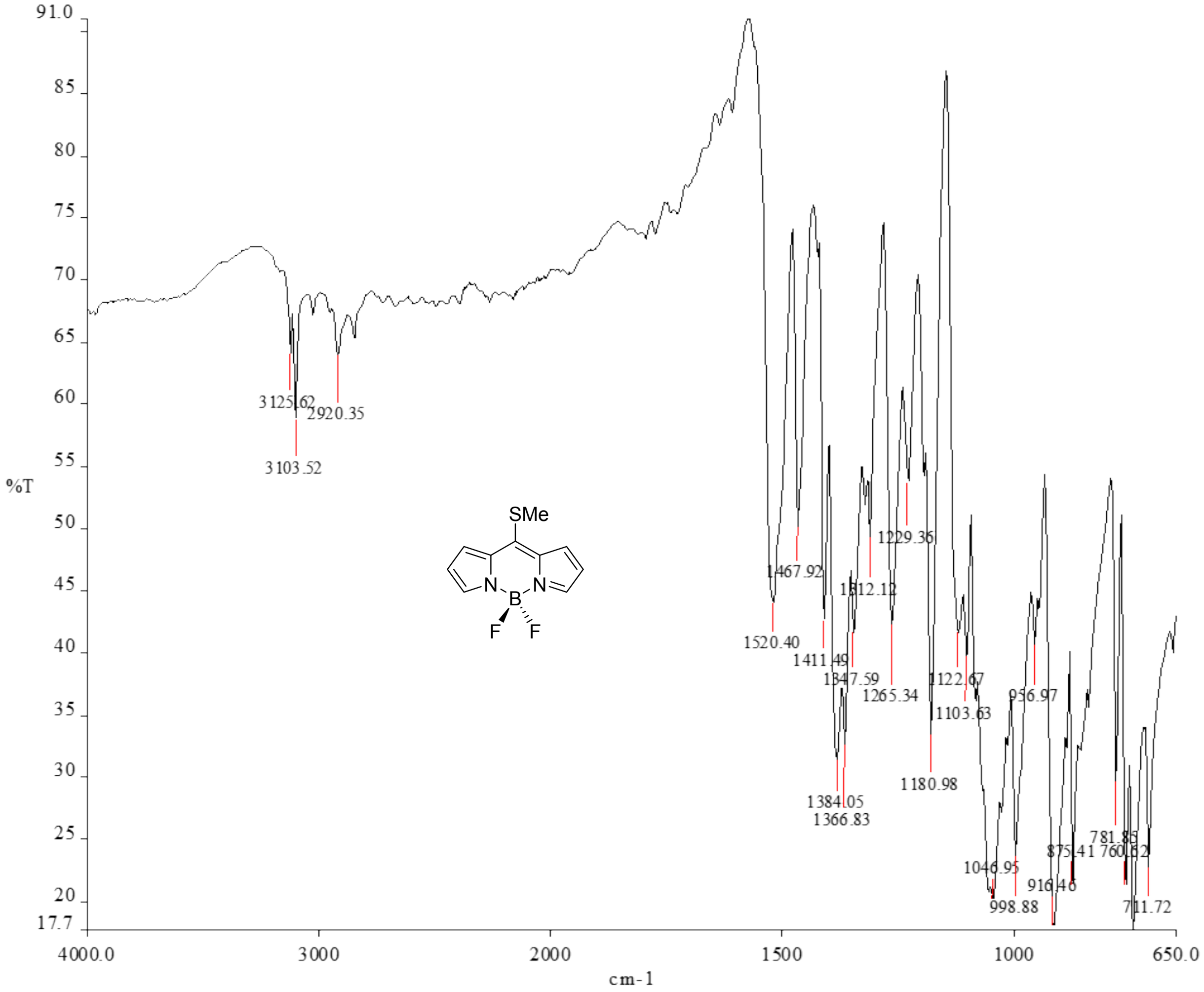

Figure 23S. IR (neat) spectrum of **1a**.

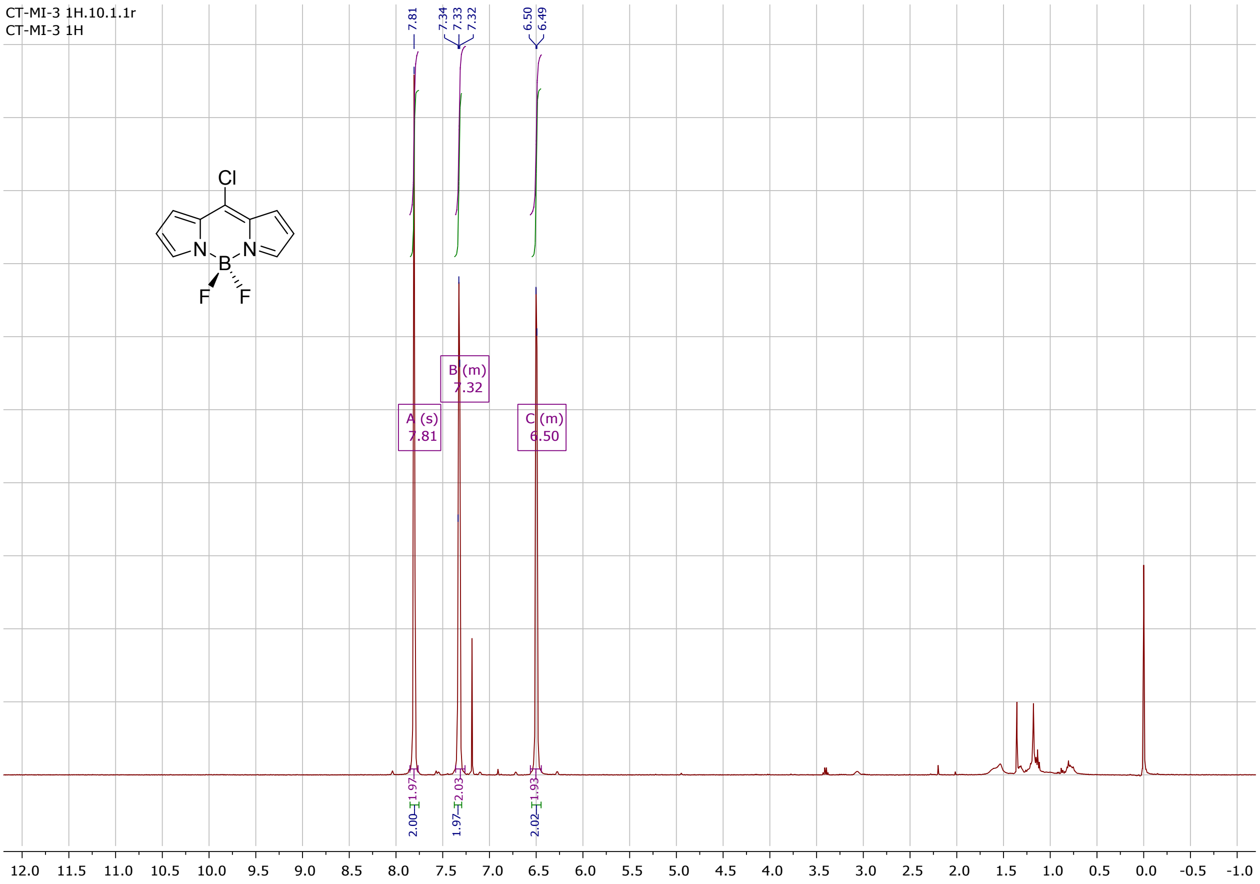

Figure 24S. ^{1}H NMR spectrum (400 MHz) of **1b** in $CDCl_3$.

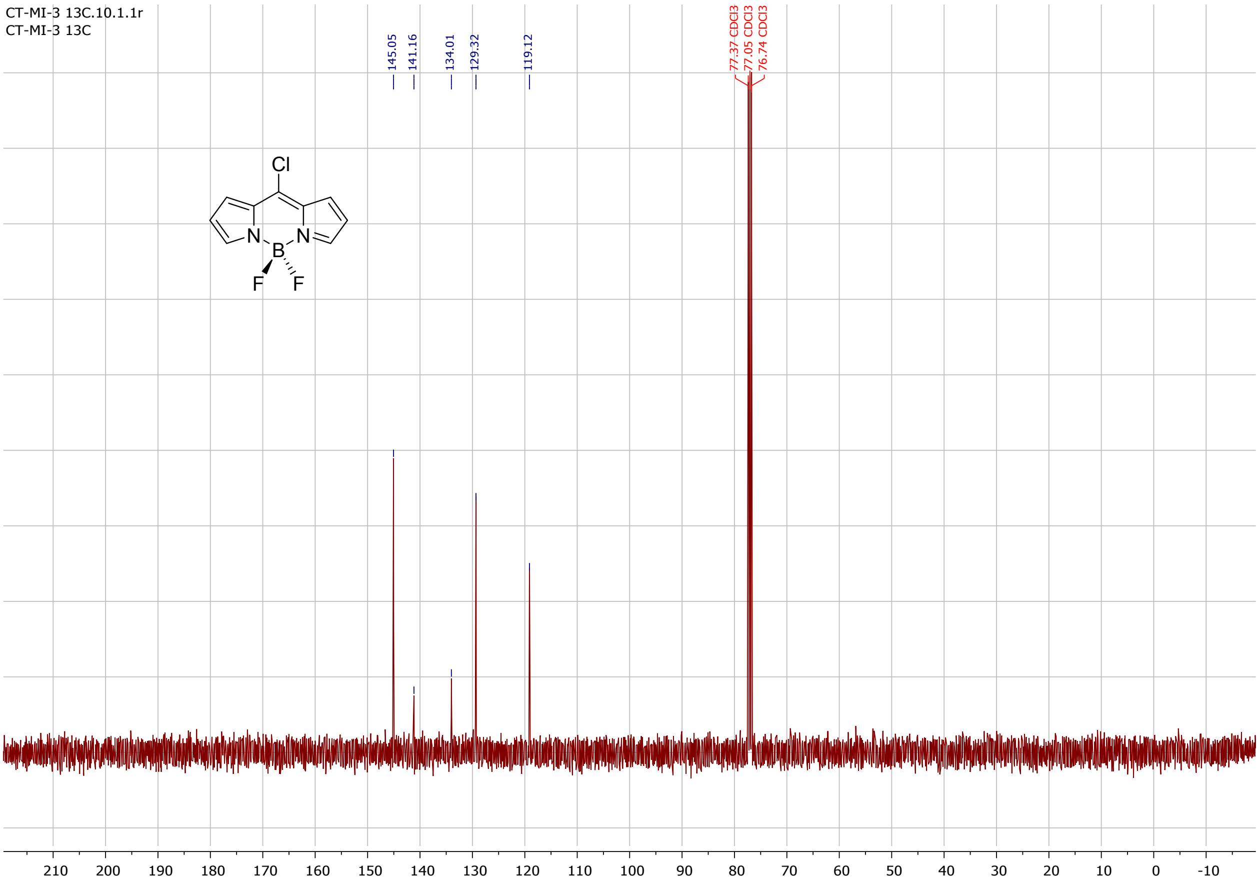

Figure 25S. $^{13}C\{^{1}H\}$ NMR spectrum (101 MHz) of **1b** in $CDCl_3$.

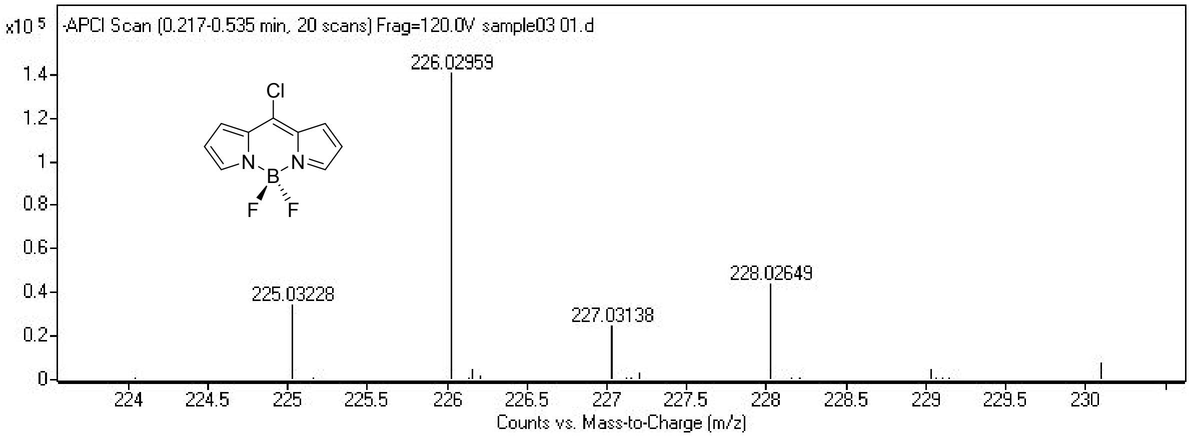

Figure 26S. HRMS (APCI negative) spectrum of **1b**.

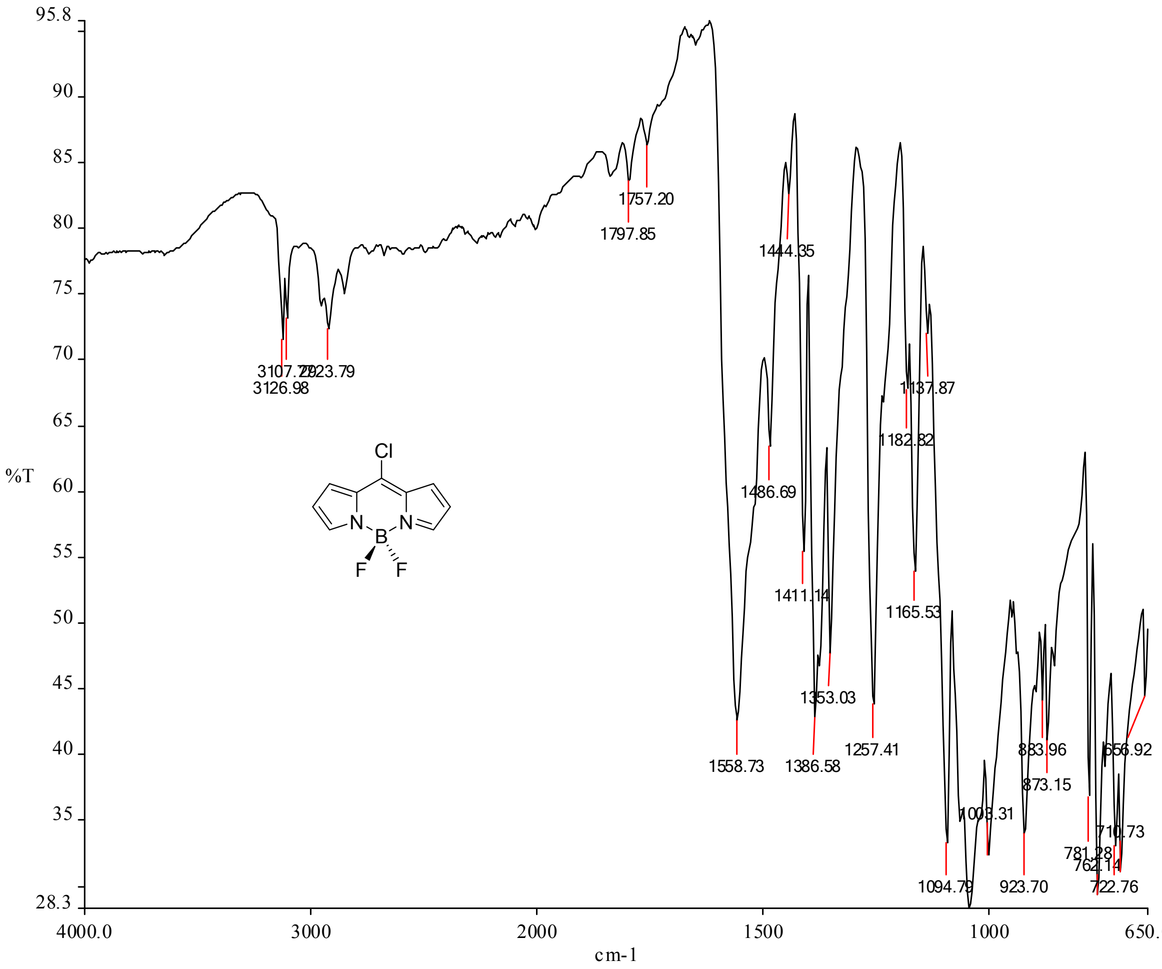

Figure 27S. IR (neat) spectrum of **1b**.

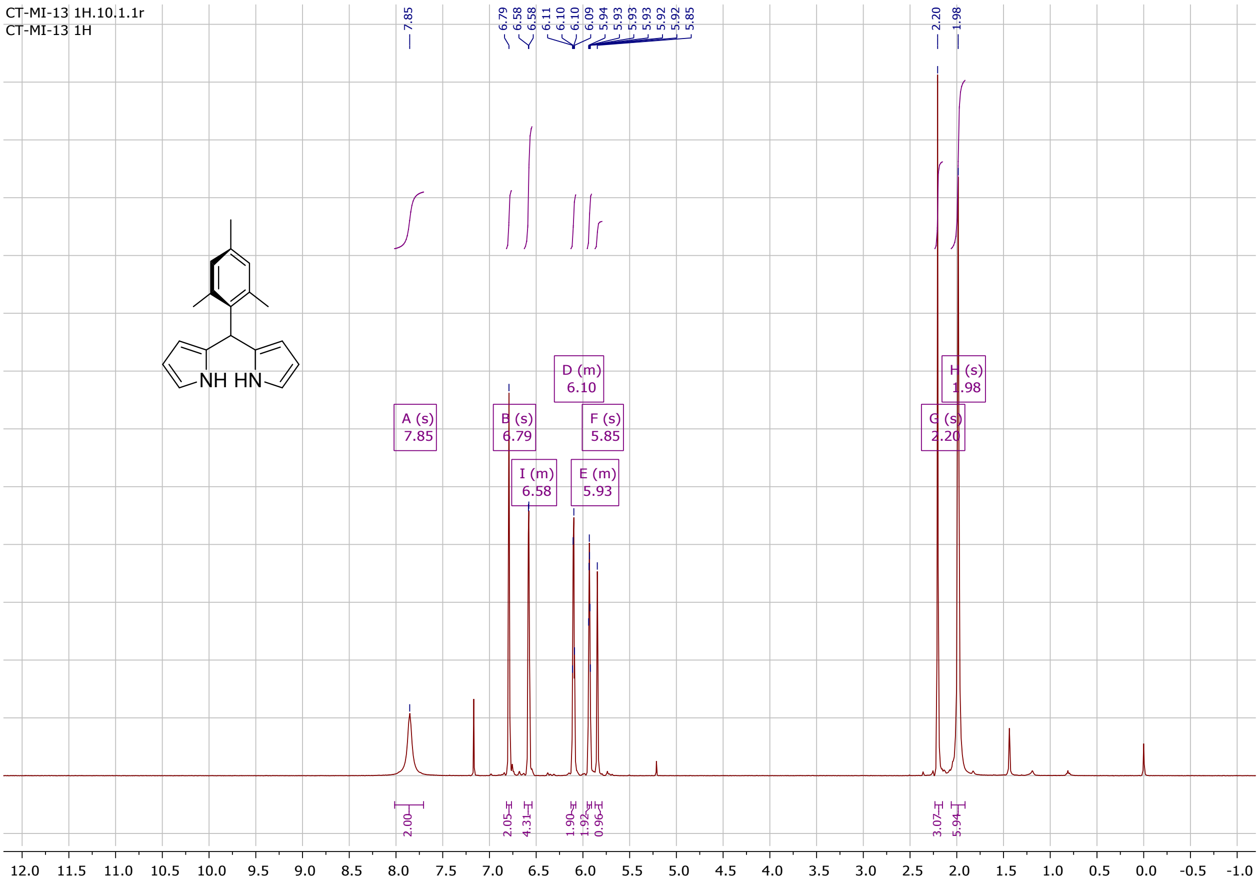

Figure 28S. ^{1}H NMR spectrum (400 MHz) of **2,2′-(mesitylmethylene)bis(1H-pyrrole)** in $CDCl_3$.

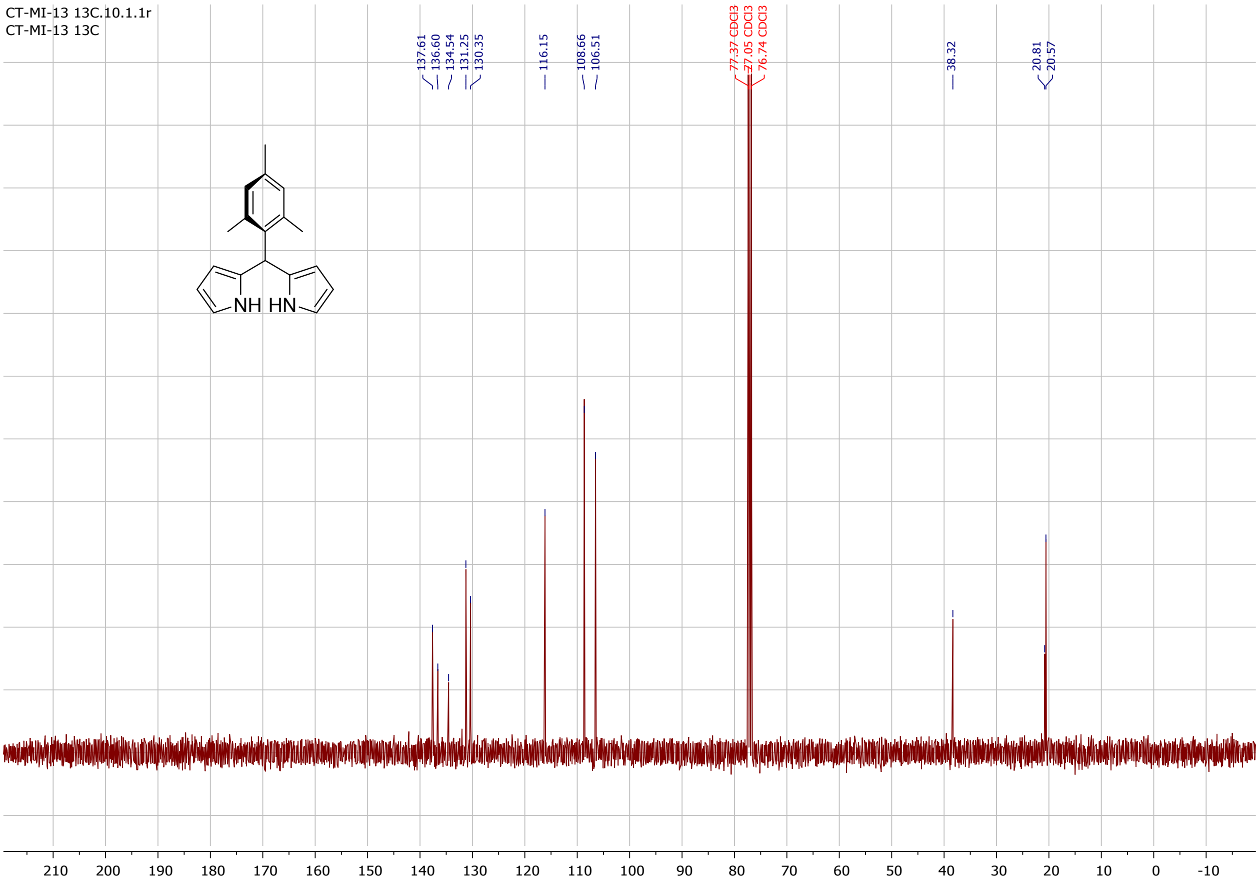

Figure 29S. $^{13}C\{^{1}H\}$ NMR spectrum (101 MHz) of **2,2′-(mesitylmethylene)bis(1H-pyrrole)** in $CDCl_3$.

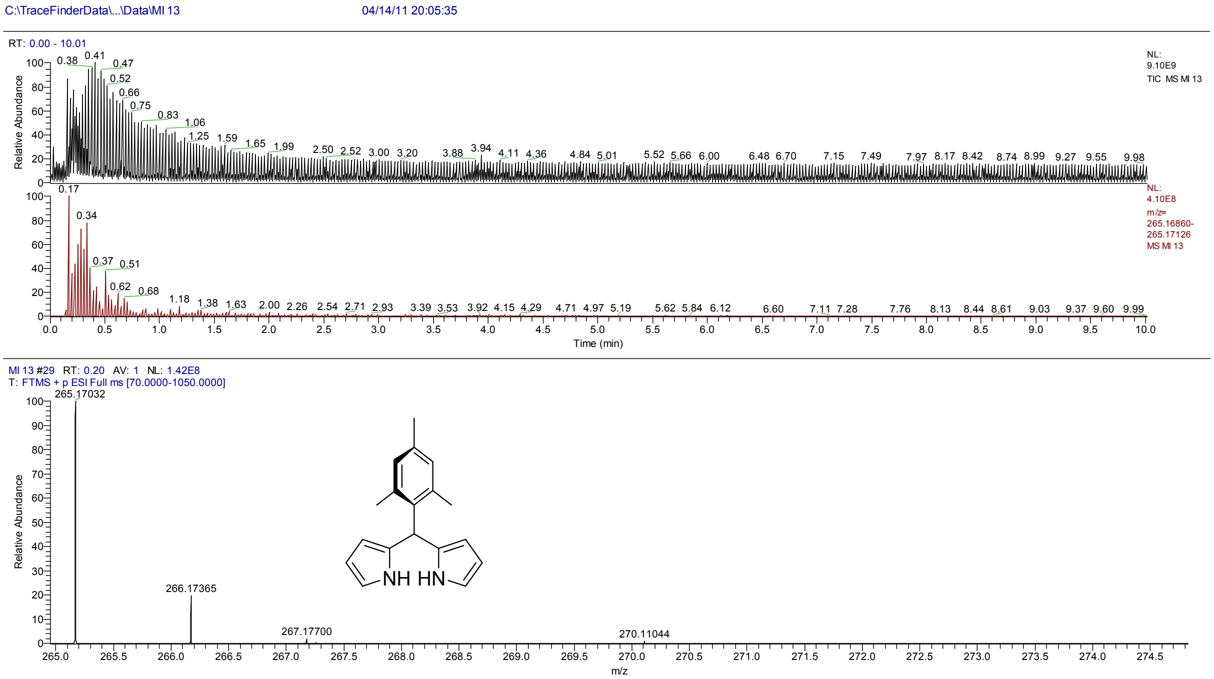

Figure 30S. HRMS (ESI positive) spectrum of **2,2′-(mesitylmethylene)bis(1H-pyrrole)**.

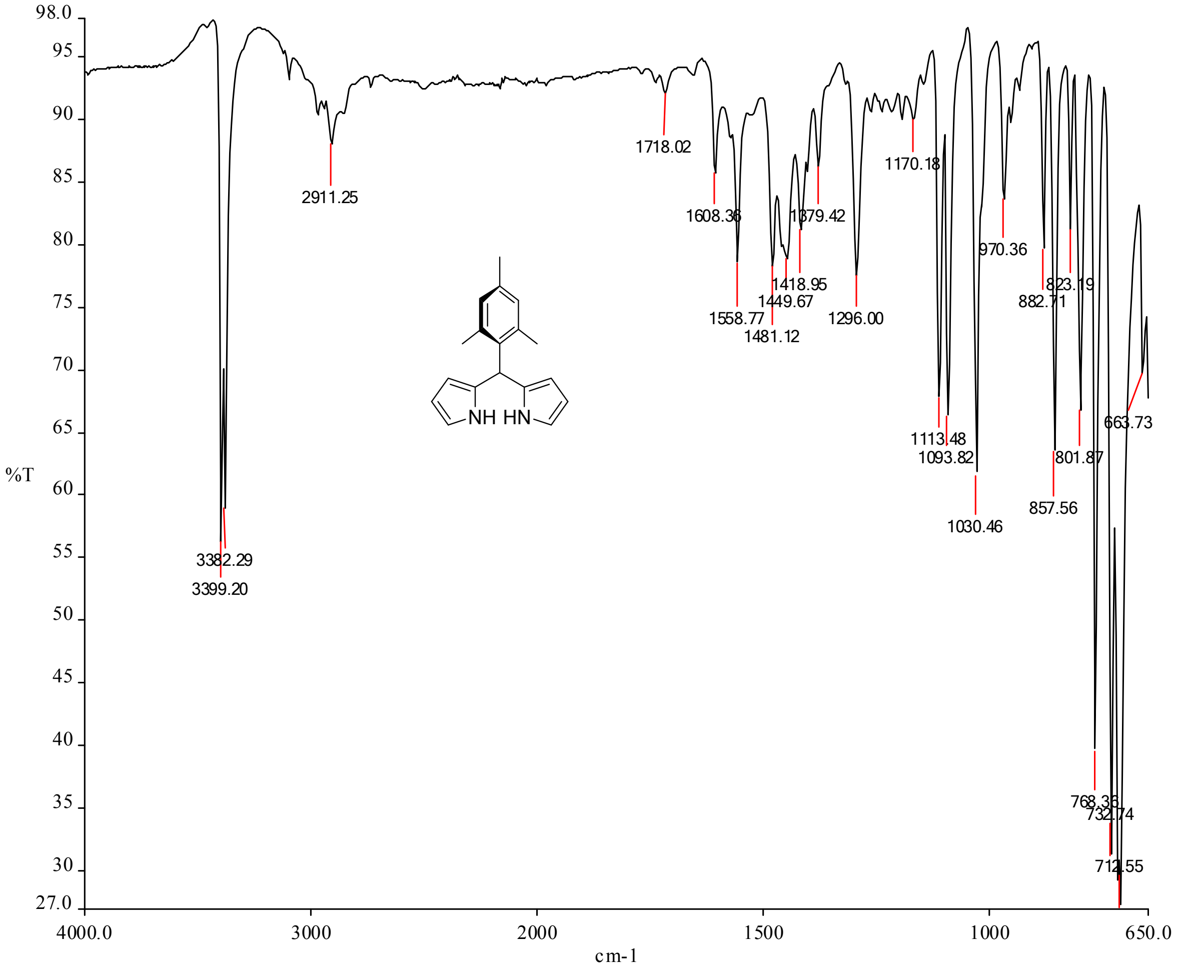

Figure 31S. IR (neat) spectrum of **2,2′-(mesitylmethylene)bis(1H-pyrrole)**.

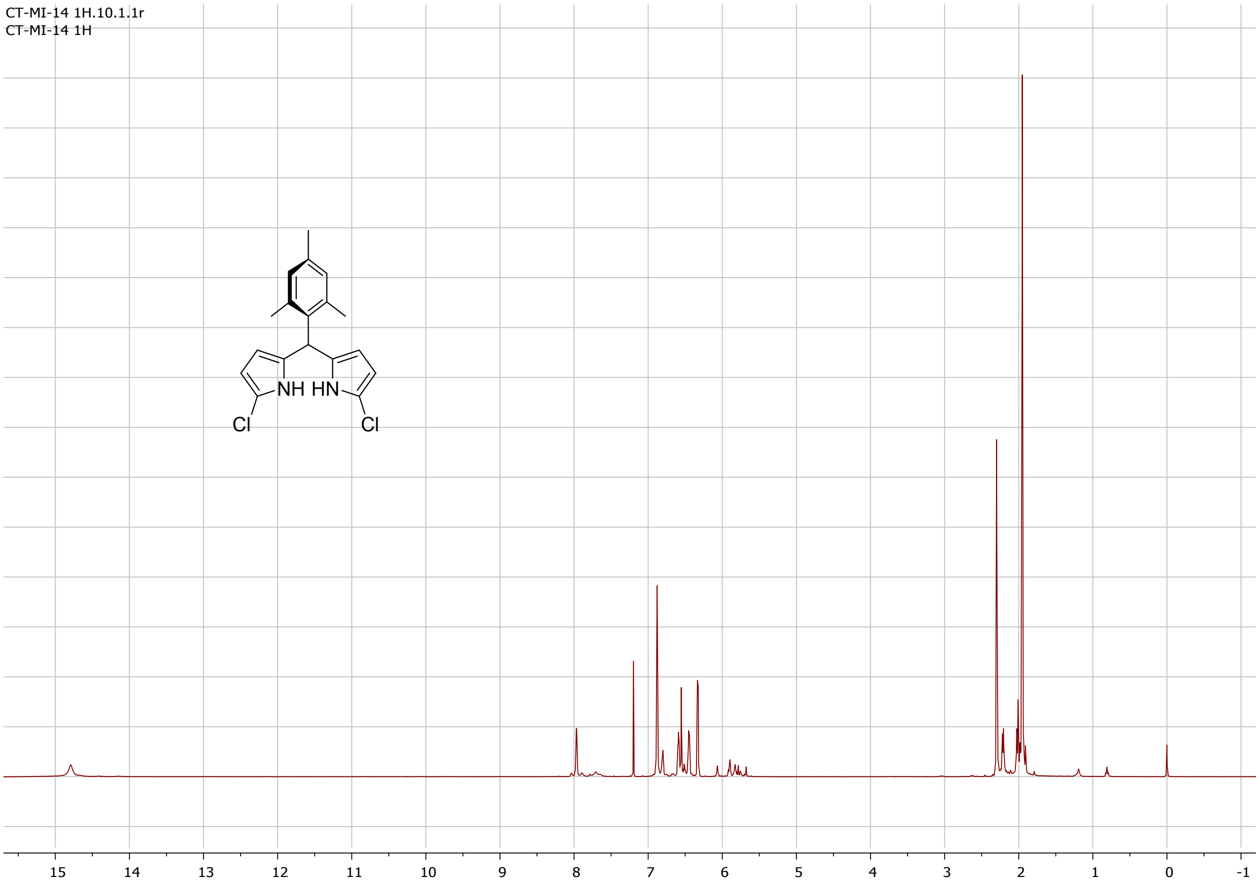

Figure 32S. ^{1}H NMR spectrum (400 MHz) of **5,5′-(mesitylmethylene)bis(2-chloro-1*H*-pyrrole)** in $CDCl_3$.

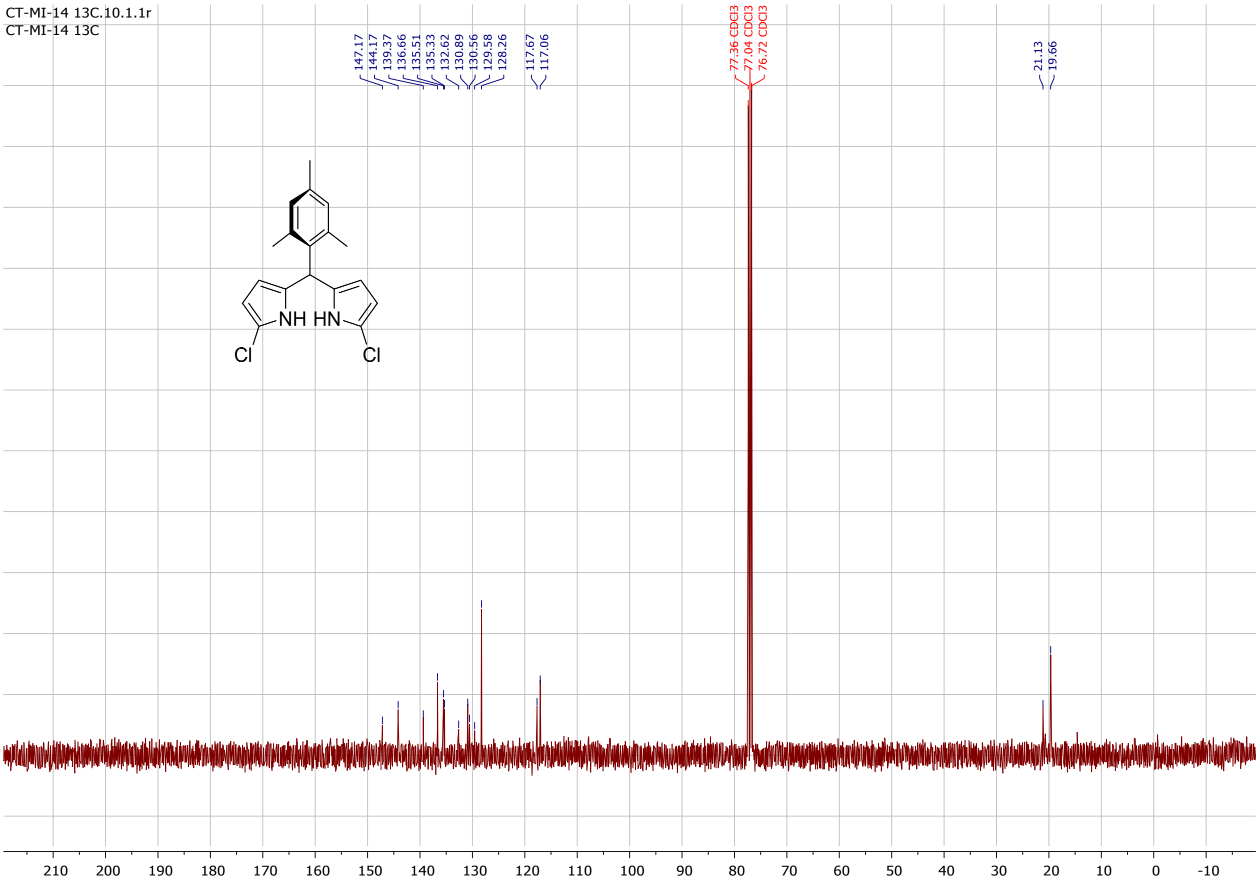

Figure 33S. $^{13}C\{^{1}H\}$ NMR spectrum (101 MHz) of **5,5′-(mesitylmethylene)bis(2-chloro-1*H*-pyrrole)** in $CDCl_3$.

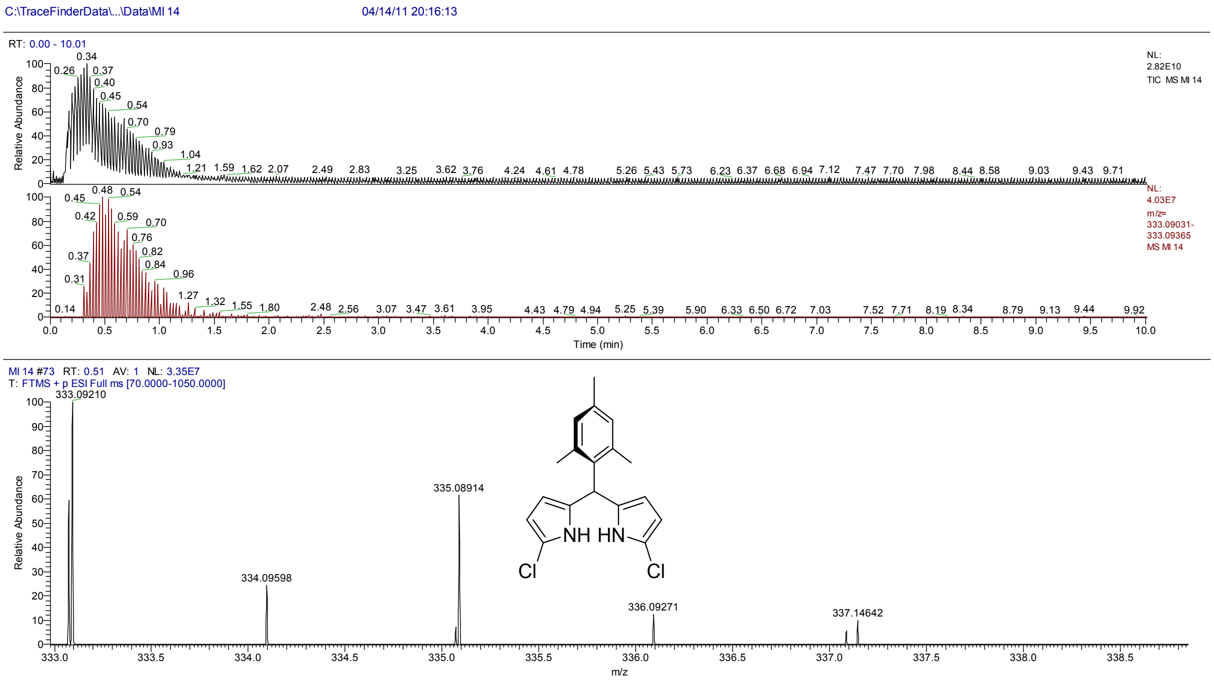

Figure 34S. HRMS (ESI positive) spectrum of **5,5′-(mesitylmethylene)bis(2-chloro-1*H*-pyrrole)**.

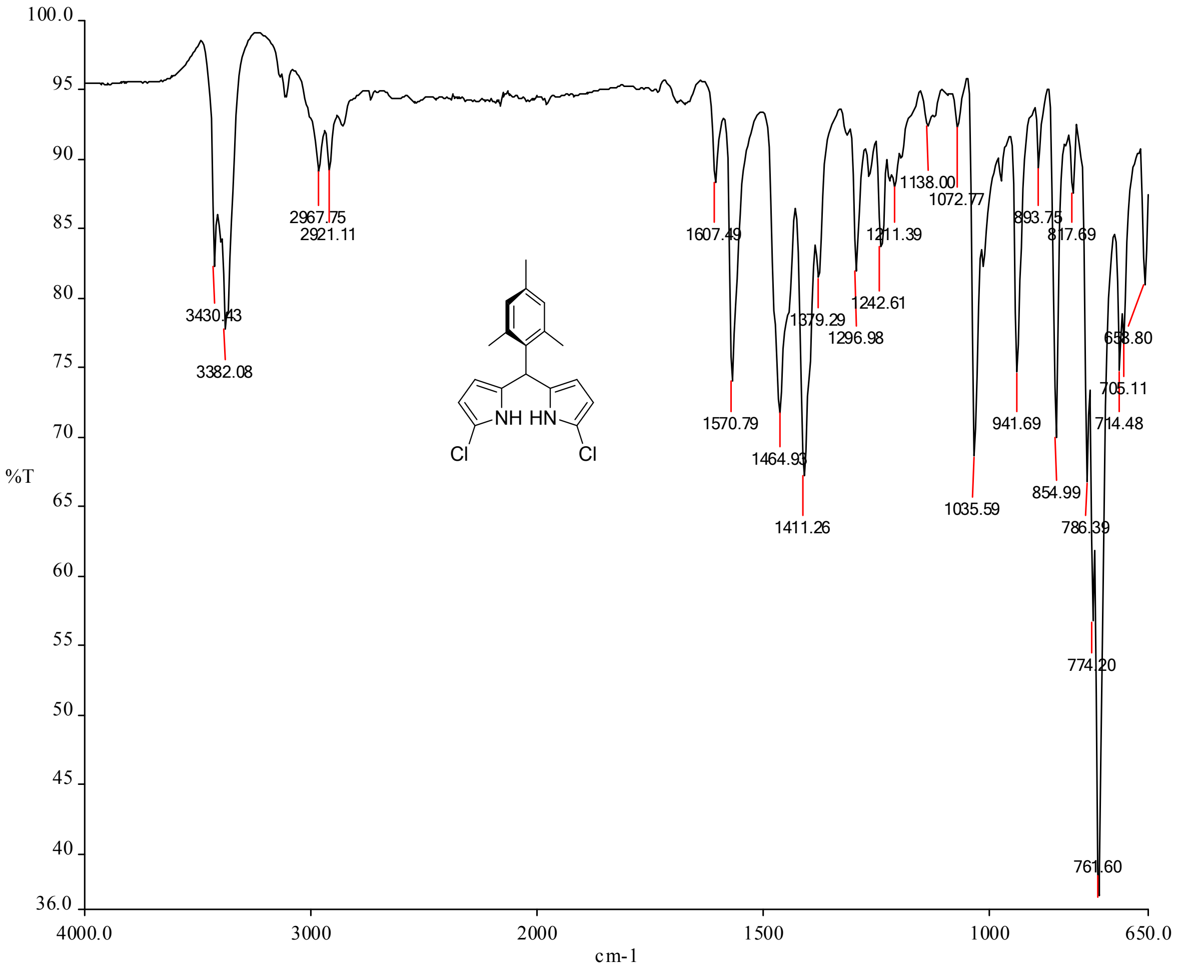

Figure 35S. IR (neat) spectrum of **5,5′-(mesitylmethylene)bis(2-chloro-1*H*-pyrrole)**.

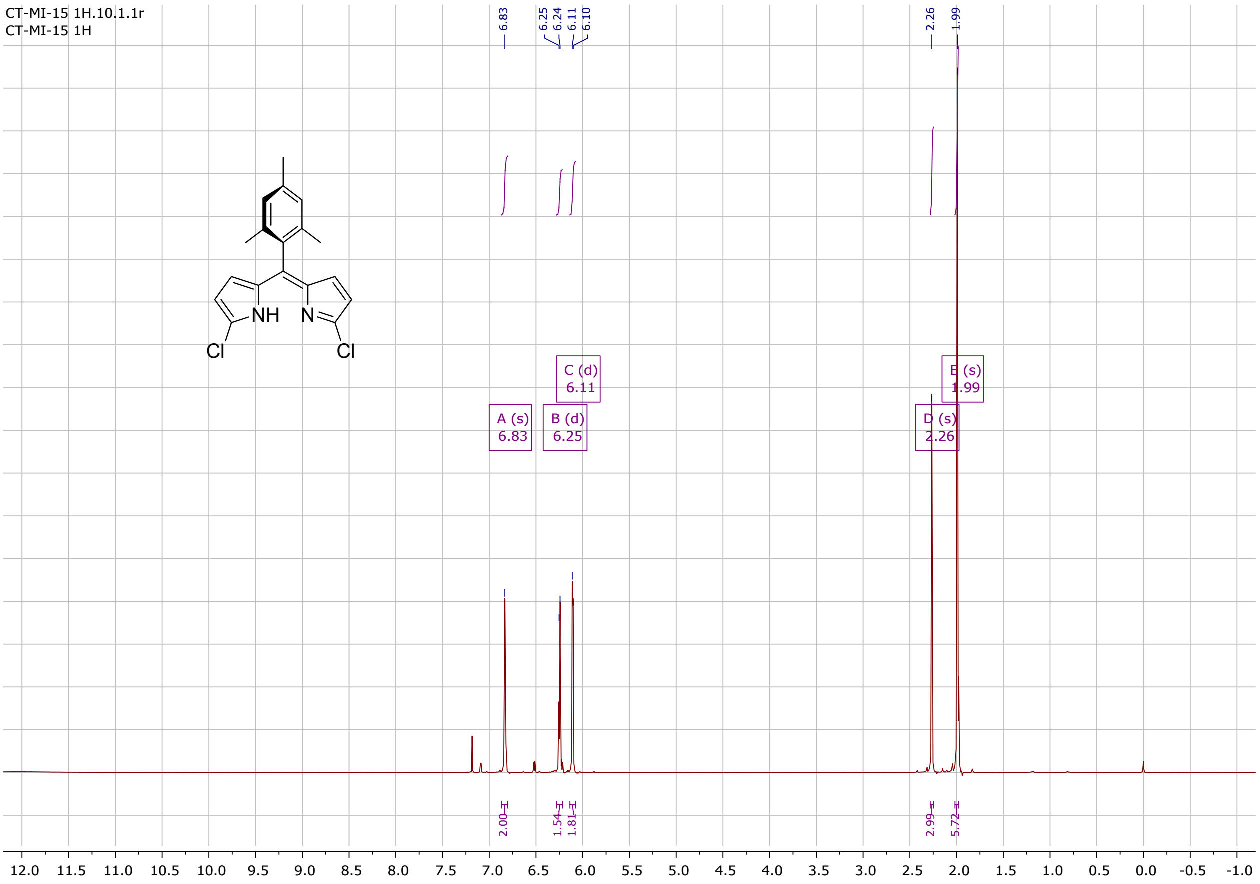

Figure 36S. ^{1}H NMR spectrum (400 MHz) of **(Z)-2-chloro-5-((5-chloro-2*H*-pyrrol-2-ylidene)(mesityl)methyl)- 1*H*-pyrrole** in $CDCl_3$.

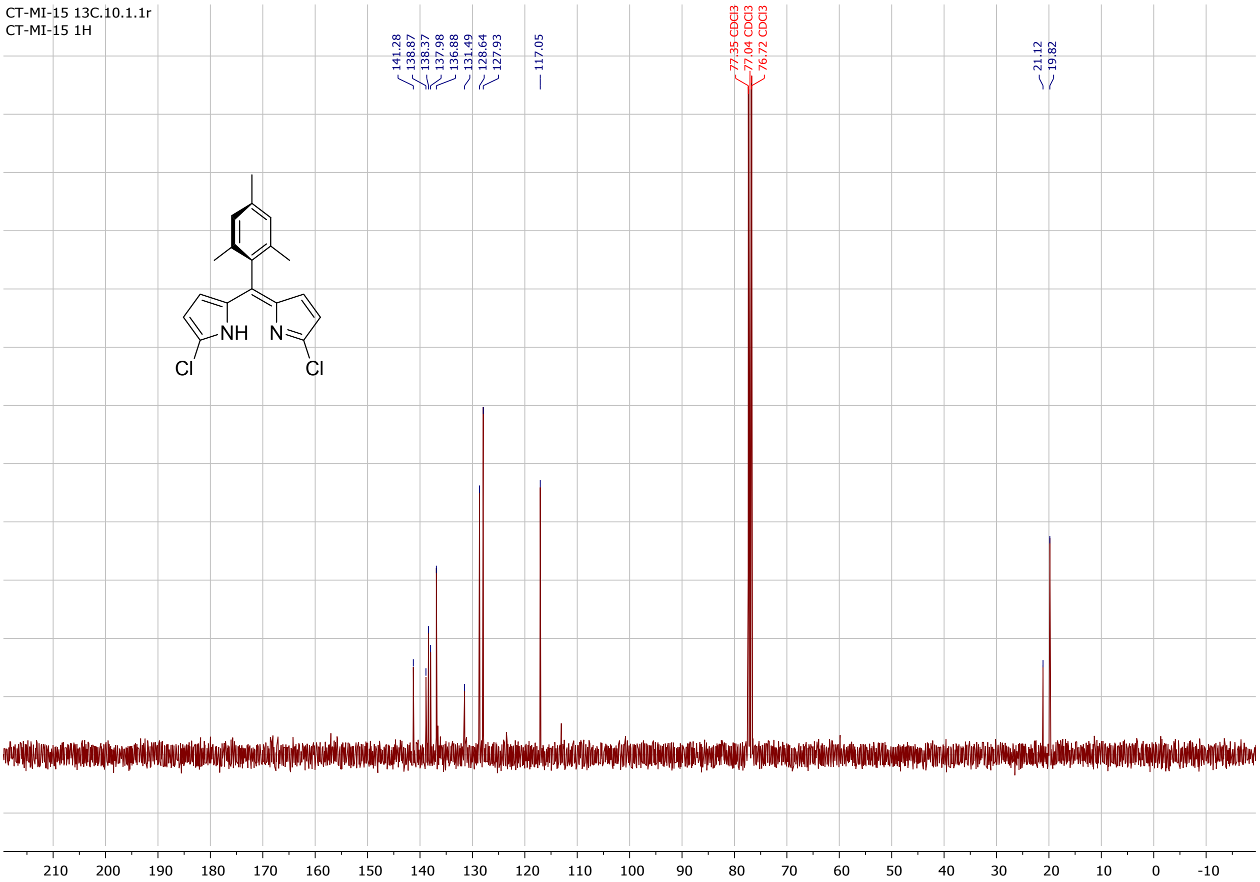

Figure 37S. $^{13}C\{^{1}H\}$ NMR spectrum (101 MHz) of **(Z)-2-chloro-5-((5-chloro-2*H*-pyrrol-2-ylidene)(mesityl)methyl)- 1*H*-pyrrole** in $CDCl_3$.

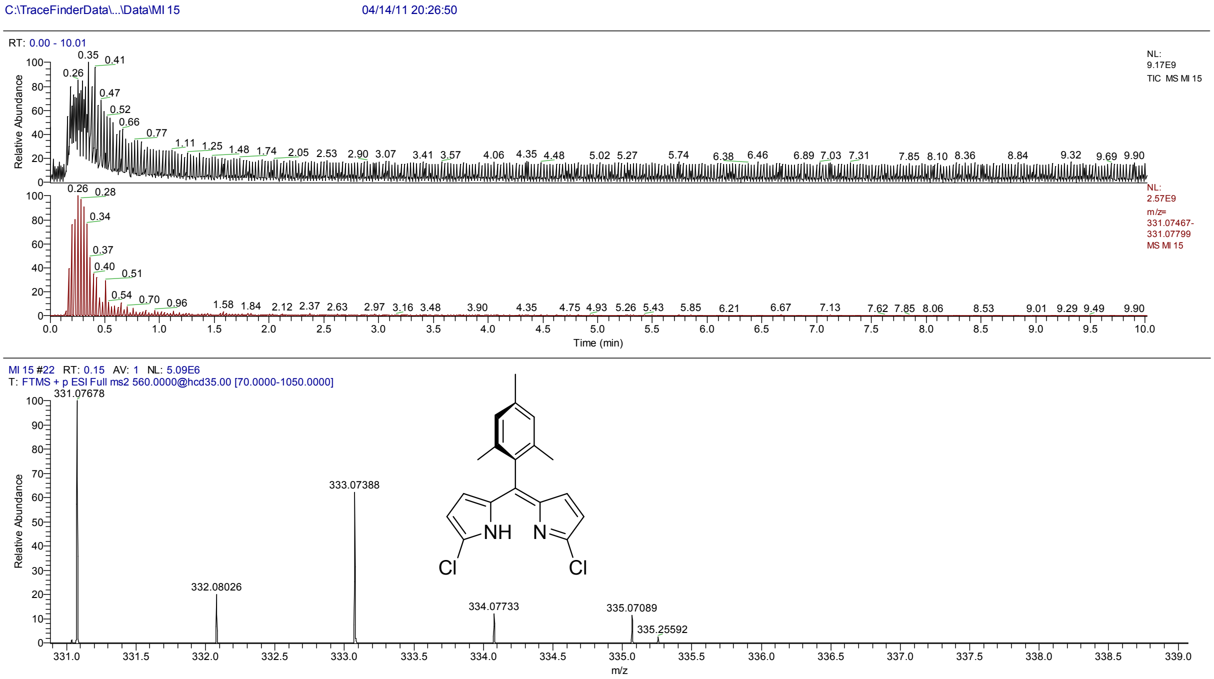

Figure 38S. HRMS (ESI positive) spectrum of **(Z)-2-chloro-5-((5-chloro-2*H*-pyrrol-2-ylidene)(mesityl)methyl)- 1*H*-pyrrole**.

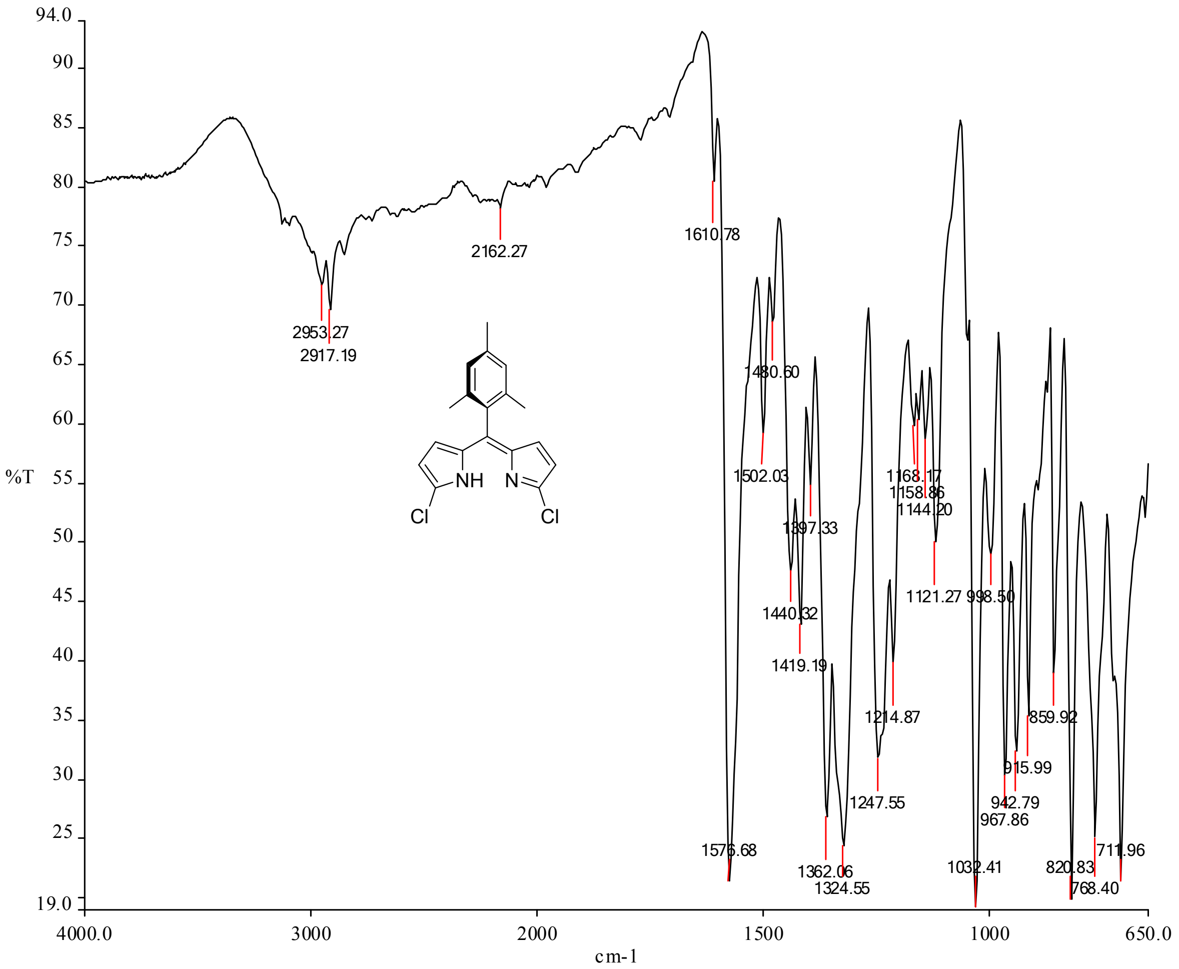

Figure 39S. IR (neat) spectrum of **(Z)-2-chloro-5-((5-chloro-2*H*-pyrrol-2-ylidene)(mesityl)methyl)- 1*H*-pyrrole**.

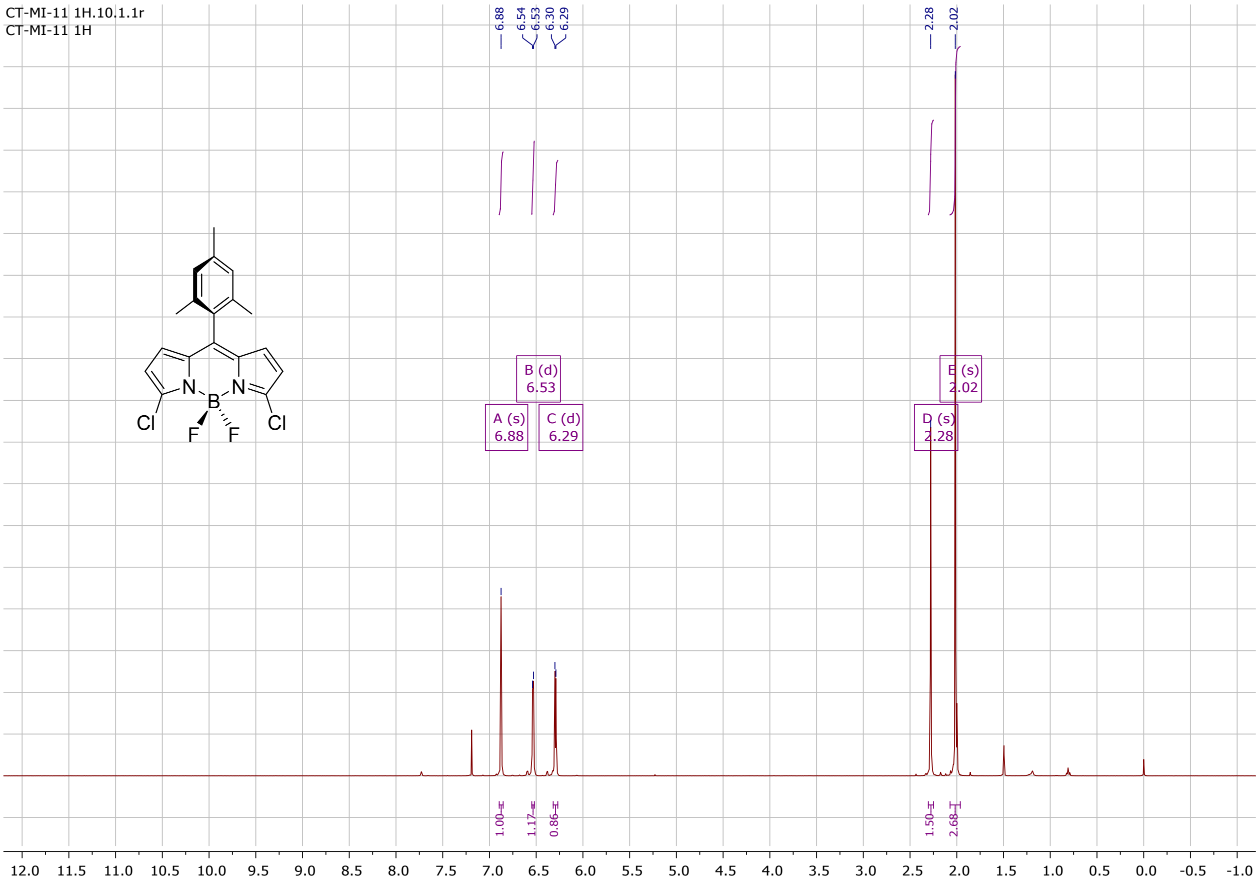

Figure 40S. ^{1}H NMR spectrum (400 MHz) of **2a** in $CDCl_3$.

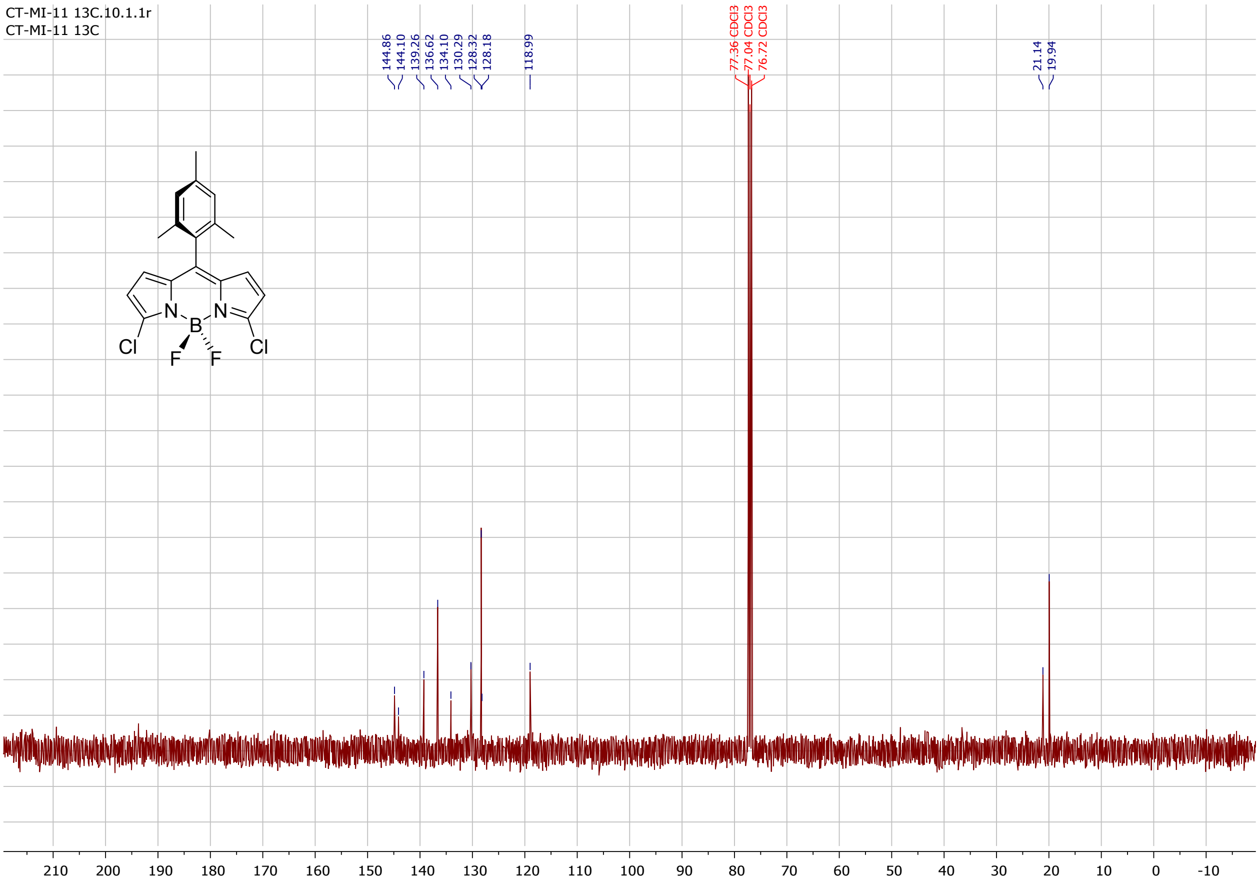

Figure 41S. $^{13}C\{^1H\}$ NMR spectrum (101 MHz) of **2a** in $CDCl_3$.

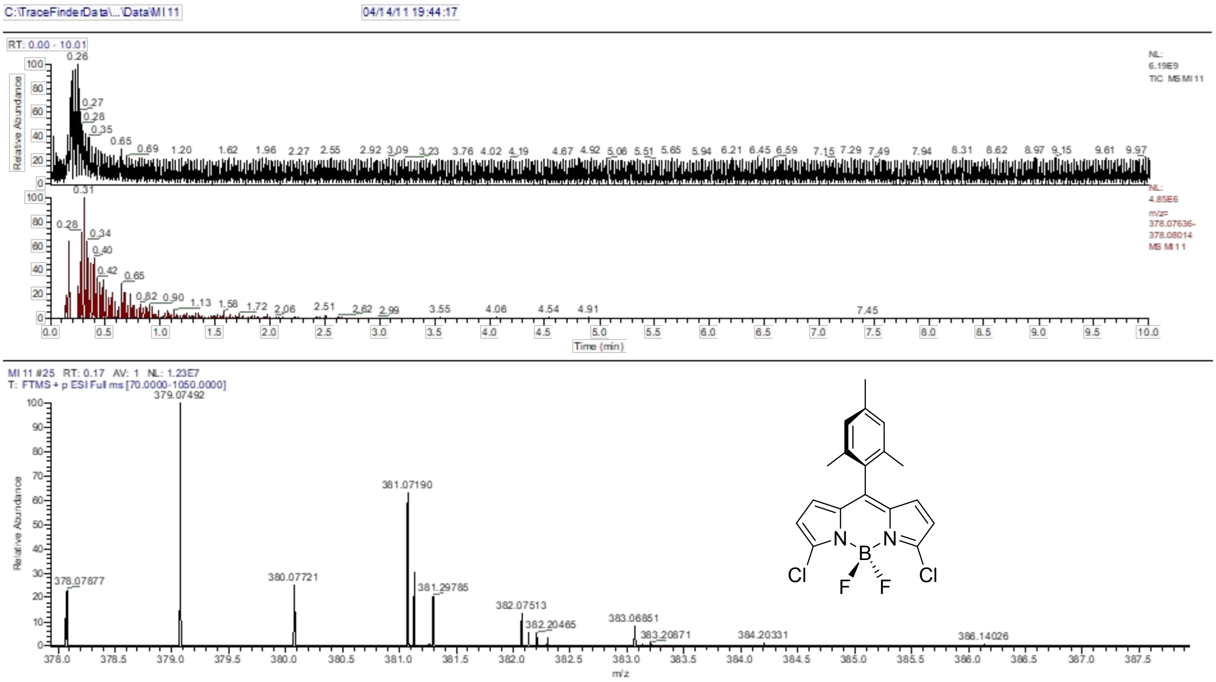

Figure 42S. HRMS (ESI positive) spectrum of **2a**.

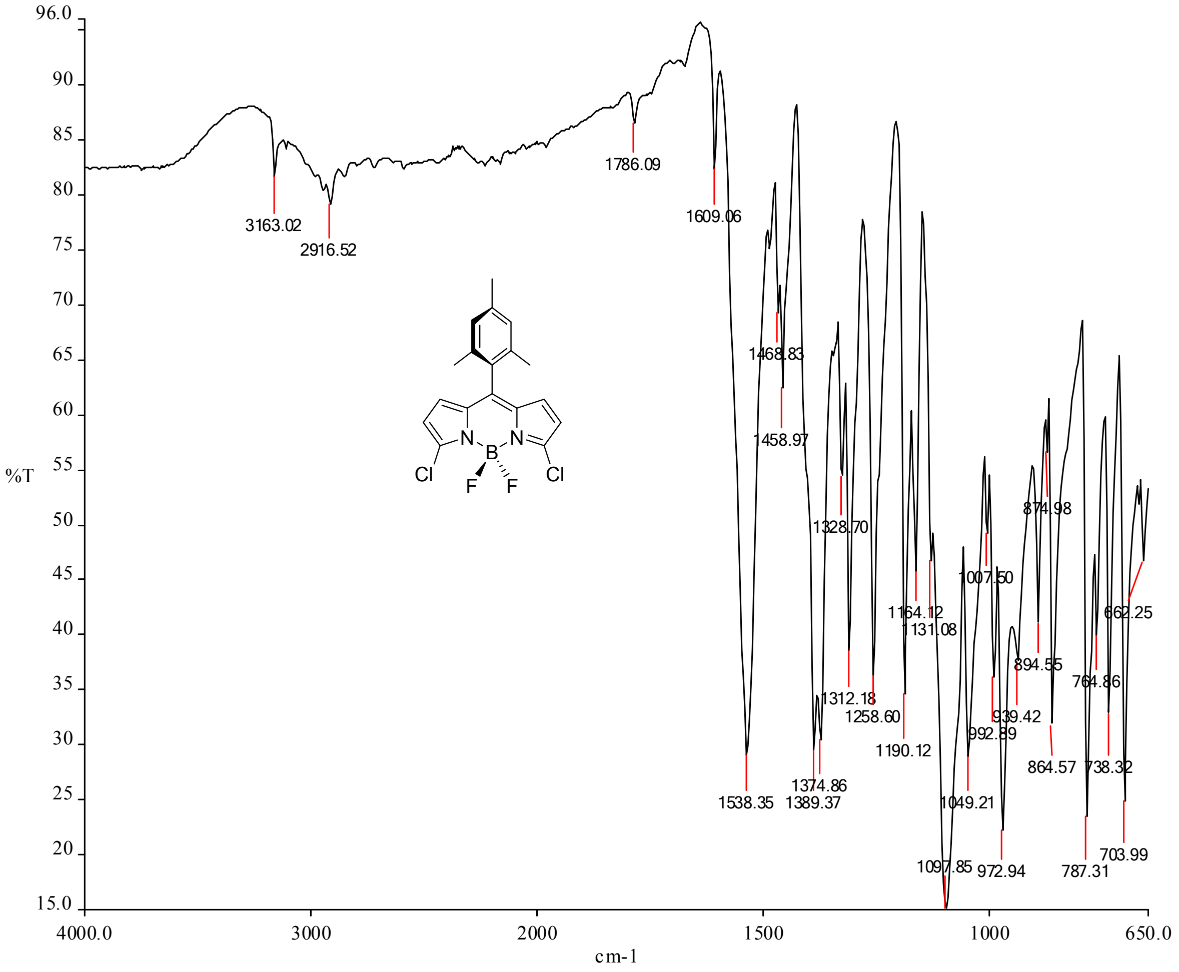

Figure 43S. IR (neat) spectrum of **2a**.

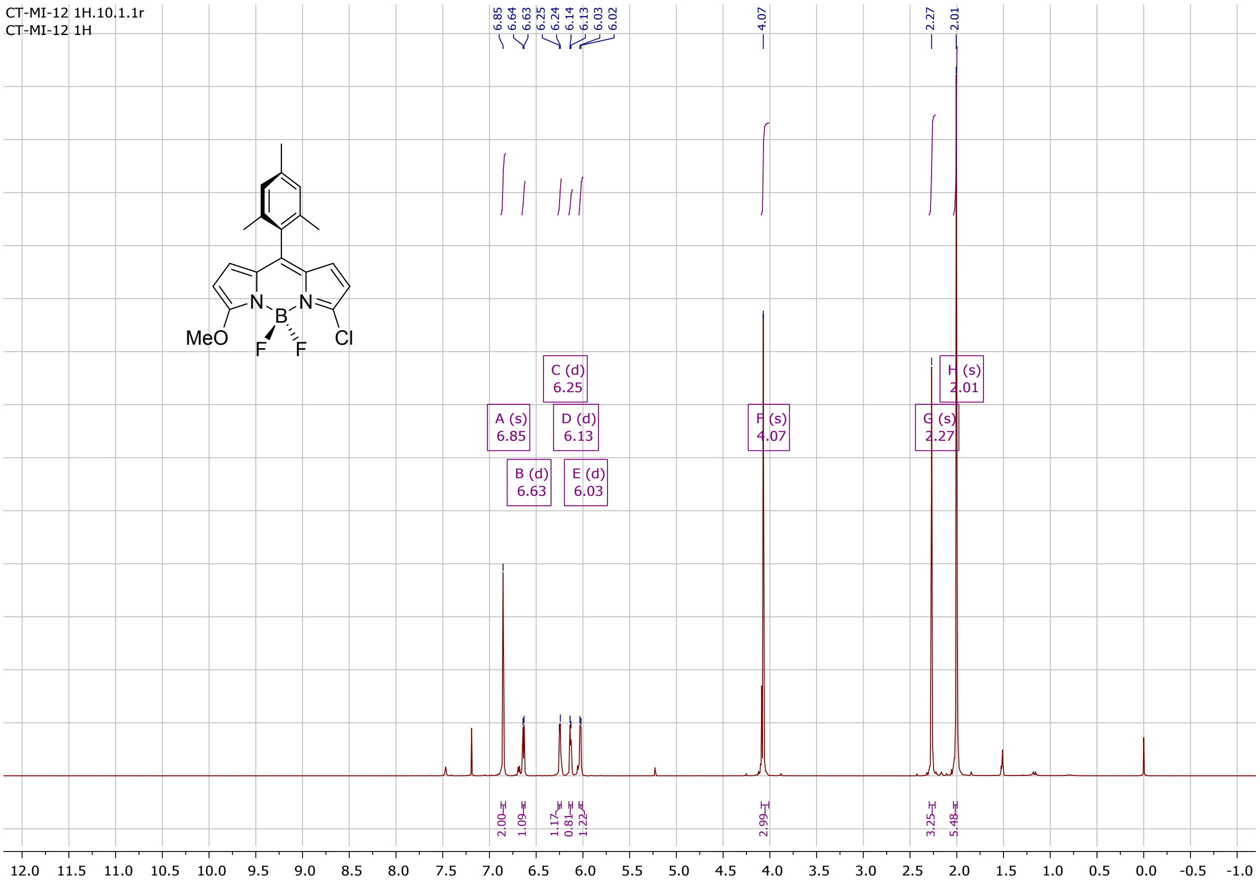

Figure 44S. ^{1}H NMR spectrum (400 MHz) of **2b** in $CDCl_3$.

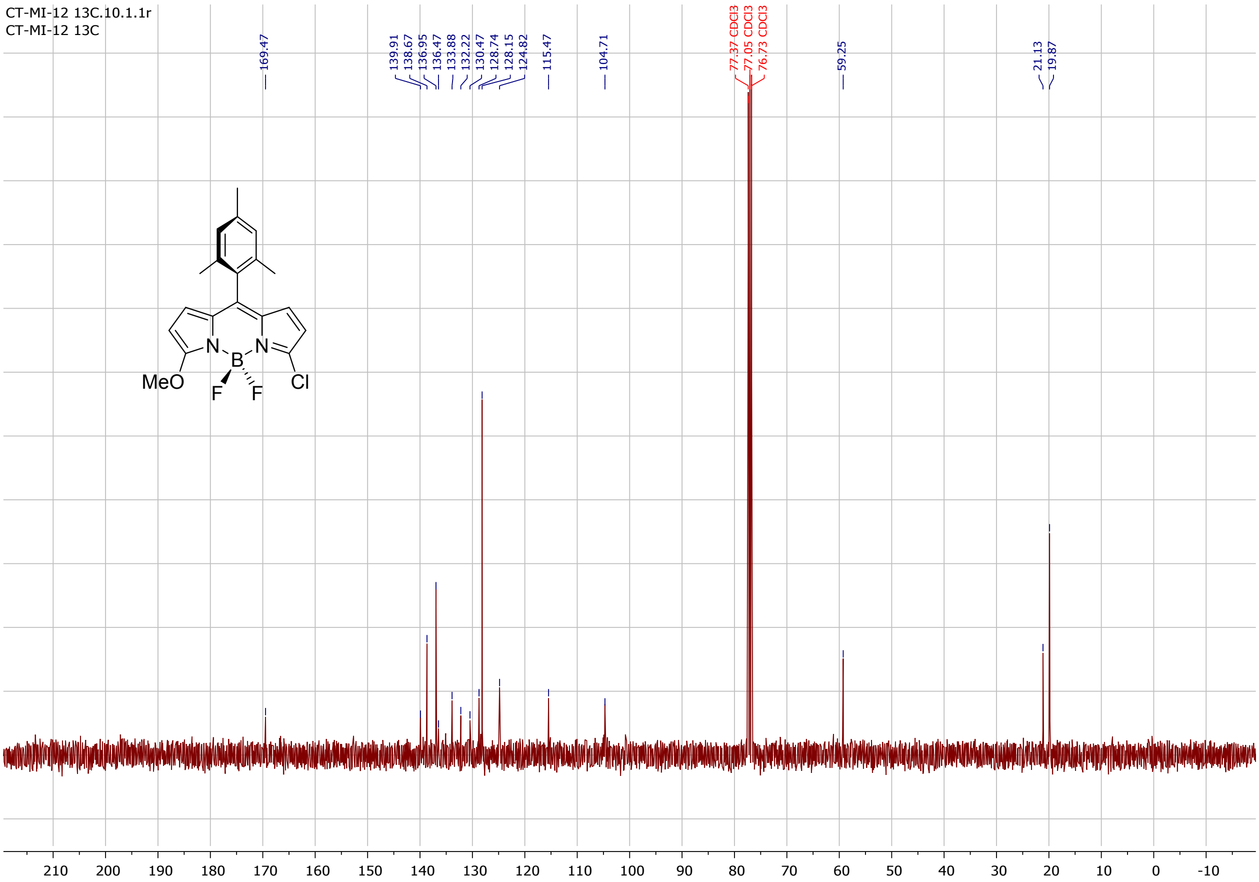

Figure 45S. $^{13}C\{^{1}H\}$ NMR spectrum (101 MHz) of **2b** in $CDCl_3$.

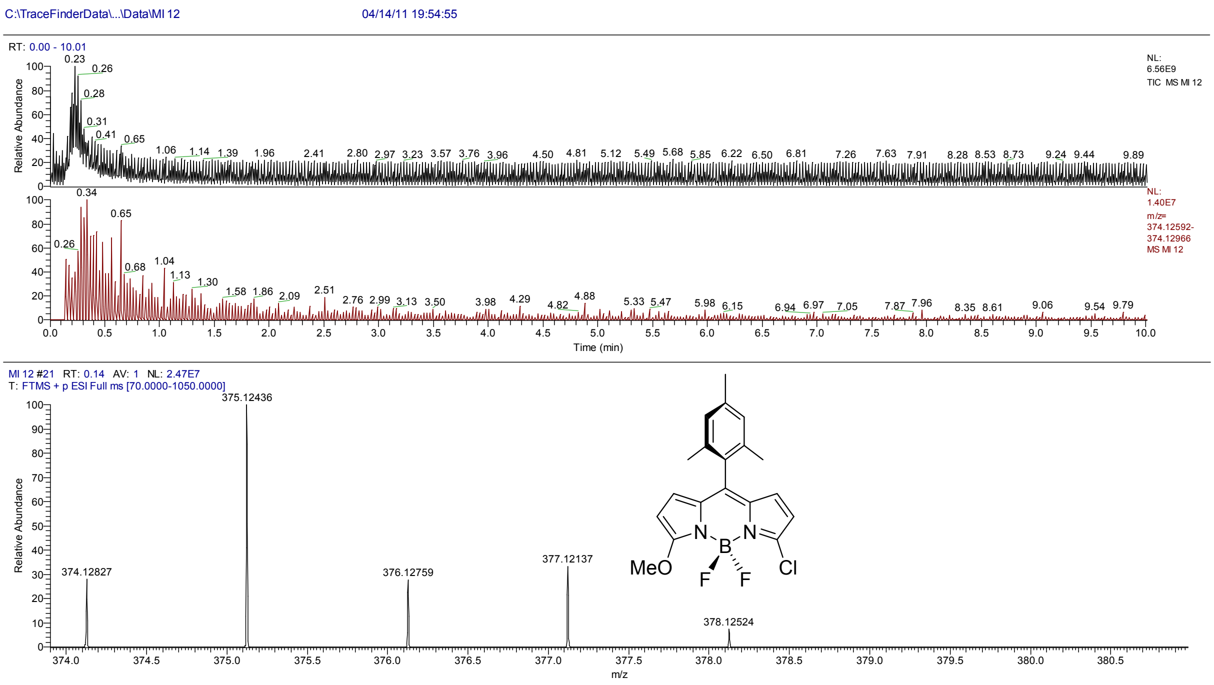

Figure 46S. HRMS (ESI positive) spectrum of **2b**.

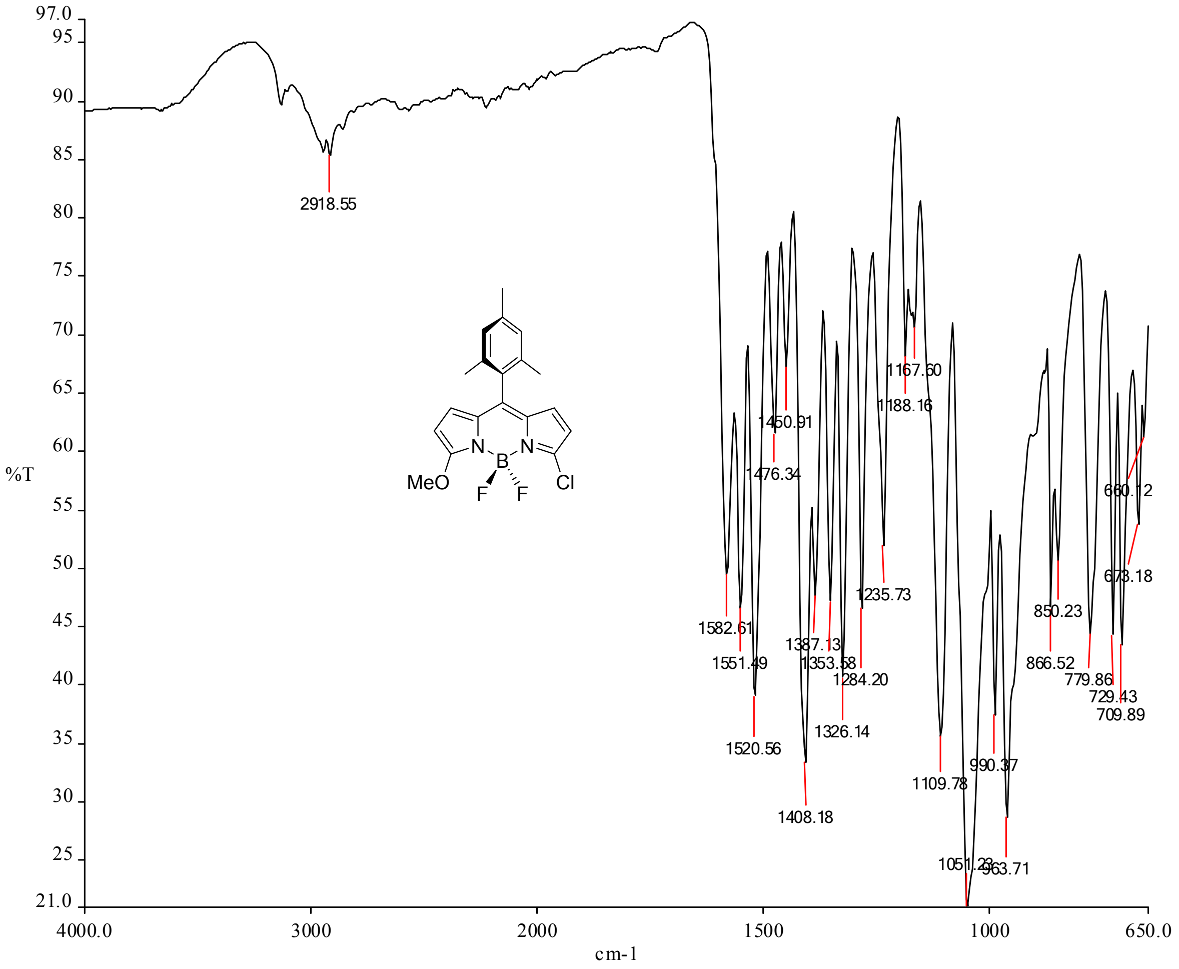

Figure 47S. IR (neat) spectrum of **2b**.

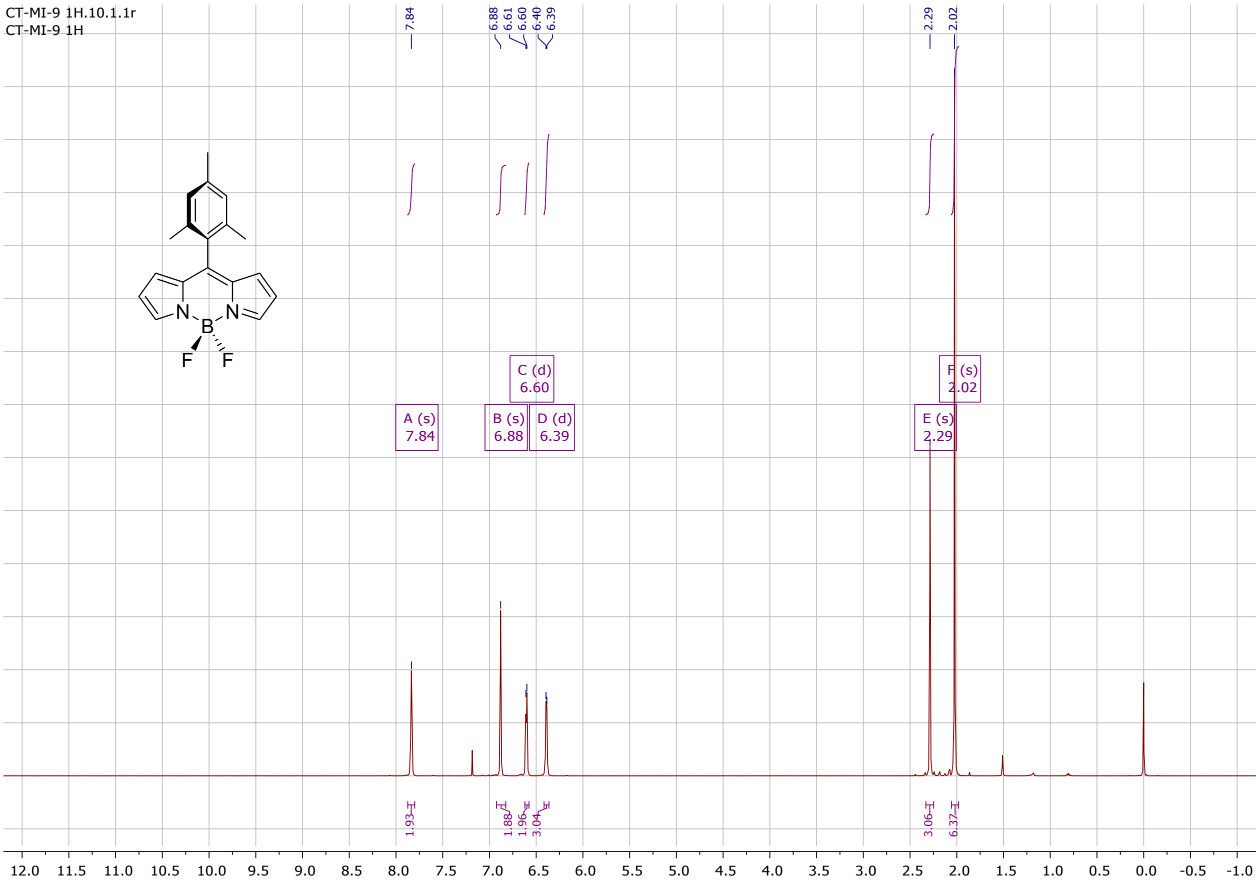

Figure 48S. ^{1}H NMR spectrum (400 MHz) of **8-mesityl-BODIPY** in $CDCl_3$.

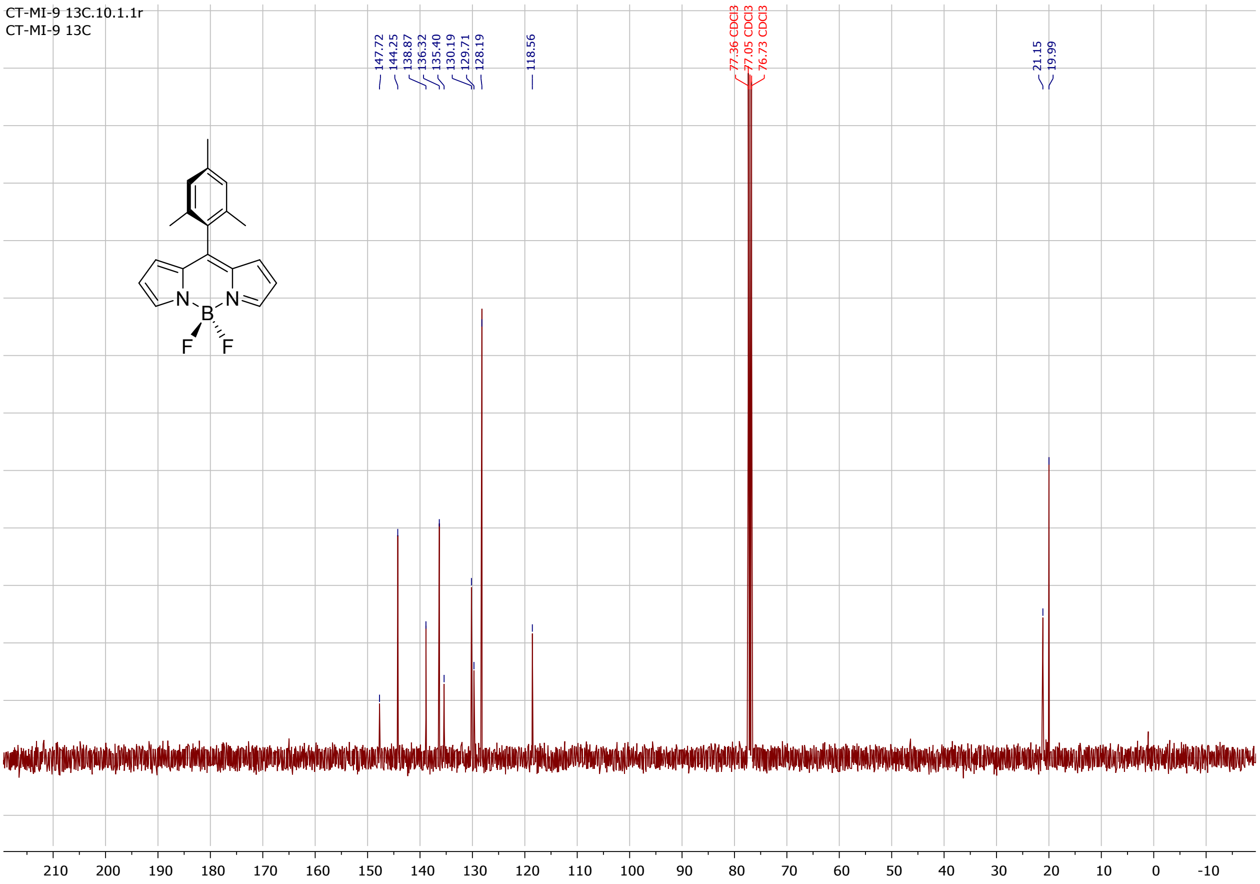

Figure 49S. $^{13}C\{^{1}H\}$ NMR spectrum (101 MHz) of **8-mesityl-BODIPY** in $CDCl_3$.

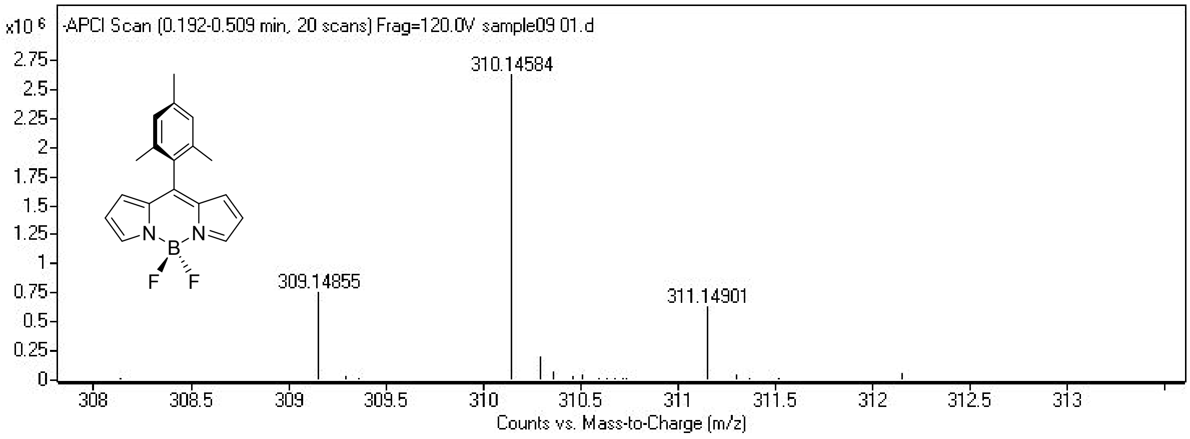

Figure 50S. HRMS (APCI negative) spectrum of **8-mesityl-BODIPY**.

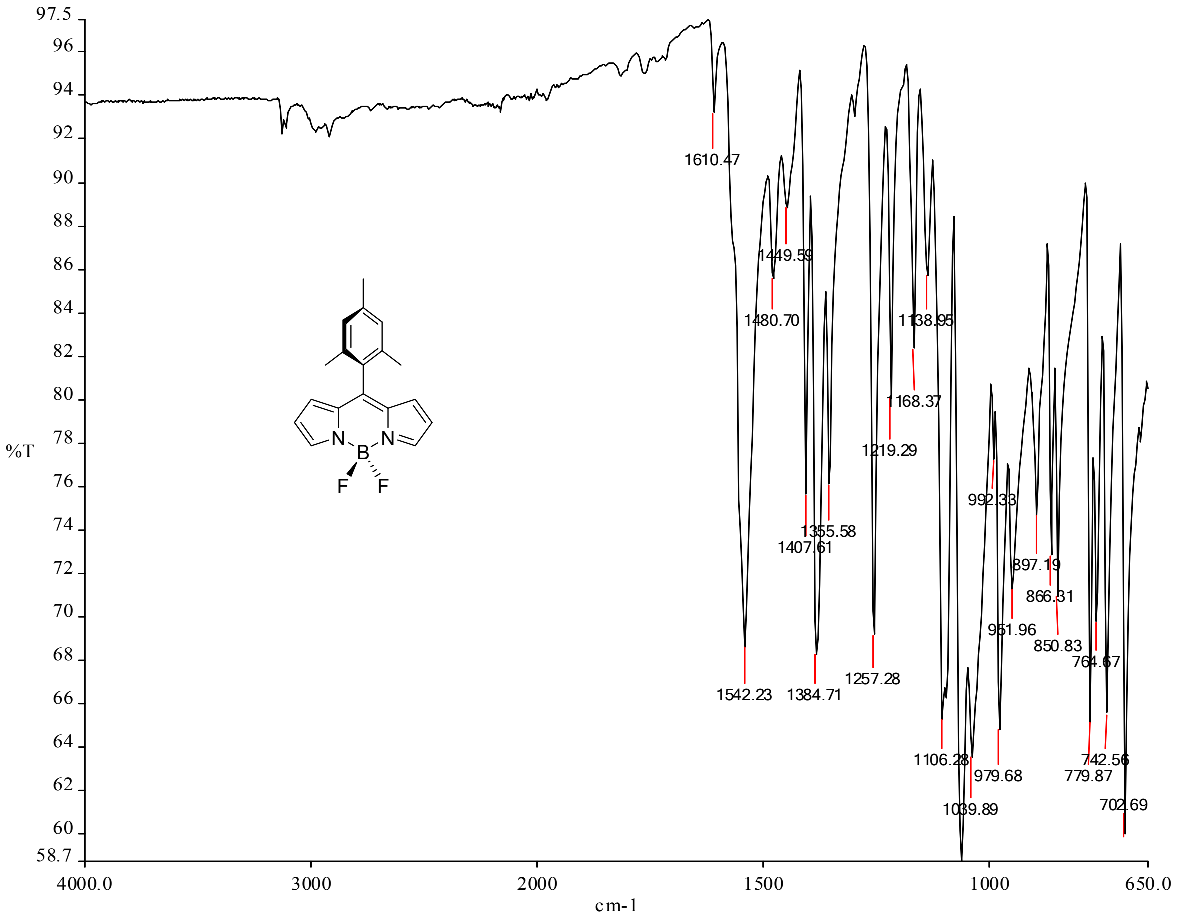

Figure 51S. IR (neat) spectrum of **8-mesityl-BODIPY**.

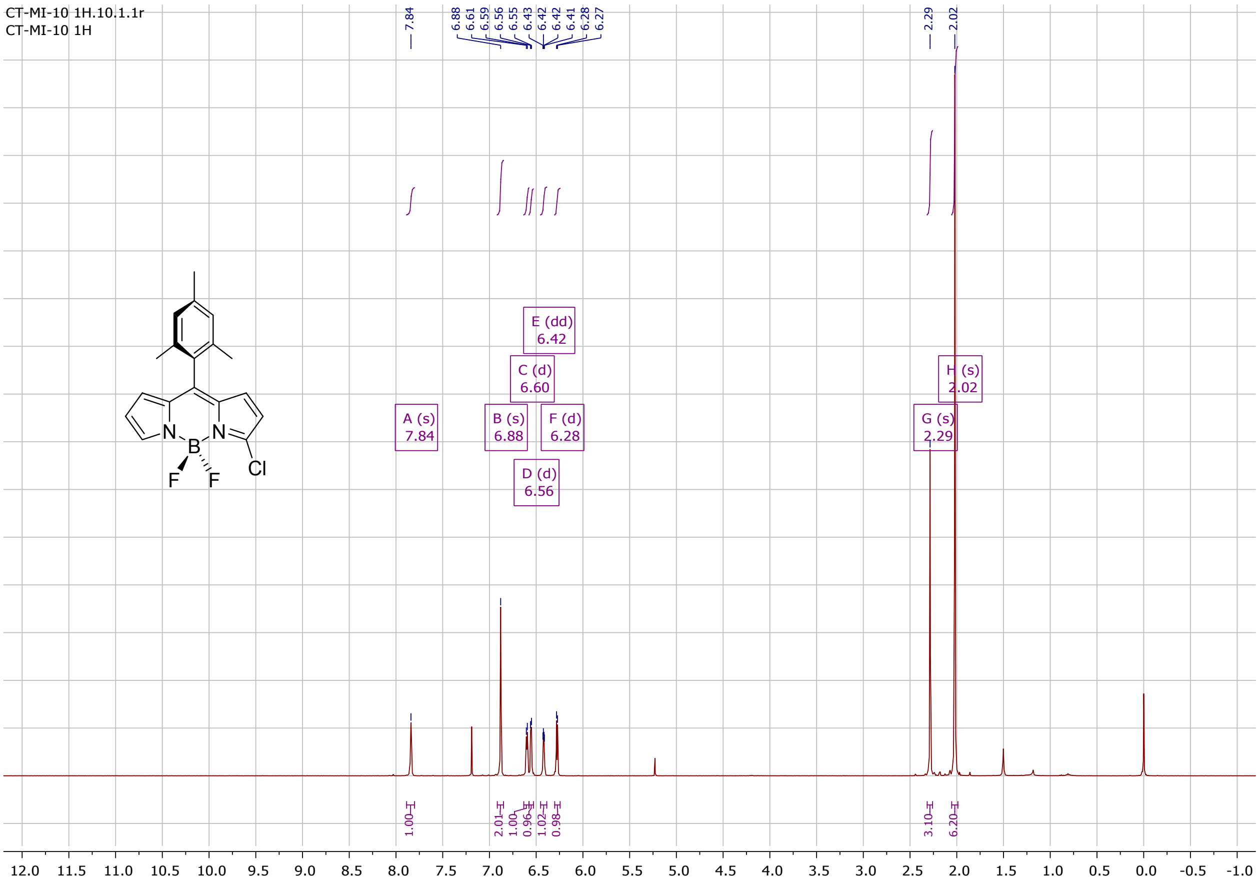

Figure 52S. ^{1}H NMR spectrum (400 MHz) of **2c** in $CDCl_3$.

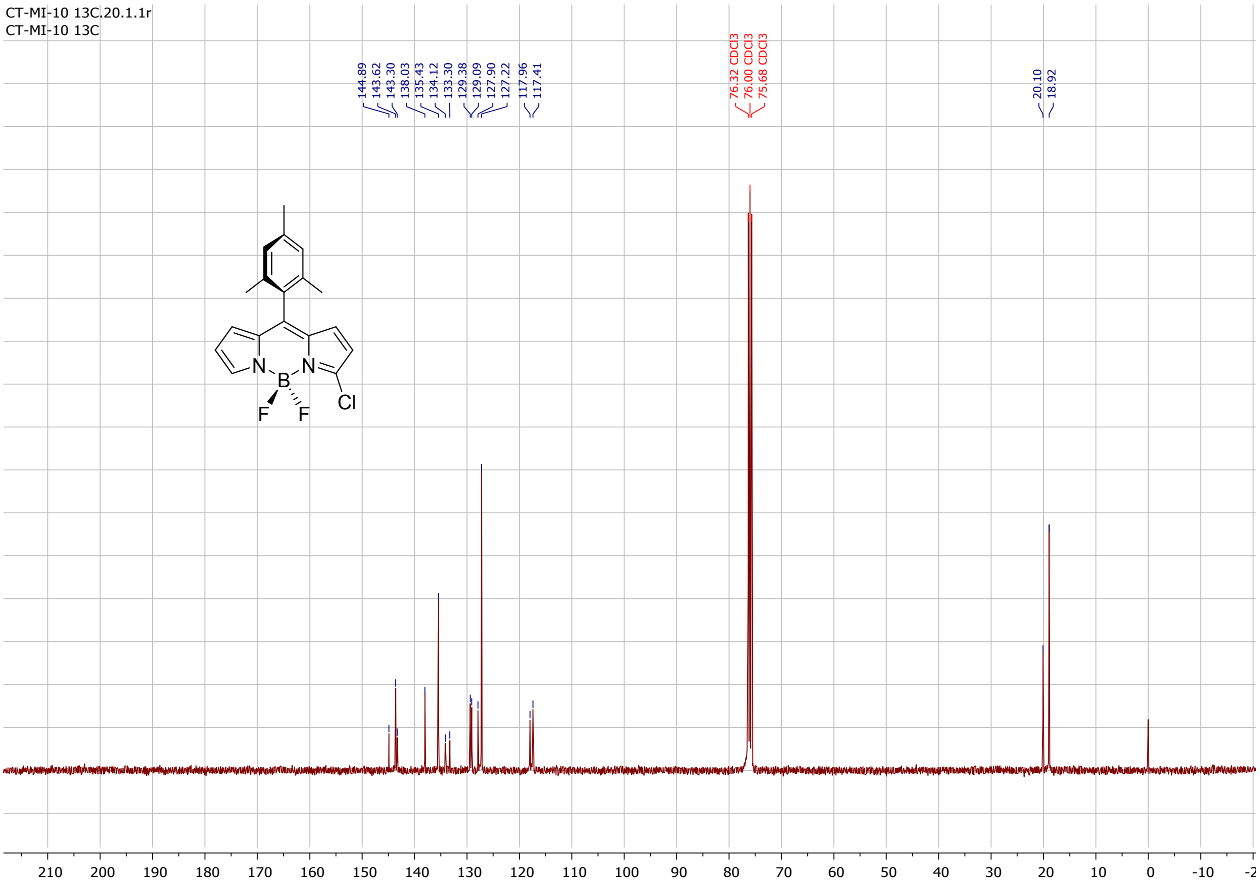

Figure 53S. $^{13}C\{^{1}H\}$ NMR spectrum (101 MHz) of **2c** in $CDCl_3$.

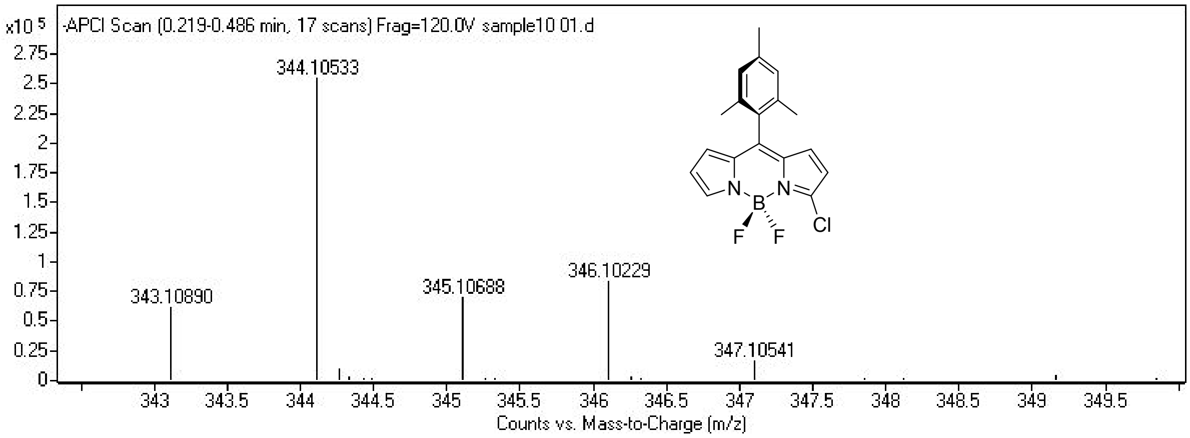

Figure 54S. HRMS (APCI negative) spectrum of **2c**.

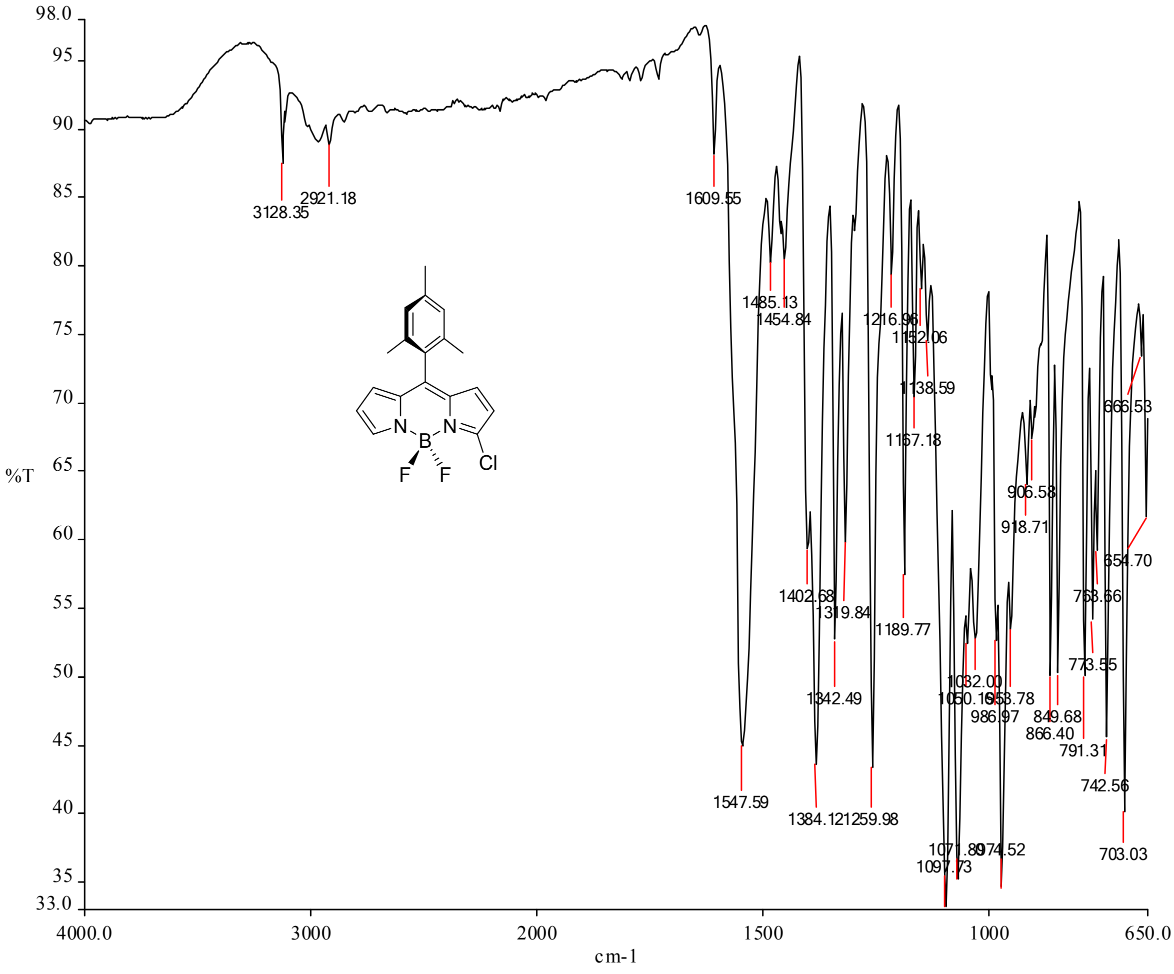

Figure 55S. IR (neat) spectrum of **2c**.

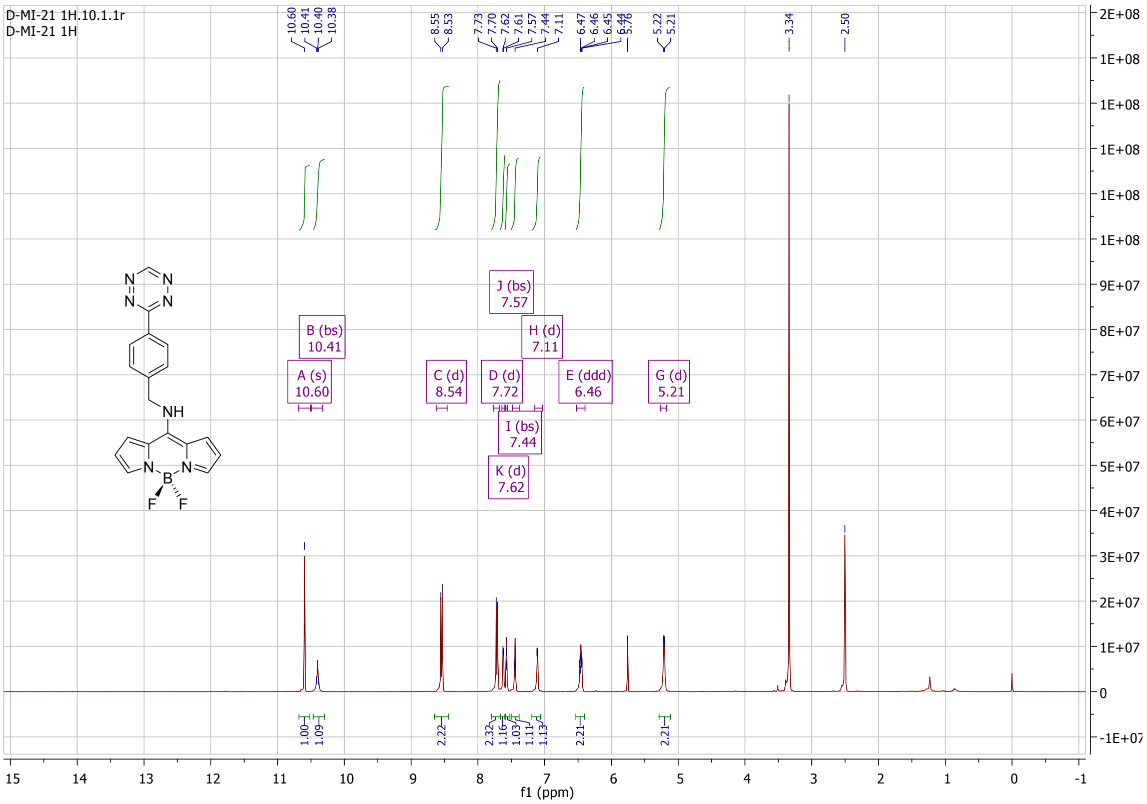

Figure 56S. ^{1}H NMR spectrum (400 MHz) of **1TzH** in DMSO-d_6.

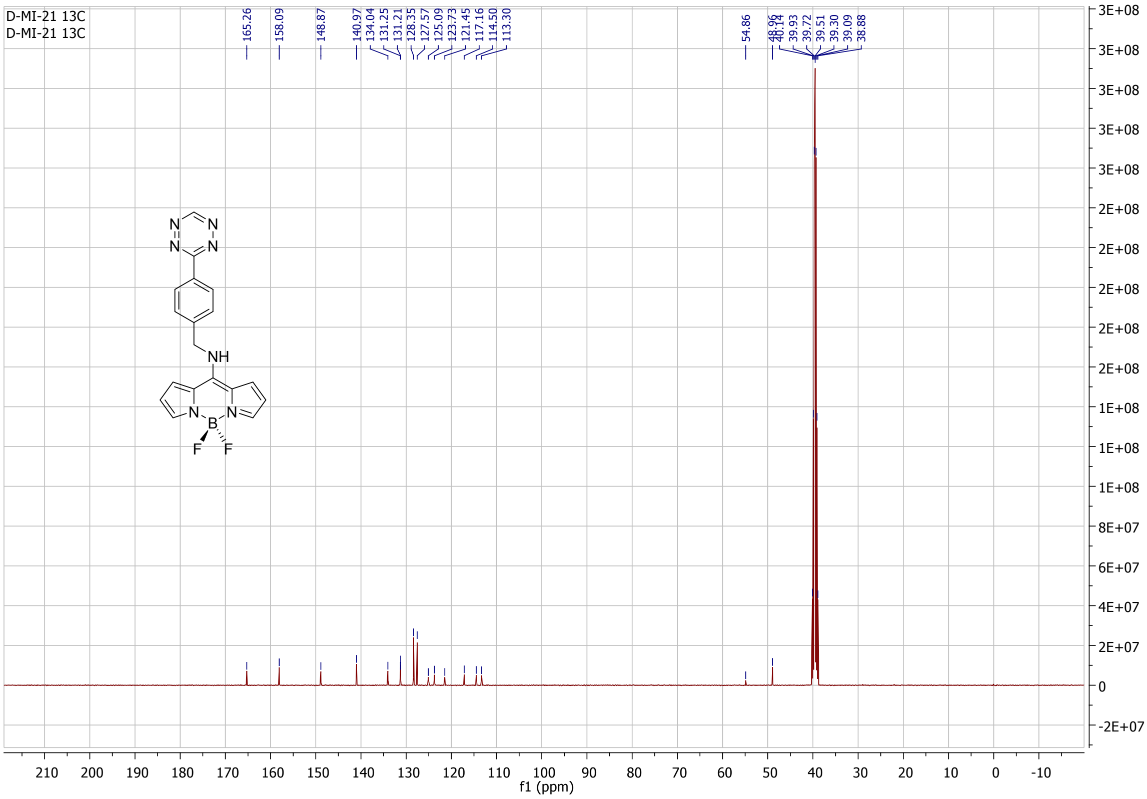

Figure 57S. ^{13}C{^{1}H} NMR spectrum (101 MHz) of **1TzH** in DMSO-d_6.

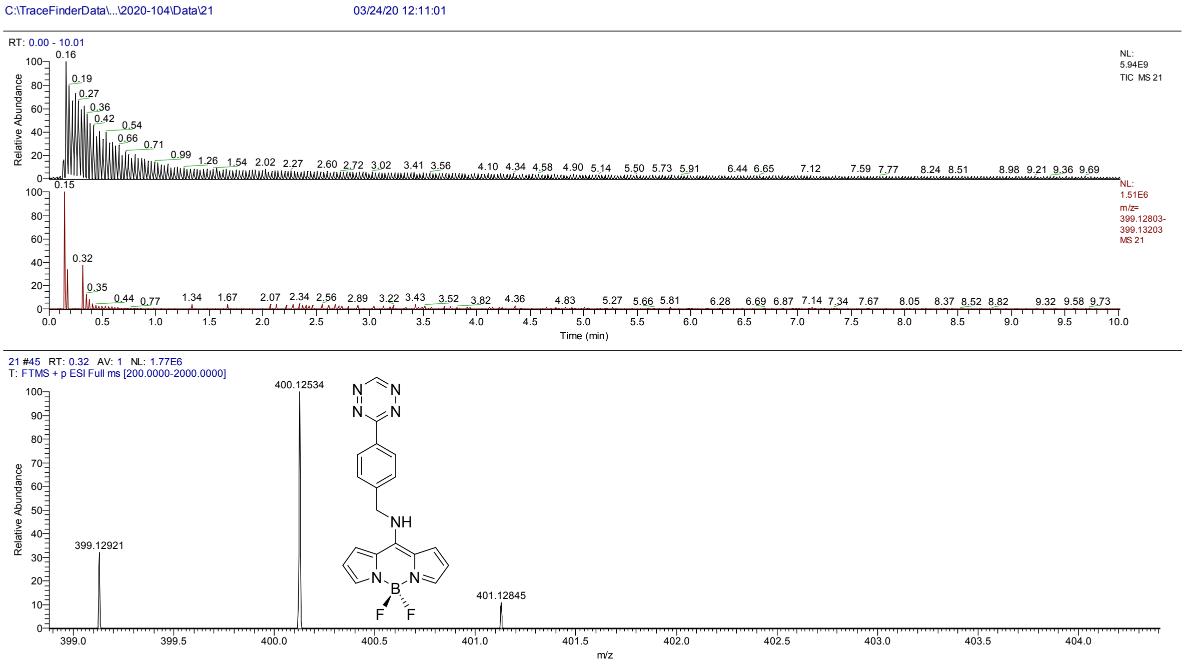

Figure 58S. HRMS (ESI positive) spectrum of **1TzH**.

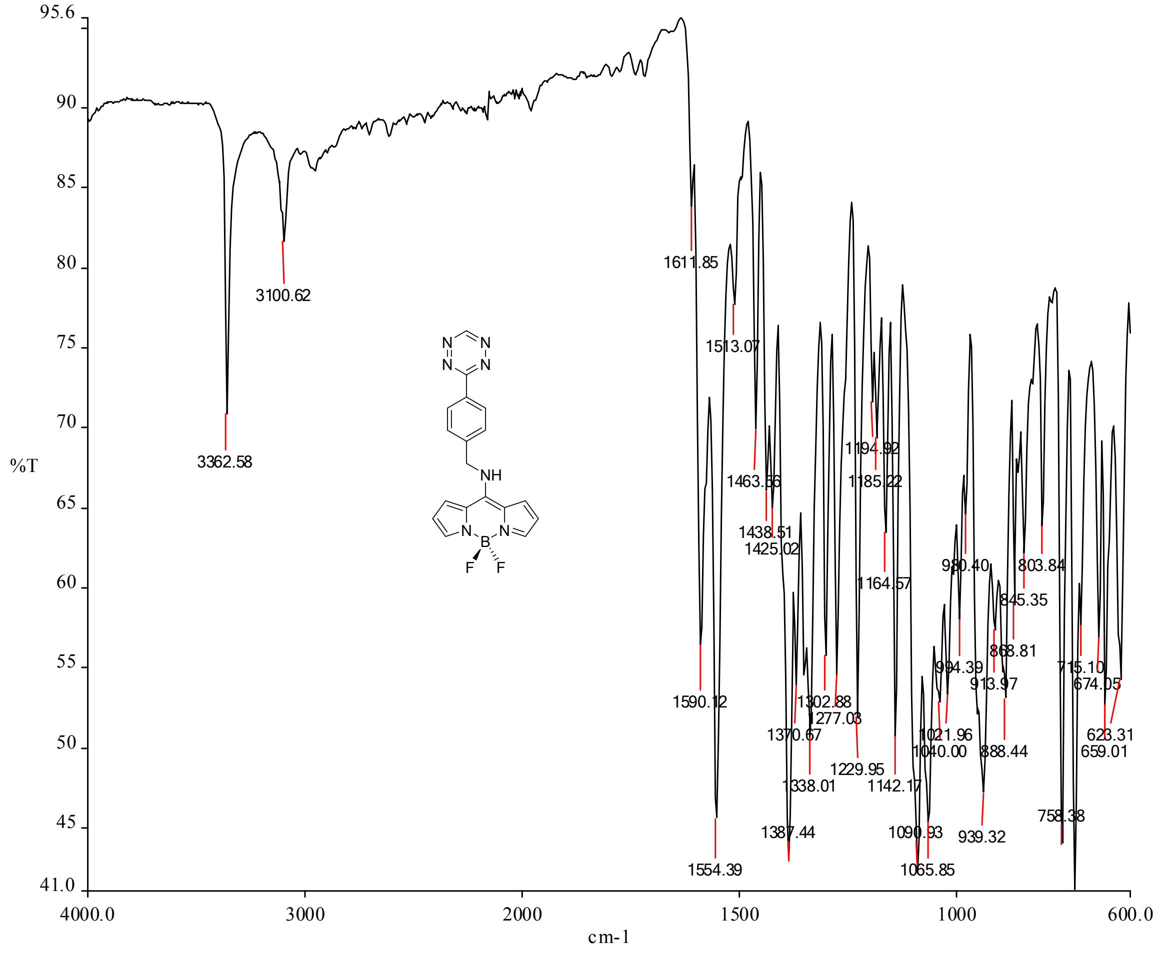

Figure 59S. IR (neat) spectrum of **1TzH**.

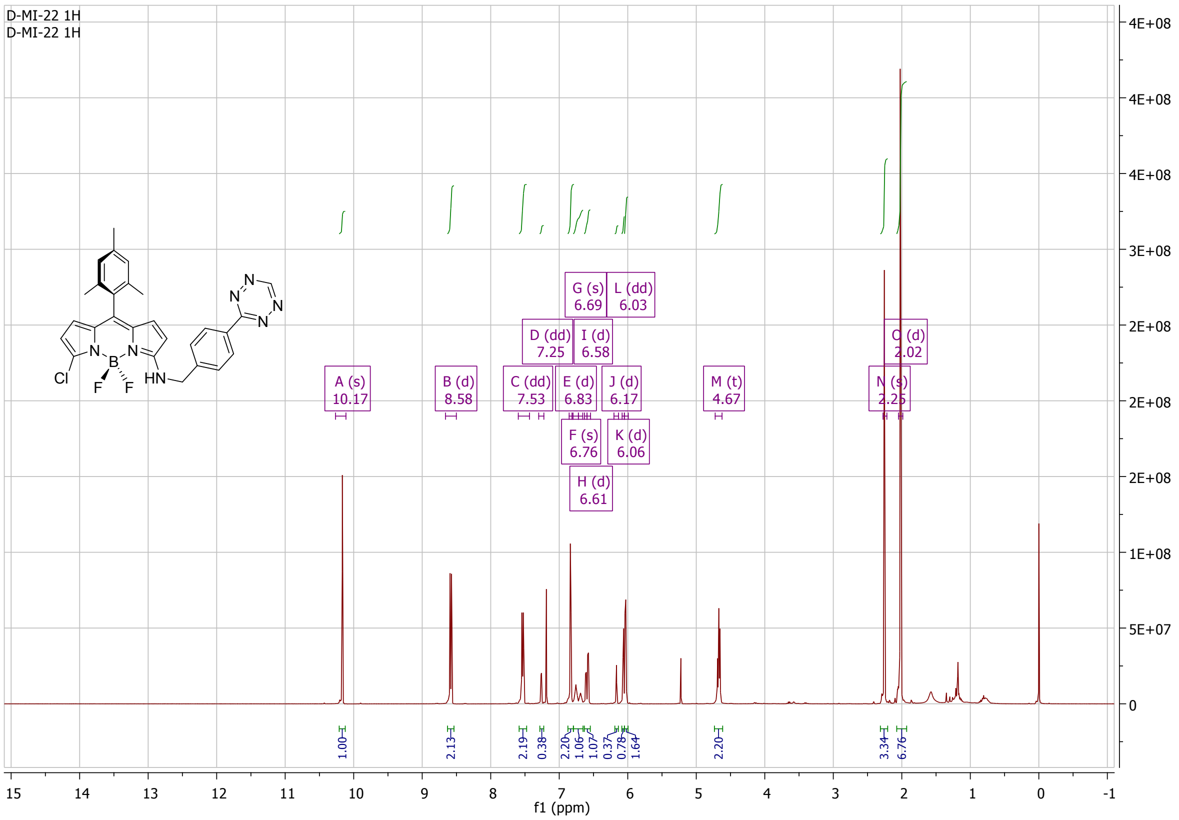

Figure 60S. ^{1}H NMR spectrum (400 MHz) of **2TzH** in $CDCl_3$.

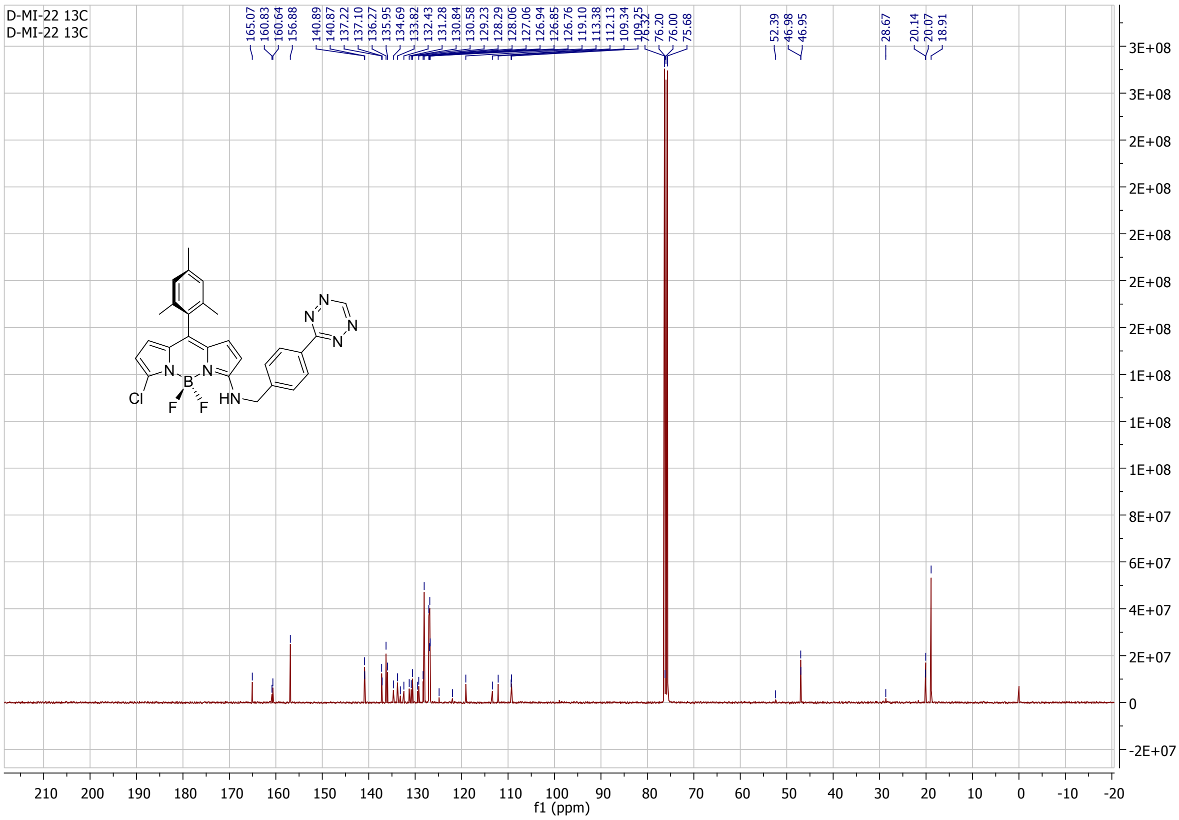

Figure 61S. $^{13}C\{^{1}H\}$ NMR spectrum (101 MHz) of **2TzH** in $CDCl_3$.

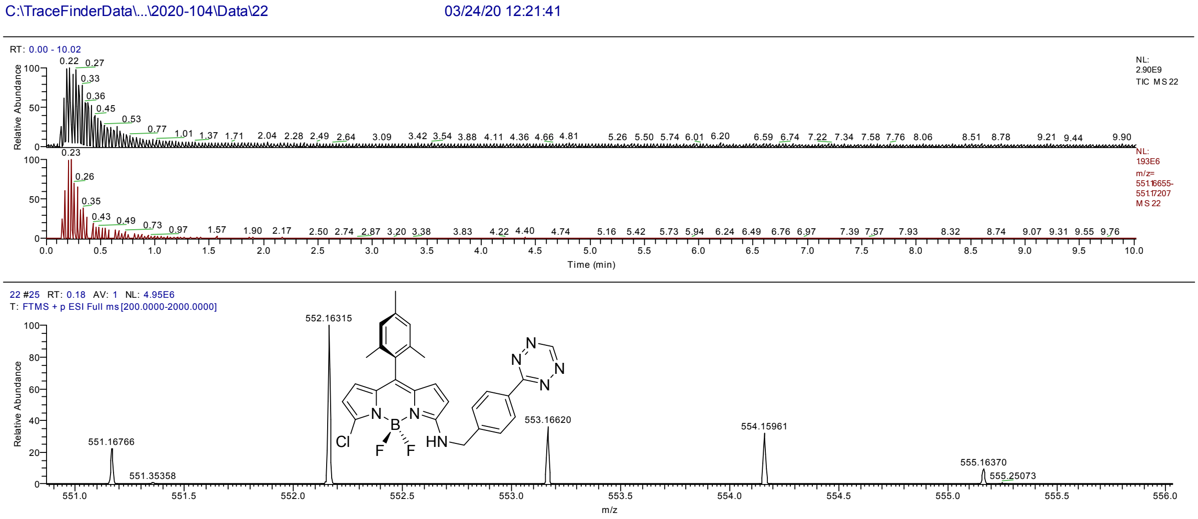

Figure 62S. HRMS (ESI positive) spectrum of **2TzH**.

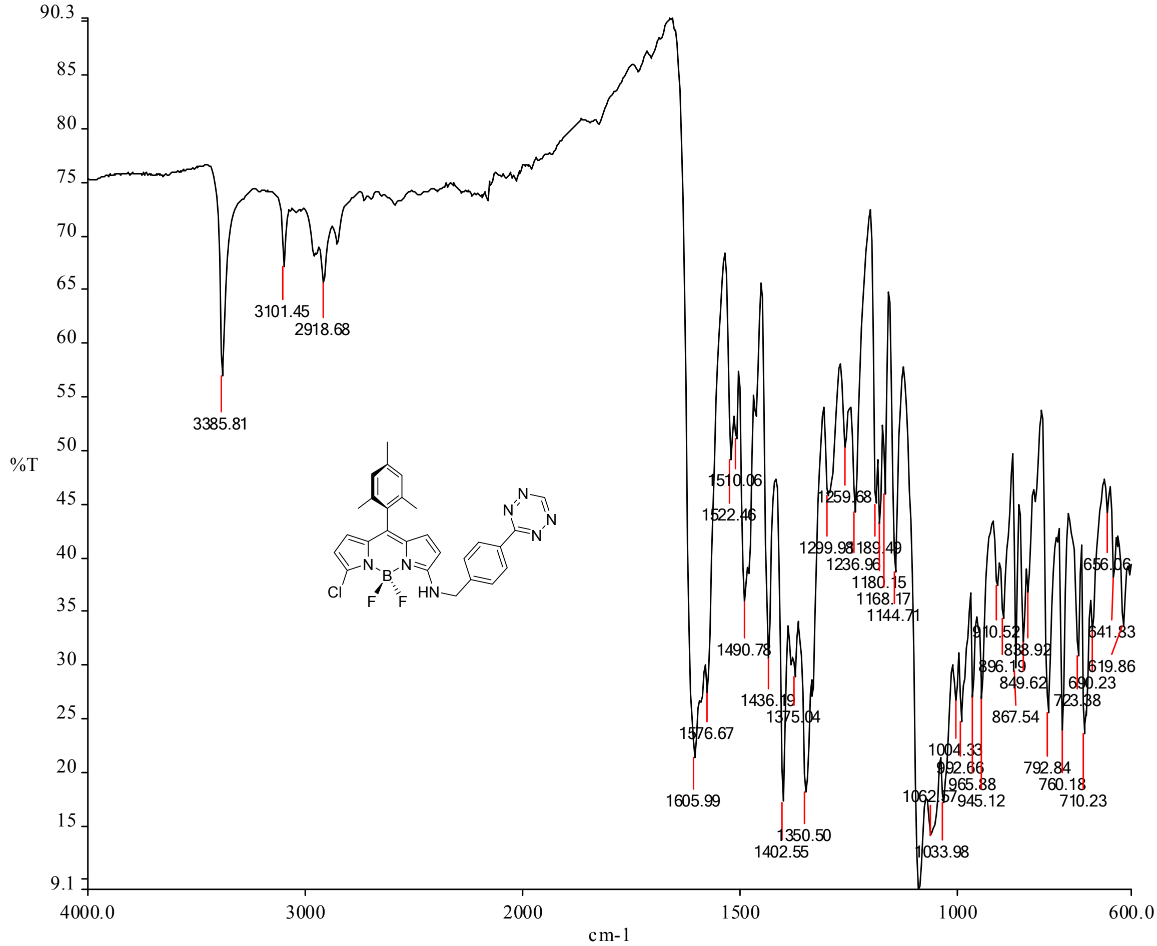

Figure 63S. IR (neat) spectrum of **2TzH**.

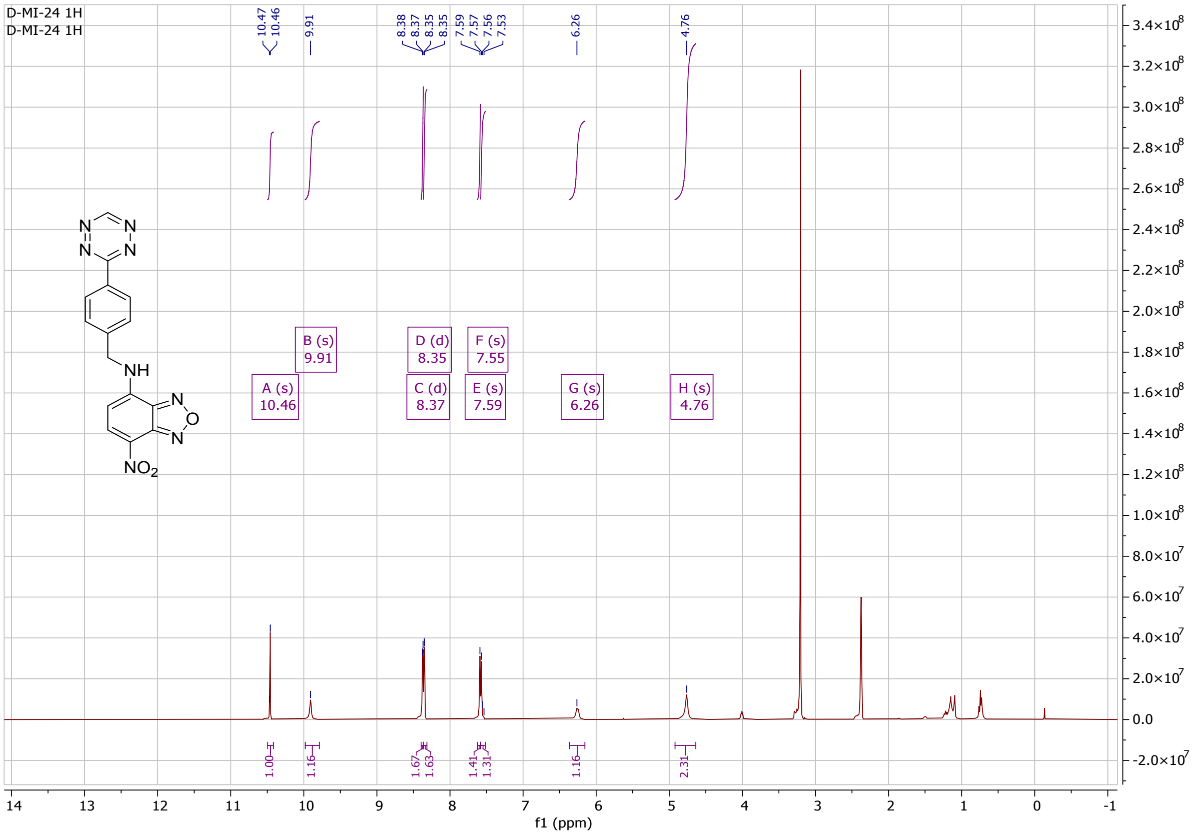

Figure 64S. ^{1}H NMR spectrum (400 MHz) of **3TzH** in DMSO-d_6.

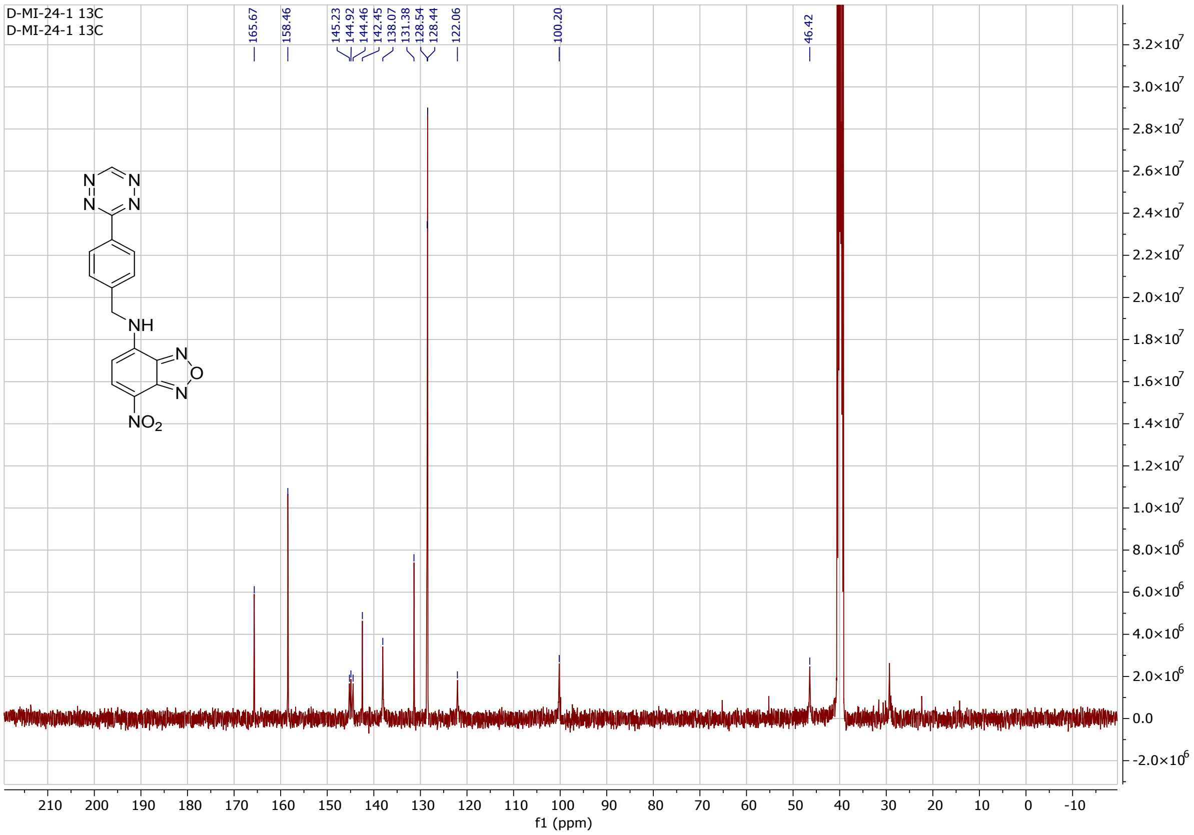

Figure 65S. ^{13}C{^{1}H} NMR spectrum (101 MHz) of **3TzH** in DMSO-d_6.

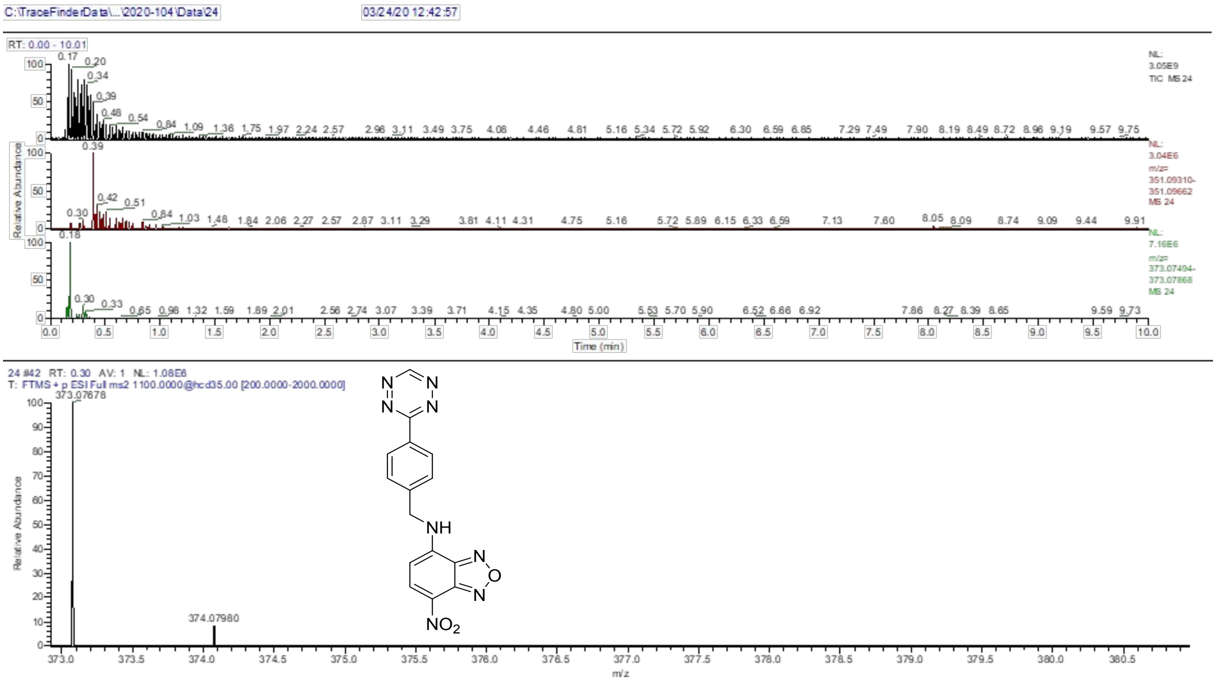

Figure 66S. HRMS (ESI positive) spectrum of **3TzH**.

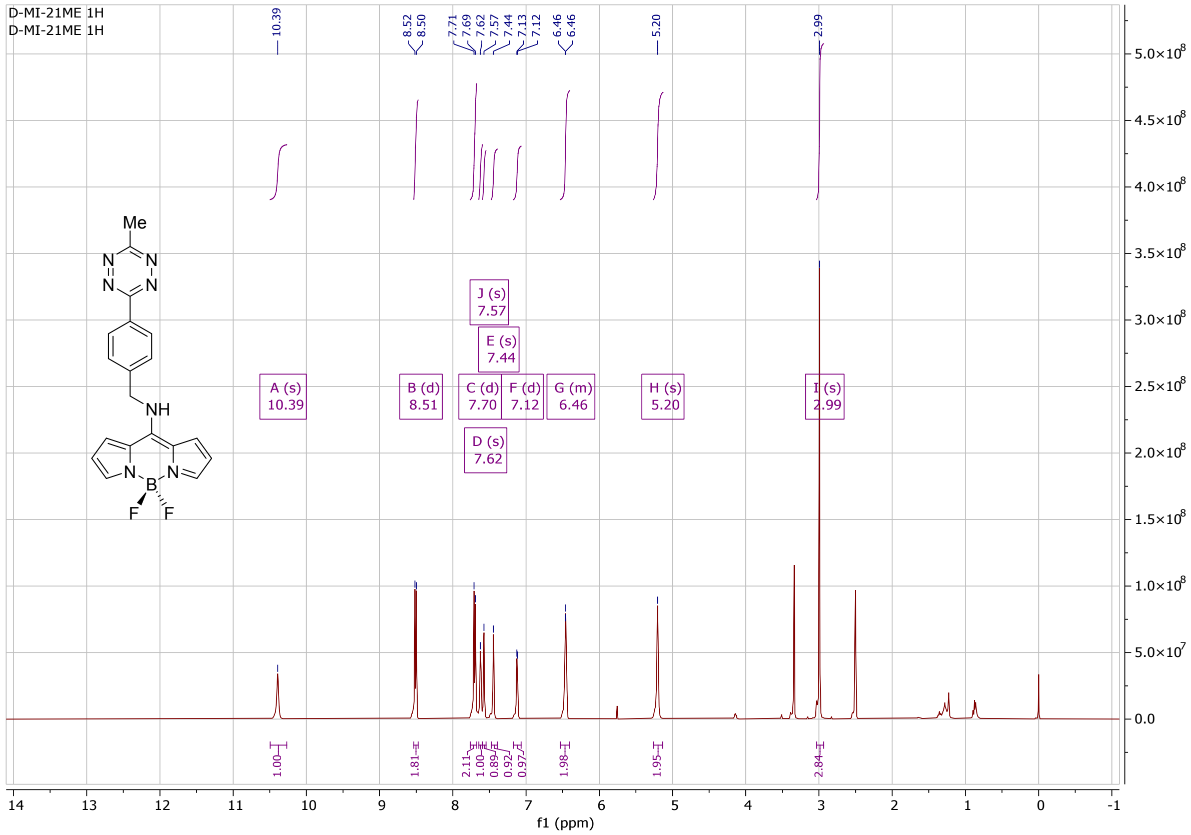

Figure 67S. ^{1}H NMR spectrum (400 MHz) of **1TzMe** in DMSO-d_6.

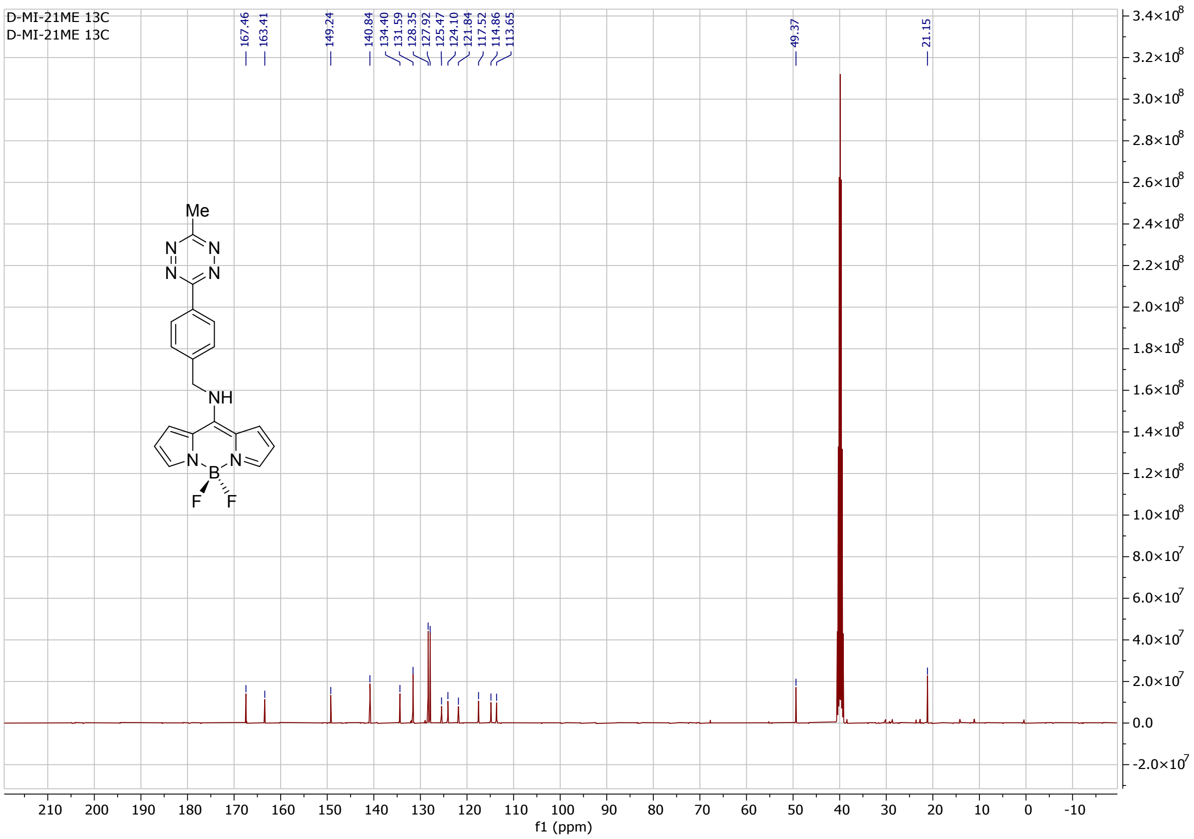

Figure 68S. $^{13}C\{^{1}H\}$ NMR spectrum (101 MHz) of **1TzMe** in DMSO-d_6.

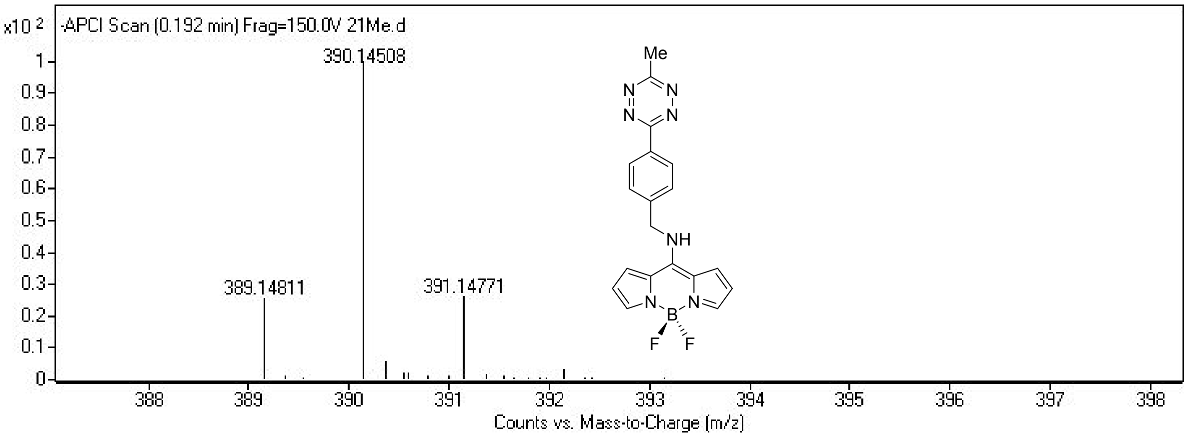

Figure 69S. HRMS (APCI negative) spectrum of **1TzMe**.

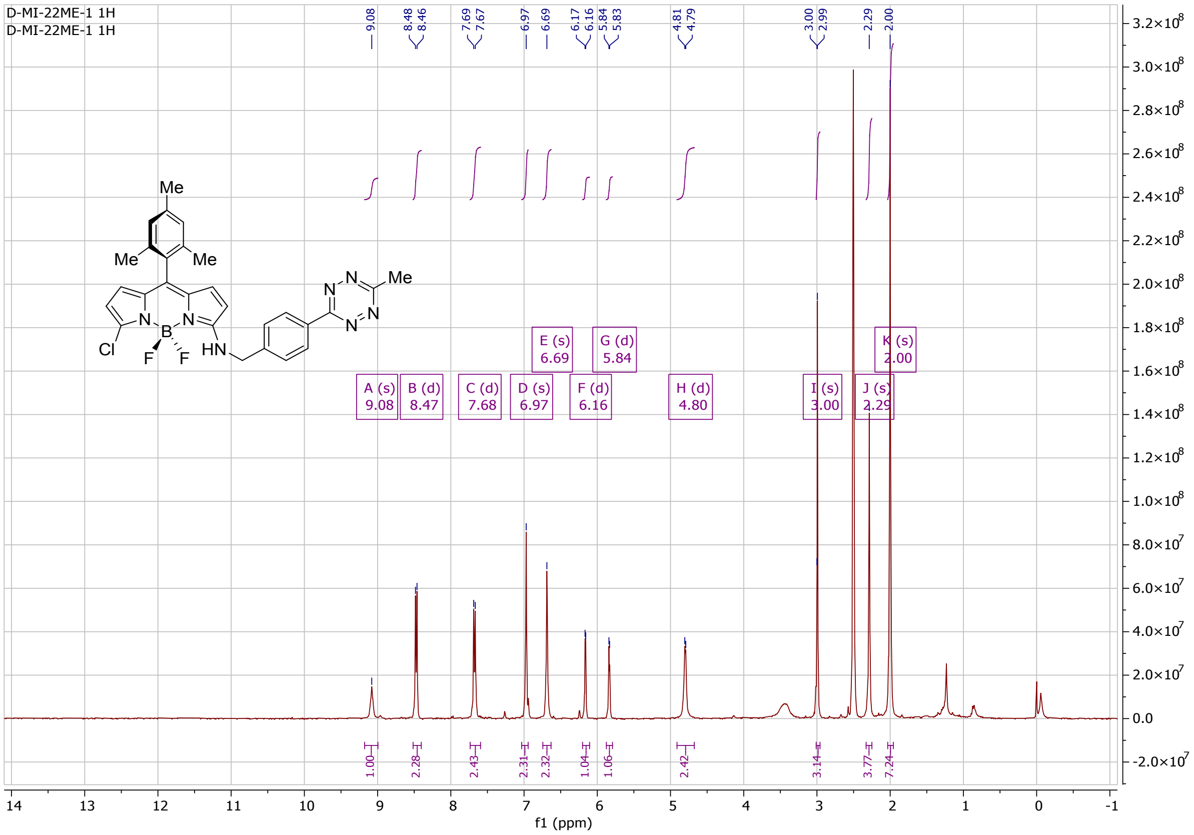

Figure 70S. ^{1}H NMR spectrum (400 MHz) of **2TzMe** in DMSO-d_6.

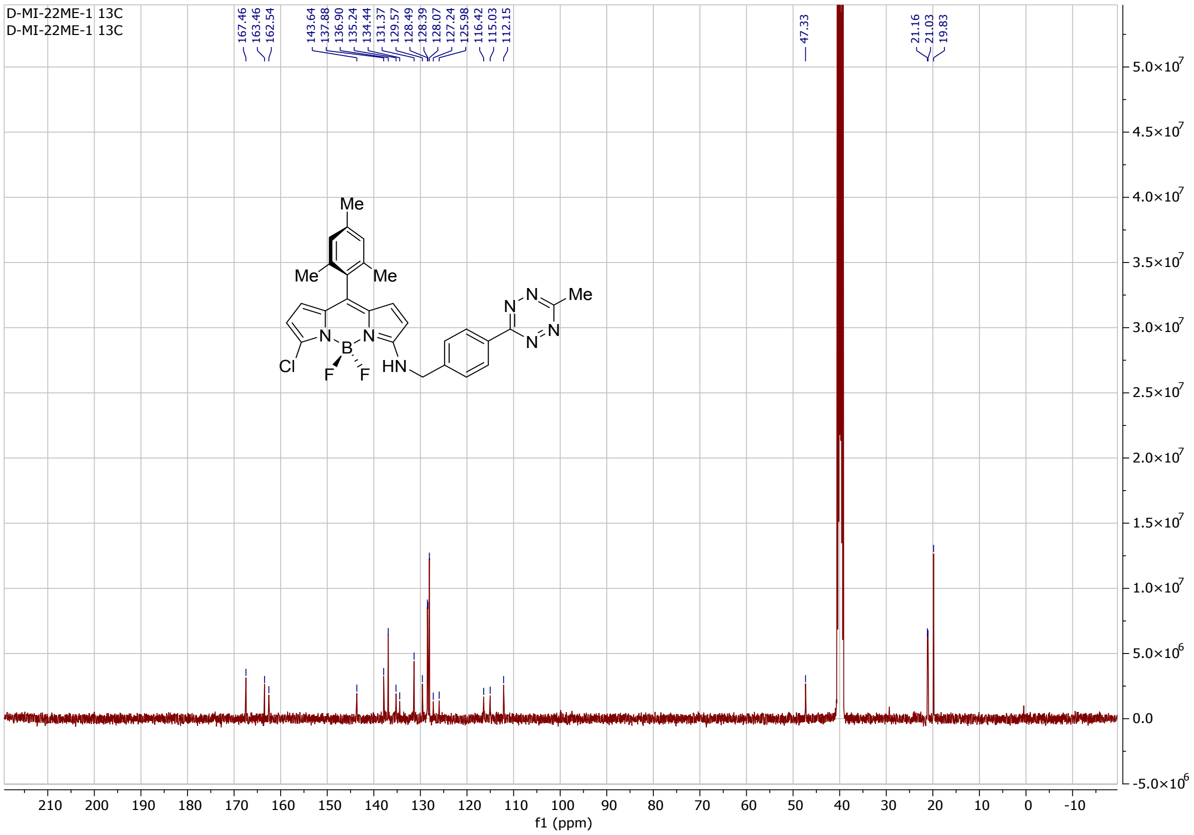

Figure 71S. $^{13}C\{^{1}H\}$ NMR spectrum (101 MHz) of **2TzMe** in DMSO-d_6.

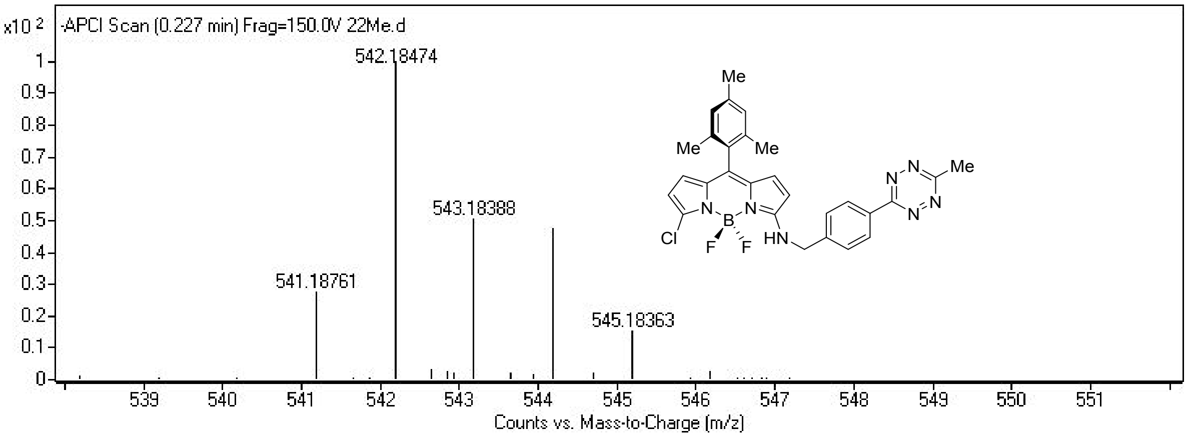

Figure 72S. HRMS (APCI negative) spectrum of **2TzMe**.

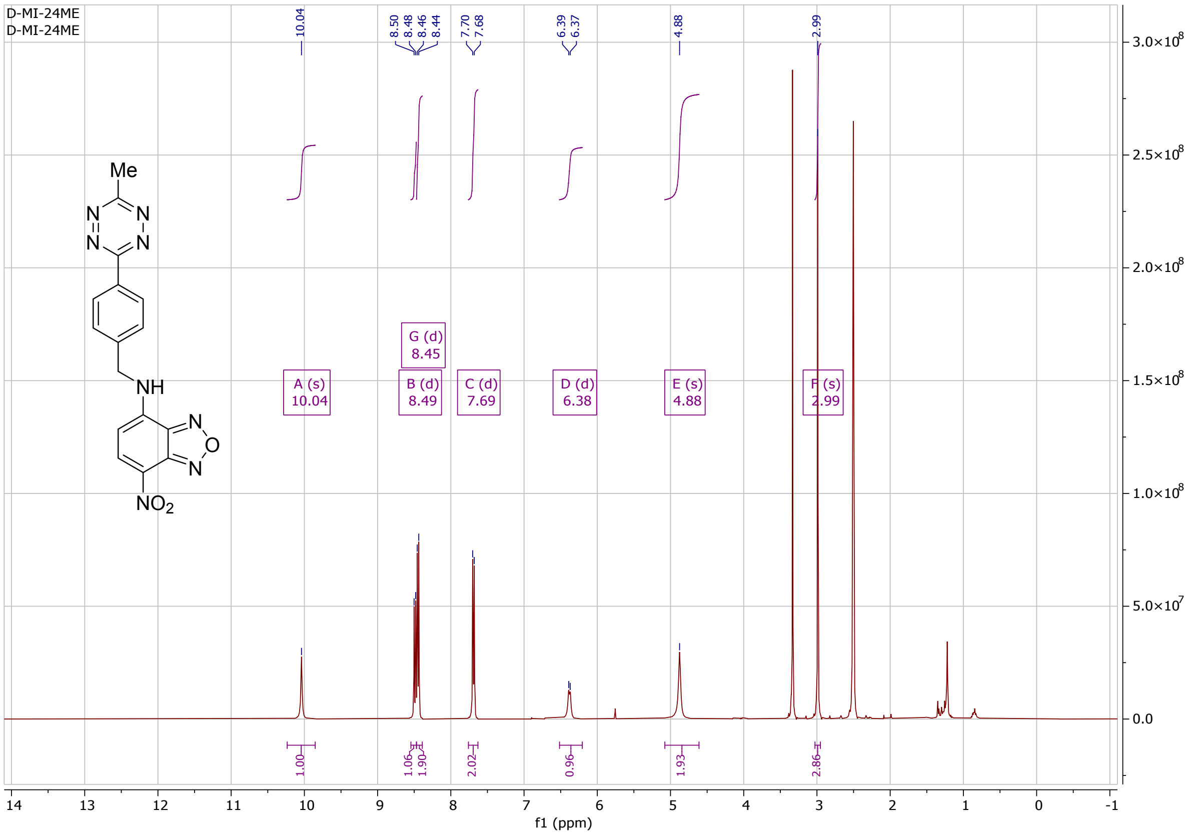

Figure 73S. ^{1}H NMR spectrum (400 MHz) of **3TzMe** in DMSO-d_6.

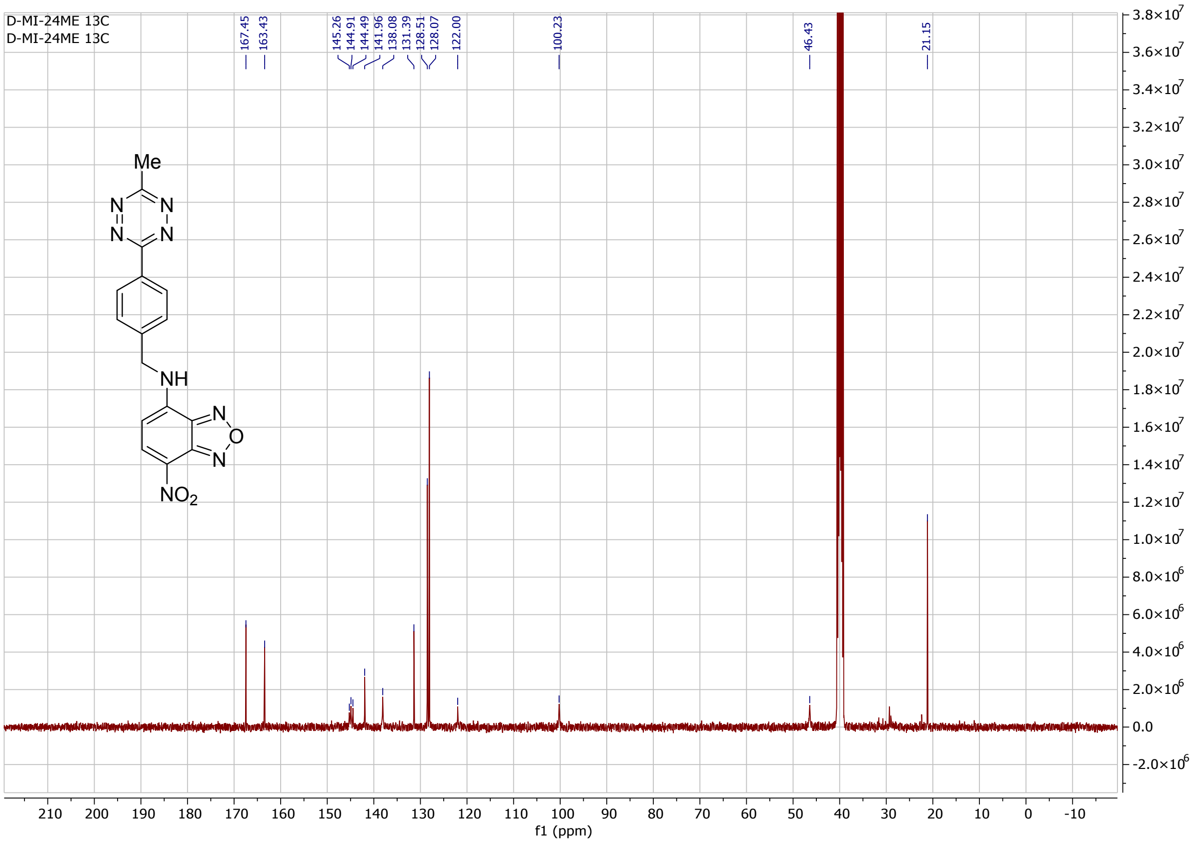

Figure 74S. $^{13}C\{^{1}H\}$ NMR spectrum (101 MHz) of **3TzMe** in DMSO-d_6.

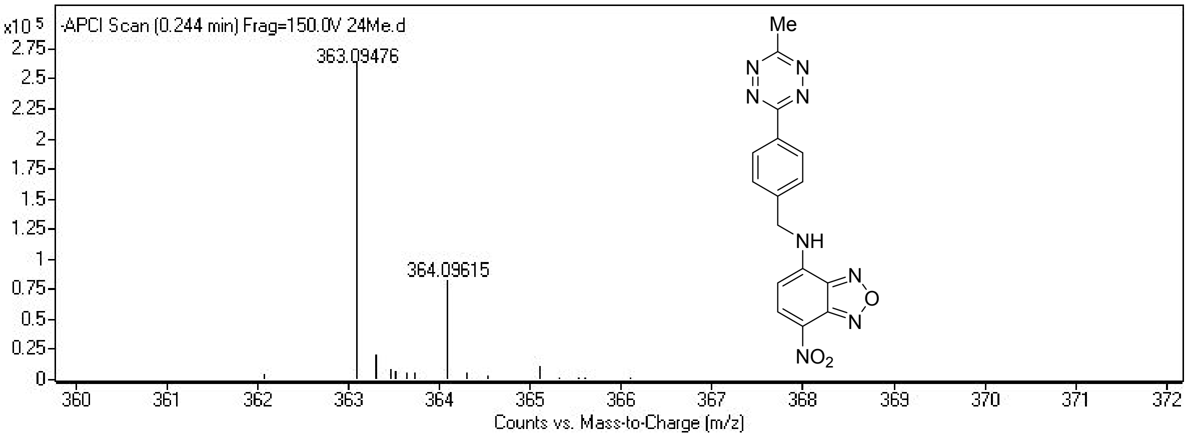

Figure 75S. HRMS (APCI negative) spectrum of **3TzMe**.

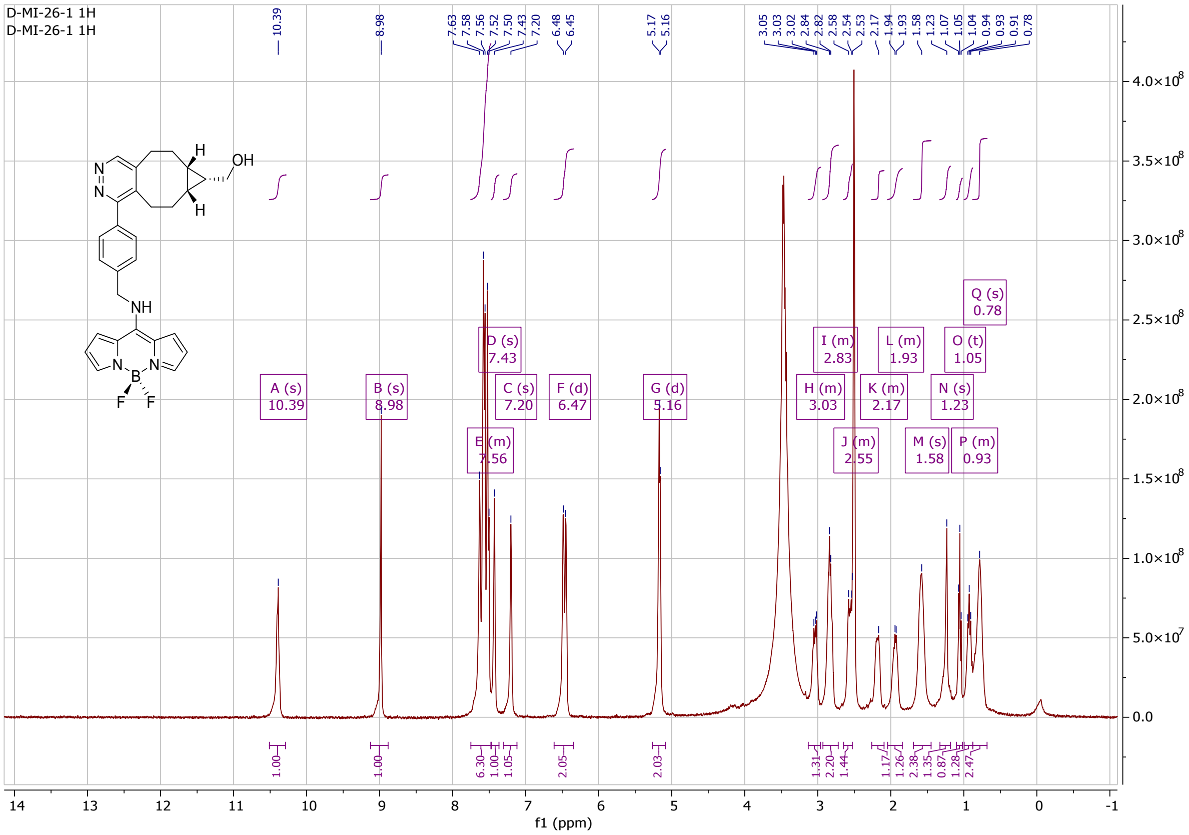

Figure 76S. ^{1}H NMR spectrum (400 MHz) of **1TzH•BCN** in DMSO-d_6.

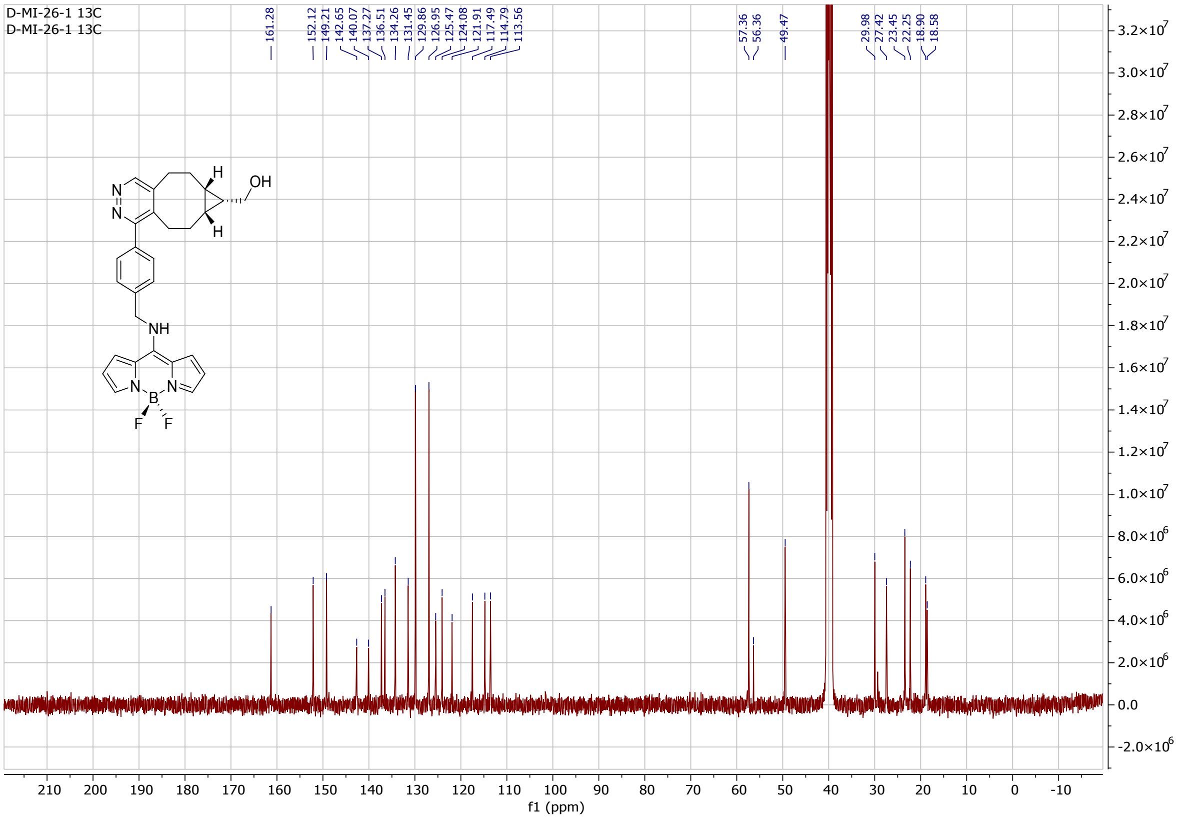

Figure 77S. $^{13}C\{^{1}H\}$ NMR spectrum (101 MHz) of **1TzH•BCN** in DMSO-d_6.

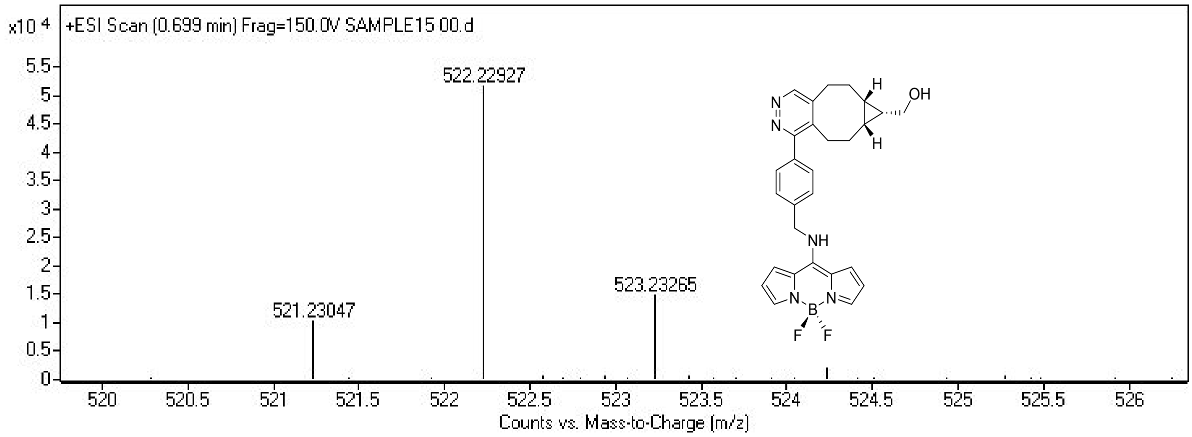

Figure 78S. HRMS (ESI positive) spectrum of **1TzH•BCN**.

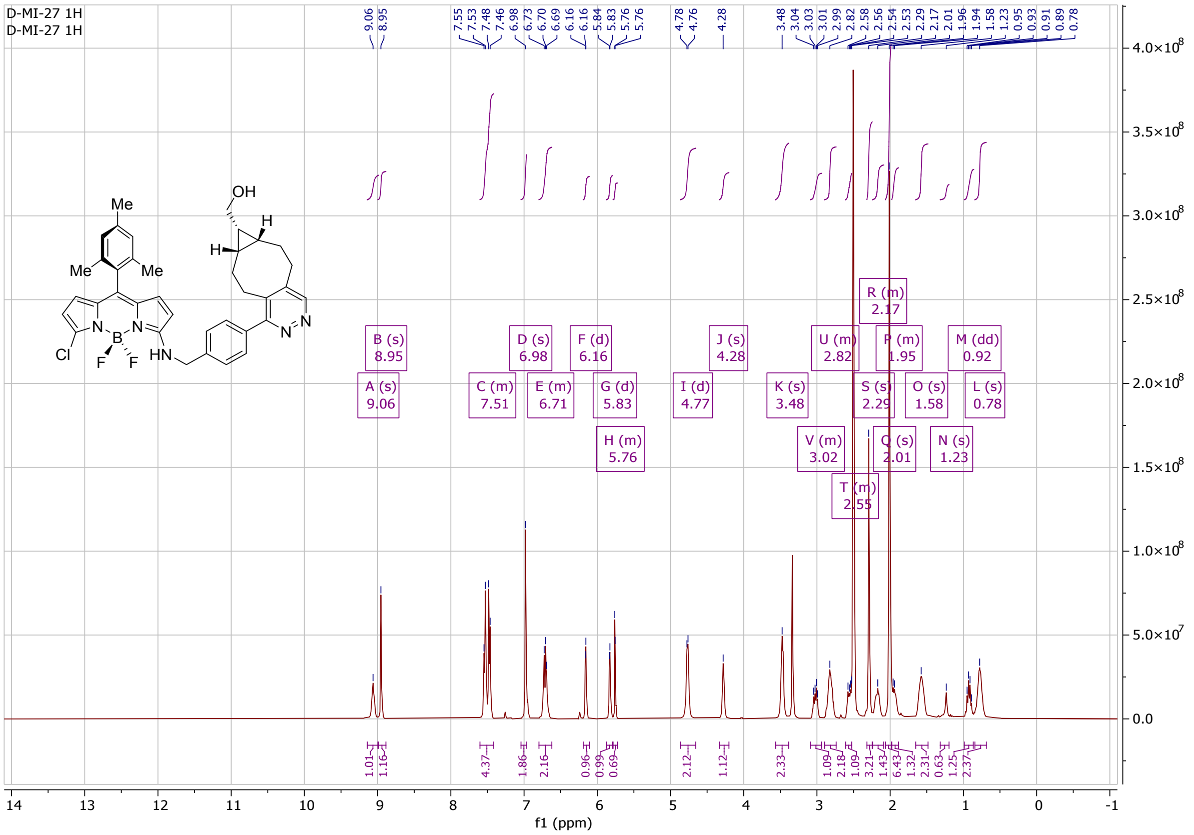

Figure 79S. ^{1}H NMR spectrum (400 MHz) of **2TzH•BCN** in DMSO-d_6.

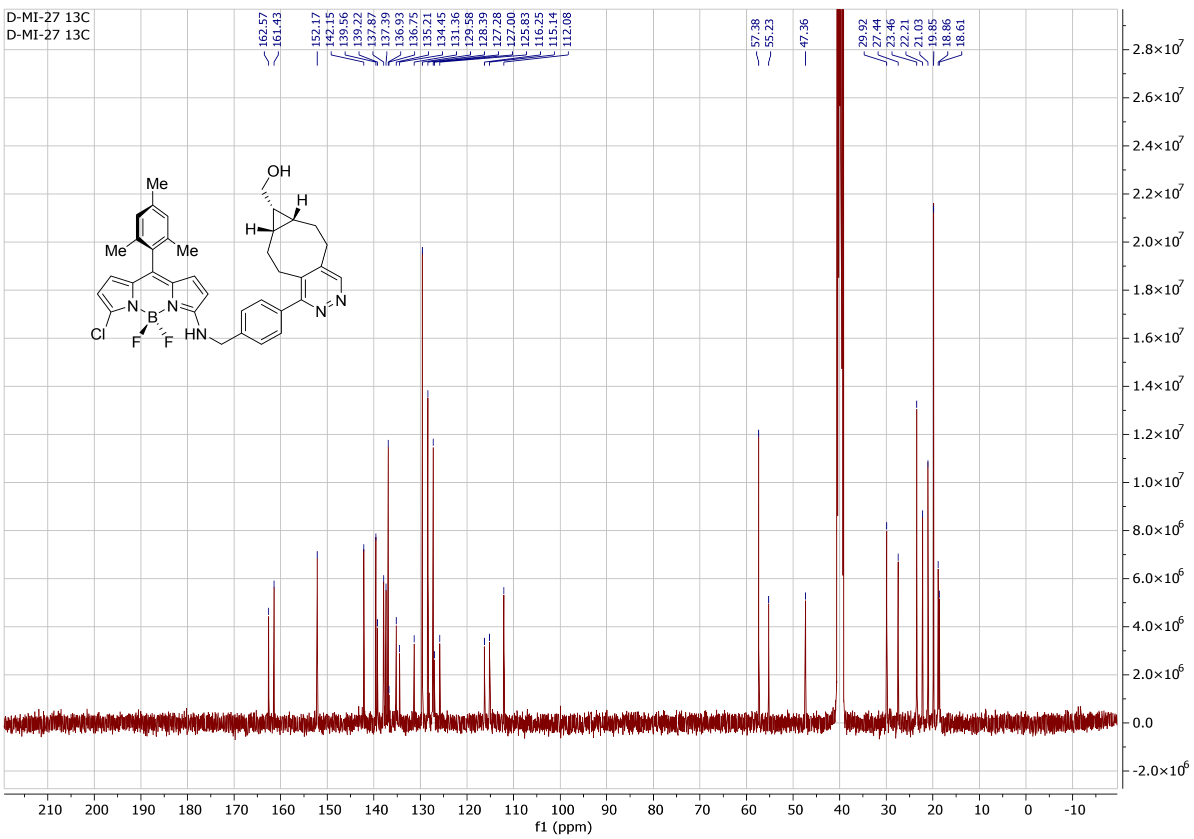

Figure 80S. $^{13}C\{^{1}H\}$ NMR spectrum (101 MHz) of **2TzH•BCN** in DMSO-d_6.

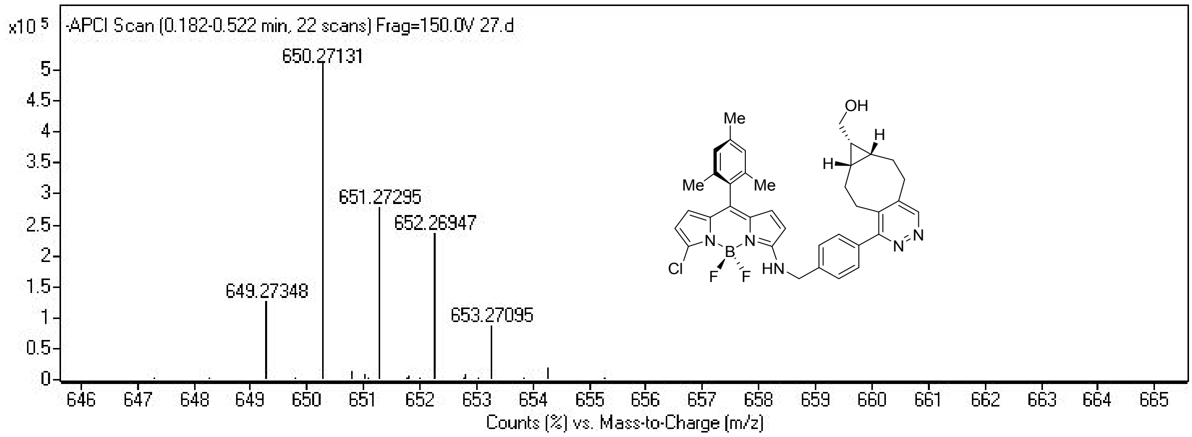

Figure 81S. HRMS (APCI negative) spectrum of **2TzH•BCN**.

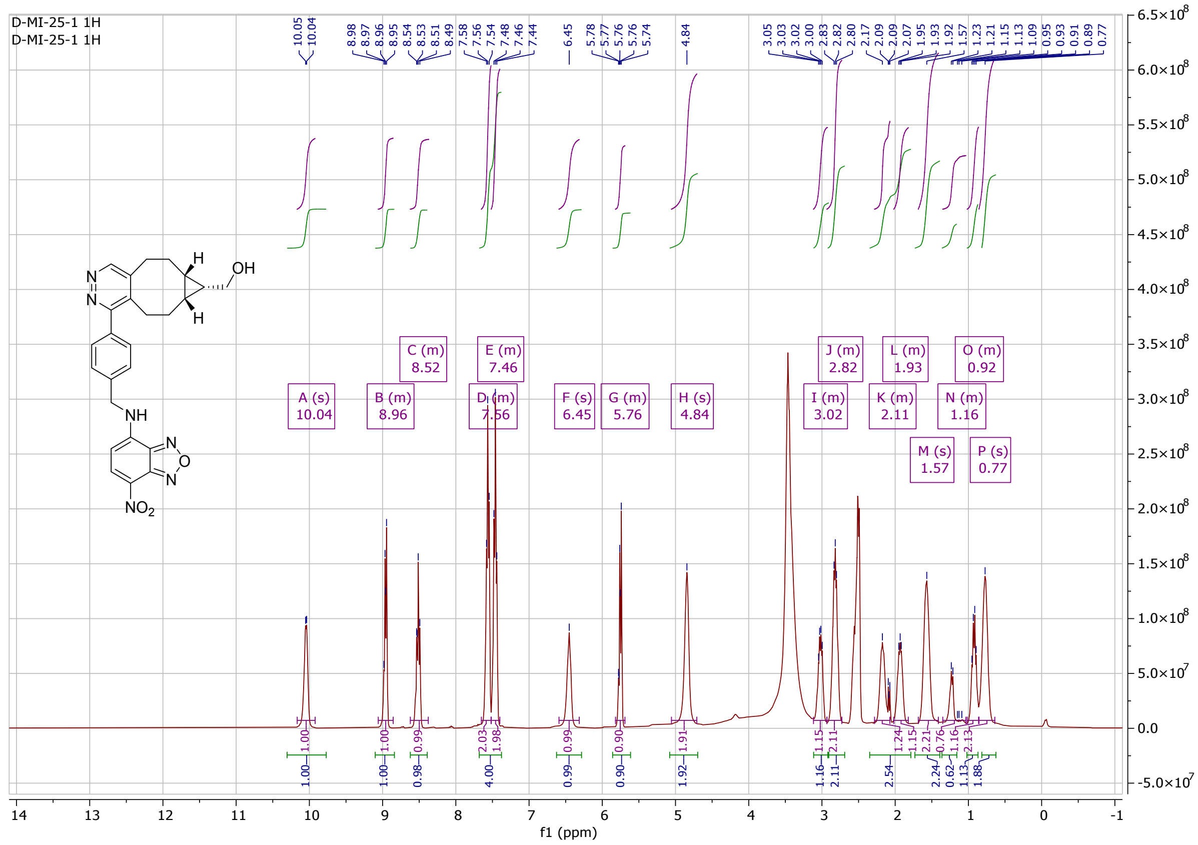

Figure 82S. ^{1}H NMR spectrum (400 MHz) of **3TzH•BCN** in DMSO-d_6.

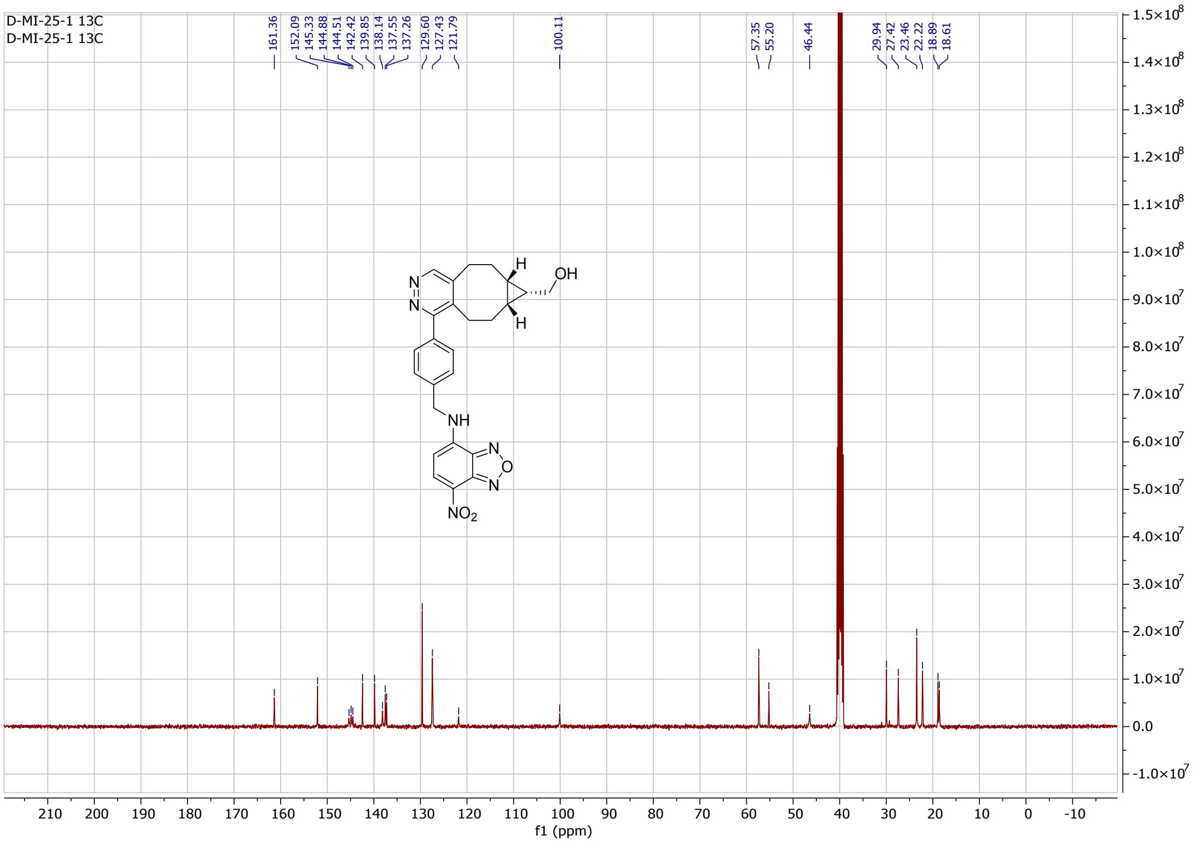

Figure 83S. $^{13}C\{^{1}H\}$ NMR spectrum (101 MHz) of **3TzH•BCN** in DMSO-d_6.

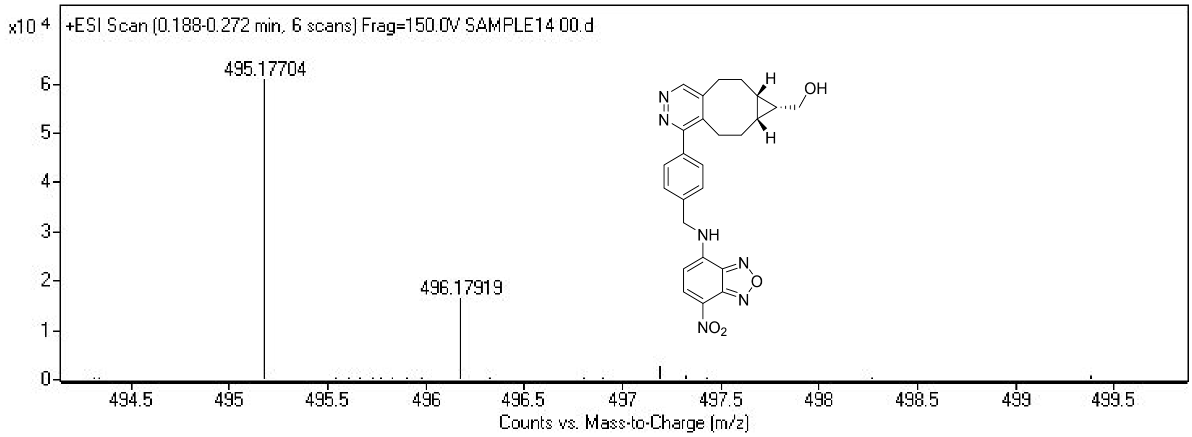

Figure 84S. HRMS (ESI positive) spectrum of **3TzH•BCN**.

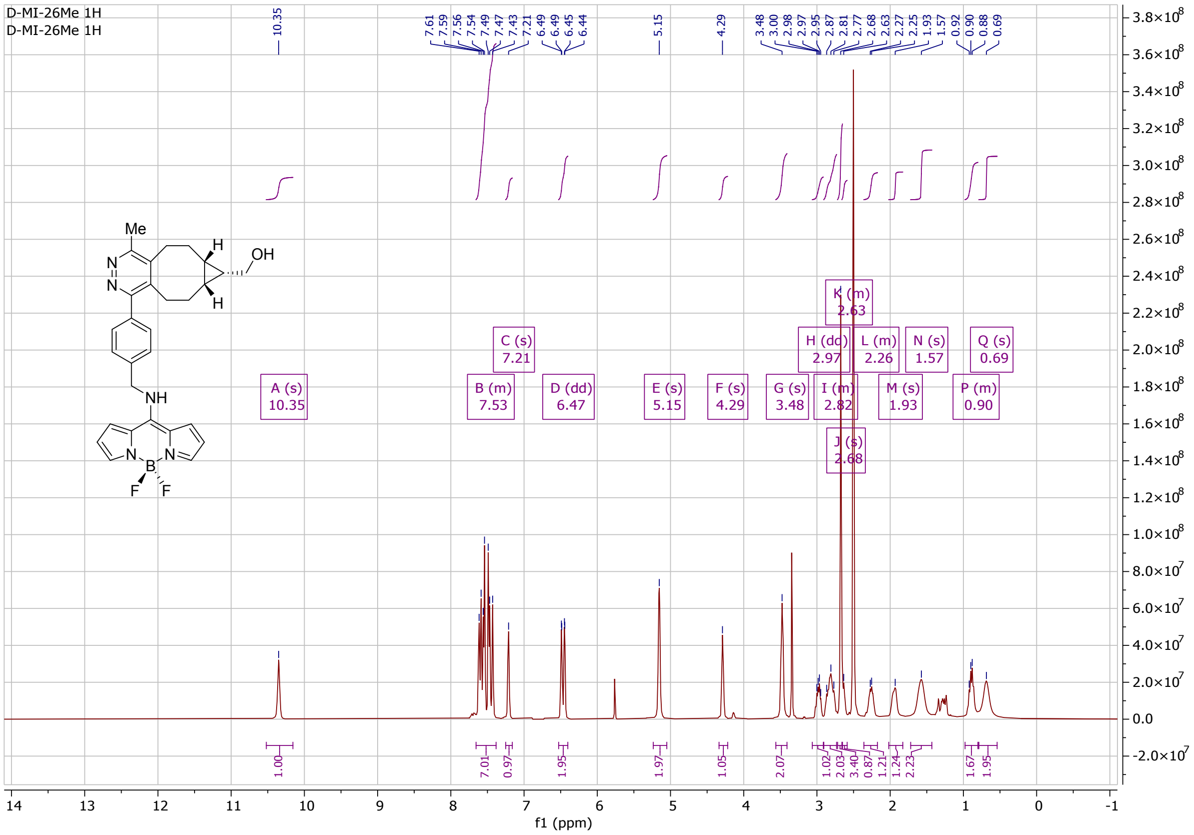

Figure 85S. ^{1}H NMR spectrum (400 MHz) of **1TzMe•BCN** in DMSO-d_6.

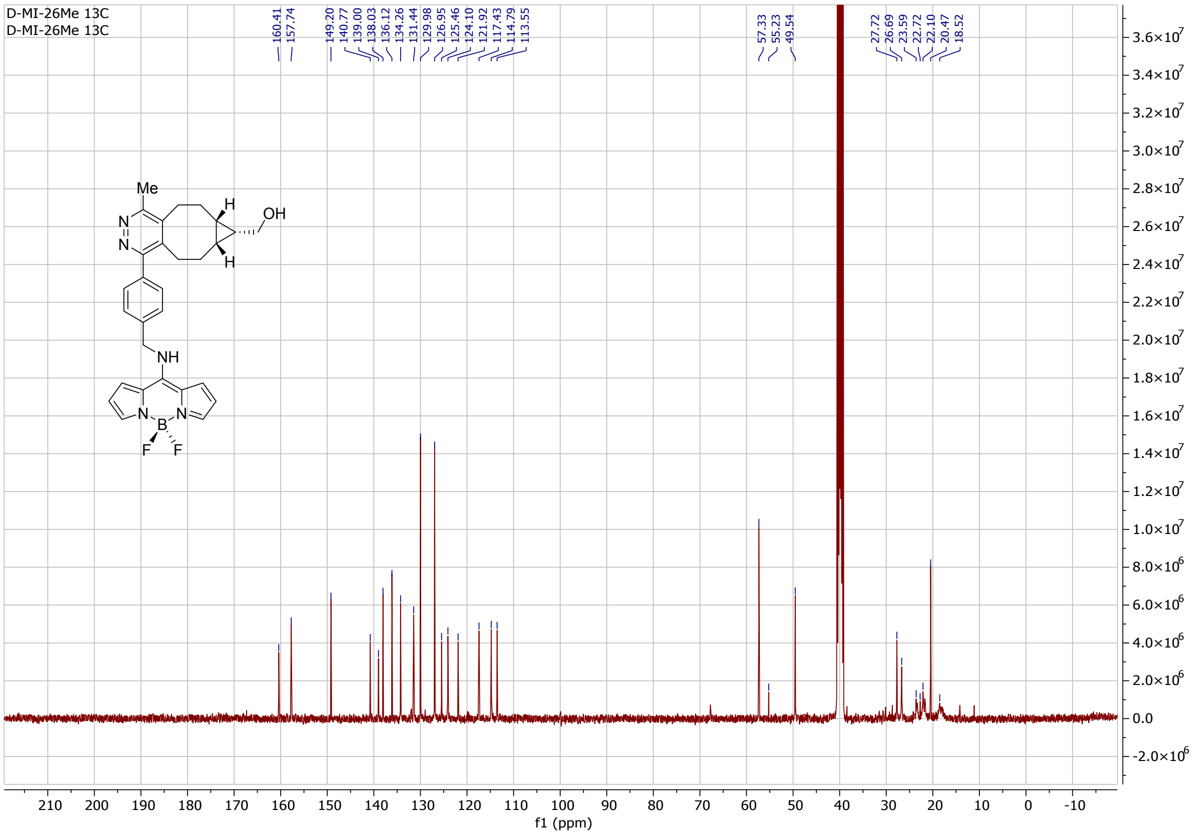

Figure 86S. ^{13}C{^{1}H} NMR spectrum (101 MHz) of **1TzMe•BCN** in DMSO-d_6.

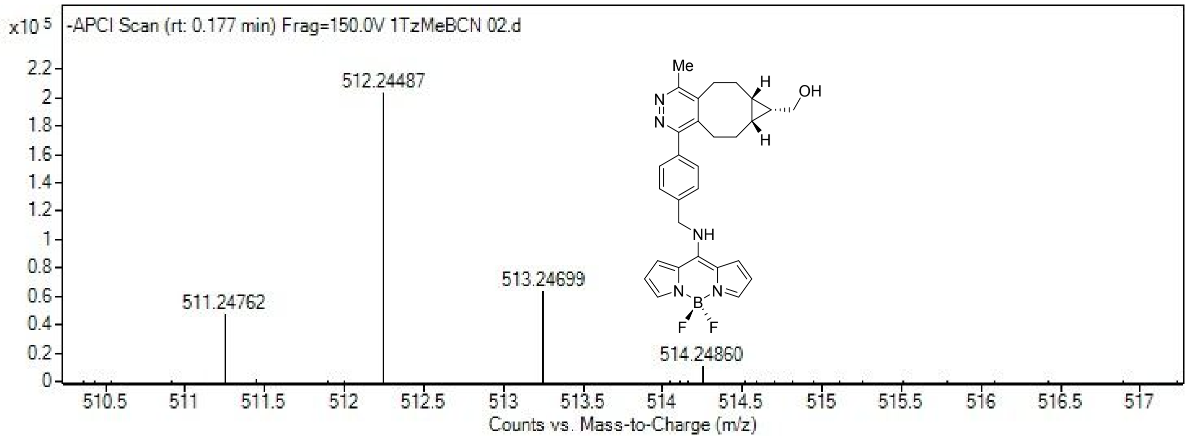

Figure 87S. HRMS (APCI negative) spectrum of **1TzMe•BCN**.

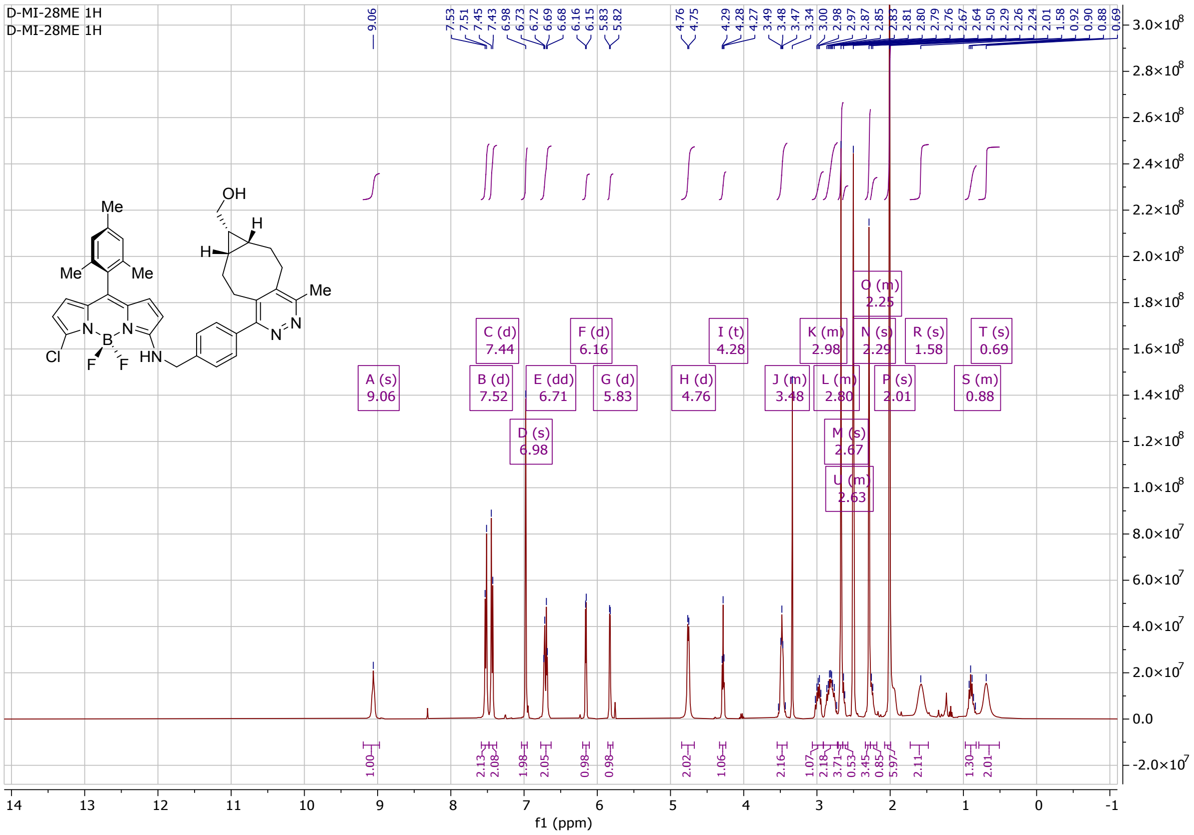

Figure 88S. ^{1}H NMR spectrum (400 MHz) of **2TzMe•BCN** in DMSO-d_6.

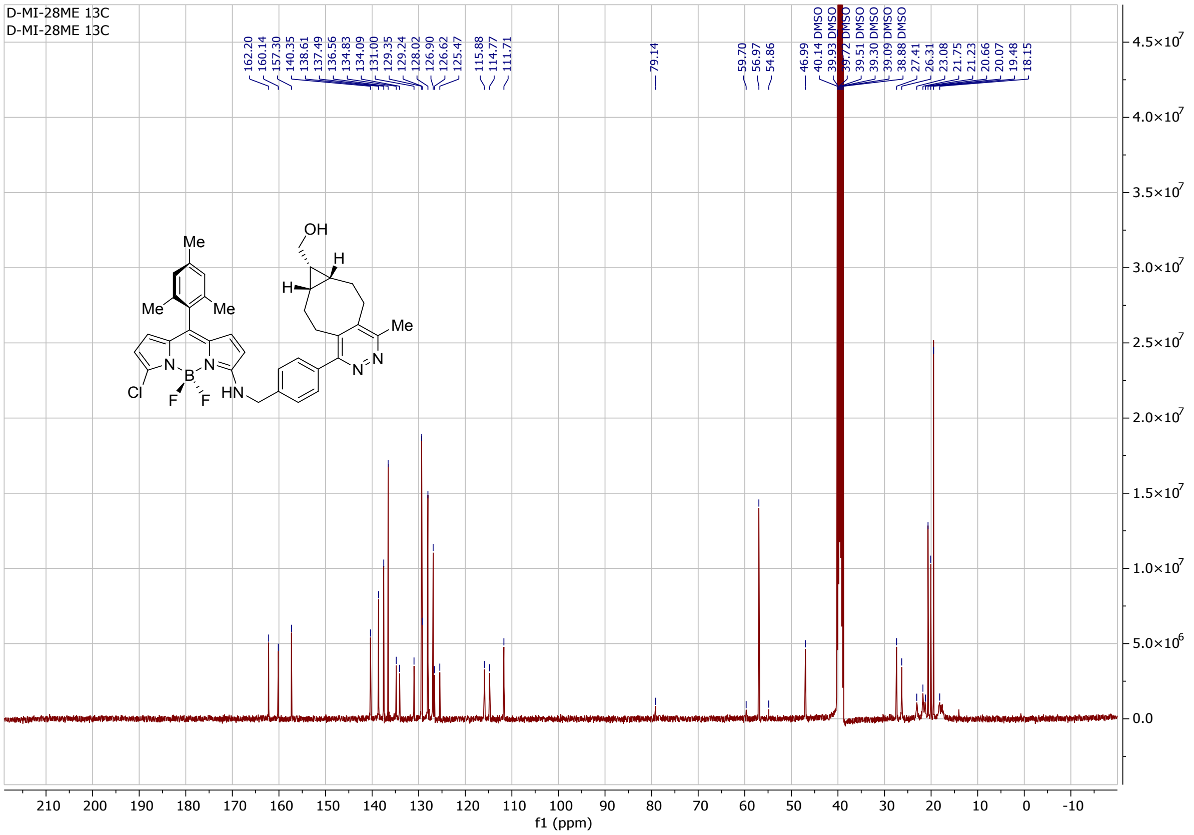

Figure 89S. $^{13}C\{^{1}H\}$ NMR spectrum (101 MHz) of **2TzMe•BCN** in DMSO-d_6.

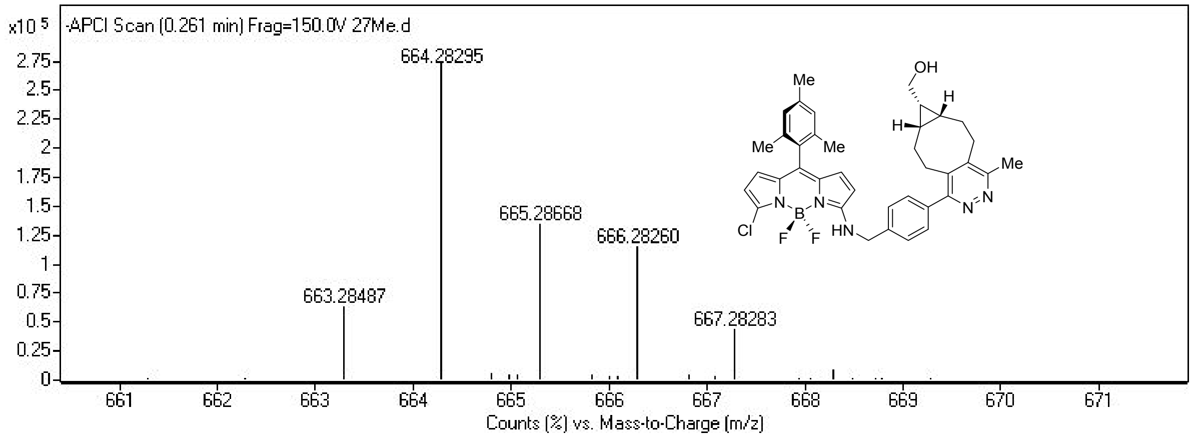

Figure 90S. HRMS (APCI negative) spectrum of **2TzMe•BCN**.

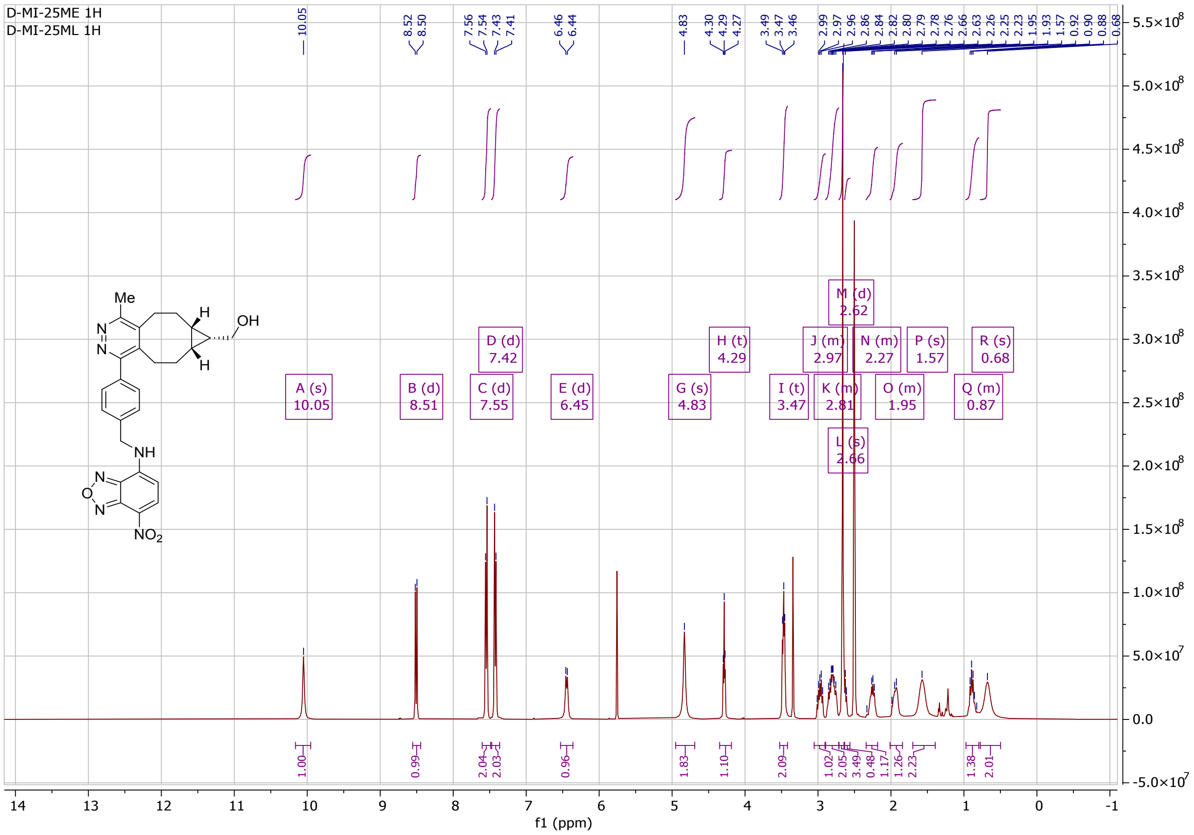

Figure 91S. ^{1}H NMR spectrum (400 MHz) of **3TzMe•BCN** in DMSO-d_6.

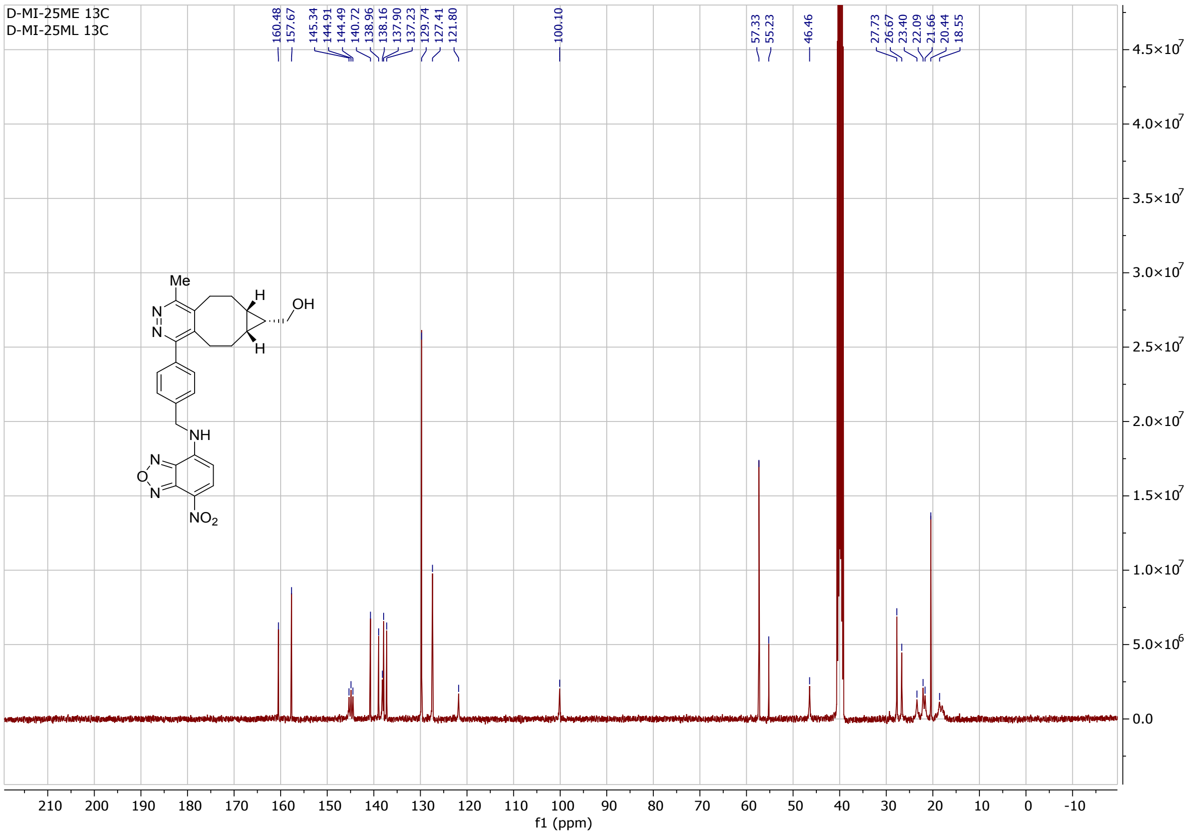

Figure 92S. ^{13}C{^{1}H} NMR spectrum (101 MHz) of **3TzMe•BCN** in DMSO-d_6.

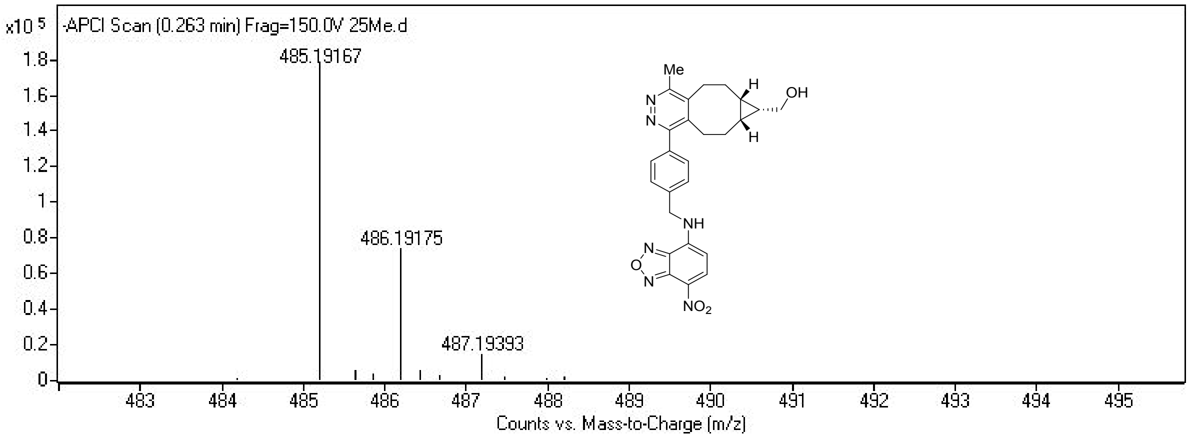

Figure 93S. HRMS (APCI negative) spectrum of **3TzMe•BCN**.

References

(1) Goud, T. V.; Tutar, A.; Biellmann, J.-F. Synthesis of 8-heteroatom-substituted 4,4-difluoro-4-bora-3a,4a-diaza-s-indacene dyes (BODIPY). *Tetrahedron* **2006**, *62*, 5084−5091.

(2) Plater, M. J.; Aiken, S.; Bourhill, G. A new synthetic route to donor–acceptor porphyrins. *Tetrahedron* **2002**, *58*, 2405–2413.

(3) Leen, V.; Yuan, P.; Wang, L.; Boens, N.; Dehaen, W. Synthesis of *Meso*-Halogenated BODIPYs and Access to *Meso*-Substituted Analogues. *Org. Lett.* **2012**, *14*, 6150–6153.

(4) Rohand, T.; Dolusic, E.; Ngo, T. H.; Maes, W.; Dehaen, W. Efficient Synthesis of Aryldipyrromethanes in Water and Their Application in the Synthesis of Corroles and Dipyrromethenes. *Arkivoc* **2007**, *2007*, 307–324.

(5) Domaille, D. W.; Zeng, L.; Chang, C. J. Visualizing Ascorbate-Triggered Release of Labile Copper within Living Cells using a Ratiometric Fluorescent Sensor. *J. Am. Chem. Soc.* **2010**, *132*, 1194–1195.

(6) Zhou, X.; Yu, C.; Feng, Z.; Yu, Y.; Wang, J.; Hao, E.; Wei, Y.; Mu, X.; Jiao, L. Highly regioselective α-chlorination of the BODIPY chromophore with copper(II) chloride. *Org. Lett.* **2015**, *17*, 4632–4635.

(7) Isaacman, M. J.; Corigliano, E. M.; Theogarajan, L. S. Stealth Polymeric Vesicles via Metal-Free Click Coupling. *Biomacromolecules* **2013**, *14*, 2996–3000.

(8) Porrès, L.; Holland, A.; Pålsson, L.-O.; Monkman, A. P.; Kemp, C.; Beeby, A. Absolute Measurements of Photoluminescence Quantum Yields of Solutions Using an Integrating Sphere. *J. Fluoresc.* **2006**, *16*, 267–273.

(9) Rurack, K.; Spieles, M. Fluorescence Quantum Yields of a Series of Red and Near-Infrared Dyes Emitting at 600-1000 nm. *Anal. Chem.* **2011**, *83*, 1232–1242.

(10) Sunahara, H.; Urano, Y.; Kojima, H.; Nagano, T. Design and Synthesis of a Library of BODIPY-Based Environmental Polarity Sensors Utilizing Photoinduced Electron-Transfer-Controlled Fluorescence ON/OFF Switching. *J. Am. Chem. Soc.* **2007**, *129*, 5597–5604.
(11) Shum, J.; Zhang, P.-Z.; Lee, L. C-C.; Lo, K. K-W. Bioorthogonal Phosphorogenic Rhenium(I) Polypyridine Sydnone Complexes for Specific Lysosome Labeling. *ChemPlusChem* **2020**, *85*, 1374–1378.
